# Biocatalytic production of bicyclic β-lactams with three contiguous chiral centres using engineered crotonases

**DOI:** 10.1038/s42004-018-0106-z

**Published:** 2019-01-24

**Authors:** Refaat B. Hamed, J. Ruben Gomez-Castellanos, Luc Henry, Sven Warhaut, Timothy D. W. Claridge, Christopher J. Schofield

**Affiliations:** 1 Chemistry Research Laboratory, Department of Chemistry, University of Oxford, Mansfield Road, Oxford, OX1 3TA UK; 2 Present Address: Department of Pharmacognosy, Faculty of Pharmacy, Assiut University, Assiut, 71256 Egypt; 3 Present Address: School of Chemistry and Biosciences, University of Bradford, Richmond Road, Bradford, BD7 1DP UK

**Keywords:** Chemical biology, Biocatalysis, Biosynthesis

## Abstract

There is a need to develop asymmetric routes to functionalised β-lactams, which remain the most important group of antibacterials. Here we describe biocatalytic and protein engineering studies concerning carbapenem biosynthesis enzymes, aiming to enable stereoselective production of functionalised carbapenams with three contiguous chiral centres. Structurally-guided substitutions of wildtype carboxymethylproline synthases enable tuning of their C-N and C-C bond forming capacity to produce 5-carboxymethylproline derivatives substituted at C-4 and C-6, from amino acid aldehyde and malonyl-CoA derivatives. Use of tandem enzyme incubations comprising an engineered carboxymethylproline synthase and an alkylmalonyl-CoA forming enzyme (i.e. malonyl-CoA synthetase or crotonyl-CoA carboxylase reductase) can improve stereocontrol and expand the product range. Some of the prepared 4,6-disubstituted-5-carboxymethylproline derivatives are converted to bicyclic β-lactams by carbapenam synthetase catalysis. The results illustrate the utility of tandem enzyme systems involving engineered crotonases for asymmetric bicyclic β-lactam synthesis.

## Abstract (web summary)

Beta-lactams are important antiobiotics but synthesising functionalised derivatives in high enantiomeric purity can be challenging. Here malonyl-CoA derivatives are applied in an enantioselective multi-enzyme cascade, yielding beta-lactams bearing three contiguous chiral centres in high diastereomeric purity.

## Introduction

**Fig. 1:**
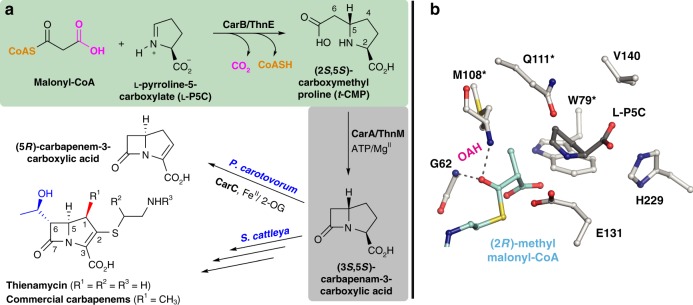
Enzymes involved in carbapenem biosynthesis. **a** Proposed roles of carboxymethylproline synthases (CarB and ThnE, green box) and β-lactam synthetases (CarA and ThnM, grey box) in biosynthesis of (5*R*)-carbapenem-3-carboxylic acid and thienamycin, respectively^21^. Note the differences between thienamycin and clinically used carbapenems, notably the presence of a 1β-methyl group in most of the latter^7,8^; **b** View from a CarB crystal structure^16^, with (2*R*)-methylmalonyl-CoA and pyrroline-5-carboxylate (l-P5C) modelled into the active site, showing residues proposed to be important in catalysis (including oxyanion hole (OAH)-forming residues: Gly62_CarB_/Gly107_ThnE_ and Met108_CarB_/Val153_ThnE_). CarB residues marked with an asterisk (and analogous residues in ThnE (Supplementary Fig. 1)), were targeted for control of stereoselectivity in formation of C-4/C-6-substituted products

β-lactams are vital antibiotics and are finding new therapeutic applications^1–4^. Most bicyclic β-lactams (e.g. penicillins and cephalosporins) are produced by fermentation, or modification of fermentation-derived materials. Carbapenems, which are used for treatment of infections, including multidrug-resistant bacteria^5^, are an exception. Carbapenems, which have at least three chiral centres, are produced by synthesis with consequent cost implications and limitations on derivatives that can be produced. The carbapenem substitution pattern affects their activities and pharmacokinetic profiles^6^. All clinically used carbapenems have the (6*R*)-hydroxyethyl sidechain (Fig. 1a) and most of them are C-1 substituted, in order to increase potency and avoid hydrolysis by dehydropeptidases^7,8^. There is a need to develop efficient asymmetric routes for antibiotic production, where cost of goods is important. With a view to enabling routes to functionalised bicycle β-lactams, in particular C-1/C-6-functionalised bicyclic β-lactams as in carbapenems, we are investigating engineering of carbapenem biosynthesis enzymes^9–11^.

Three enzymes (CarA, B and C) are reported to catalyse biosynthesis of (5*R*)-carbapen-2-em-3-carboxylate (C3C) in *Pectobacterium carotovorum*, with multiple enzymes being involved in biosynthesis of thienamycin in *Streptomyces cattleya*^12^ (Fig. 1a). The formation of (2*S*,5*S*)-carboxymethylproline (*t*-CMP), from malonyl-CoA and pyroline-5-carboxylate (in equilibrium with l-glutamate semialdehyde/5-hydroxyproline, collectively l-GHP), as catalysed by CarB in *P. carotovorum*^13–16^ and ThnE in *S. cattleya*^17,18^, is proposed as a common step in both pathways. CarB and ThnE are carboxymethylproline synthases (CMPSs) of the crotonase superfamily^19,20^. Most crotonases employ an oxyanion hole (OAH) to stabilise an enolate intermediate, usually generated by decarboxylation of a malonyl-CoA derivative (Fig. 1b and Supplementary Fig. 1). CarB/ThnE-catalysed C–C bond formation is proposed to proceed via reaction of the enolate intermediate with the (*Re*)-face of l-P5C to give a *t*-CMP-CoA thioester, which is hydrolysed giving *t*-CMP (Fig. 1a)^13,14^.

The C-6 sidechain of natural C-1/C-6-functionalised carbapenems is likely introduced at a late stage during biosynthesis, making the engineered production of C-6 carbapenem analogues challenging^21,22^. Thus, there is interest in biocatalytic systems for stereocontrolled synthesis of carbapenem precursors functionalised at the C-1 and C-6-equivalent positions.

We describe the use of engineered CMPSs^9–12,23–25^, solely, and in tandem with an alkylmalonyl-CoA-forming enzyme, to catalyse the formation of 4,6-disubstituted-*t*-CMP stereoisomers, i.e. products with three contiguous chiral centres. Some of these products are converted by CarA giving bicyclic β-lactams. The results illustrate the biocatalytic versatility of crotonases and the utility of stereodifferentiating tandem enzyme reactions^26–29^, for synthesis of functionalised β-amino acids and bicyclic β-lactams.

## Results

### CMPS 4,6-disubstituted-*t*-CMP preparation

**Fig. 2:**
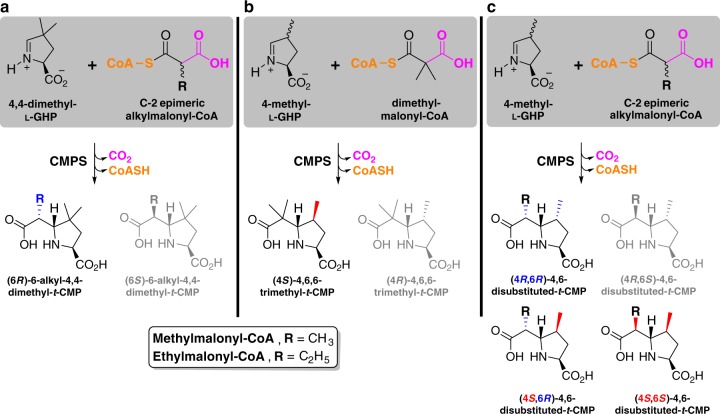
4,6-Trisubstituted-*N*-heterocycles by CMPS catalysis. Products observed (in black) on incubation of 4,4-dimethyl-l-GHP (**a**) and C-4 epimeric 4-methyl-l-GHP (**b**, **c**) with C-2 epimeric alkylmalonyl-CoA starting materials (in grey boxes). Structures of potential products not detected by LC–MS and NMR analyses are in grey

**Fig. 3:**
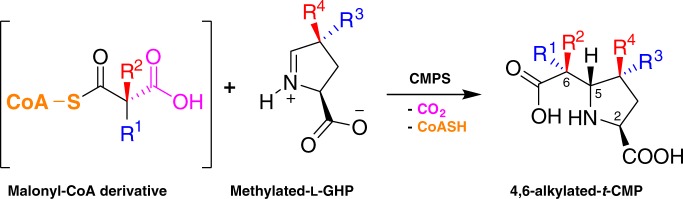
4,6-Disubstituted-*N*-heterocycles by (tandem) CMPS catalysis. Shown products are from incubation of C-4-alkylated-l-GHP and C-2 alkylmalonyl-CoA^*a*^, catalysed by the highest yielding/selective engineered CMPSs, or by use of MatB/CMPS or Ccr/CMPS. (*S*)-stereocentres are in red and (*R*)-stereocentres are in blue, throughout, for positions 4 and 6. See Table 1 for a list of substrates, diastereomeric ratios and yields

The task of producing C-1/C-6-functionalised carbapenams by CMPS catalysis is complicated by potential epimerisation of the precursors, i.e. at C-4 in l-GHP derivatives^10^ and at C-2 in malonyl-CoA derivatives^23^. At least for C-2 malonyl CoA derivatives, such epimerisation can be exploited in dynamic kinetic resolution^30,31^. We began by incubating 4,4-dimethyl-l-GHP^10^ (where C-4 epimerisation is irrelevant) and C-2 epimeric methylmalonyl-CoA with wild-type CarB and variants (Fig. 2). A new peak with the anticipated mass (*m*/*z* = 216 [M + H]^+^) was observed by LC–MS. Following scale-up, using CarB H229A, the highest yielding variant (as judged by NMR) (Supplementary Table 2), 1D/2D-NMR analyses led to assignment of the product as (6*R*)-4,4,6-trimethyl-*t*-CMP (Fig. 2a, Fig. 3, Table 1 entry 1, Supplementary Figs. 2 and 3). Incubation of 4,4-dimethyl-l-GHP, C-2 epimeric ethylmalonyl-CoA^17^ with CarB W79 variants (i.e. CarB W79F/A/Y/S) resulted in a single observed product, assigned as (6*R*)-6-ethyl-4,4-dimethyl-*t*-CMP (Fig. 2a, Table 1 entry 2, Supplementary Figs. 2, 4 and 5). Incubation of epimeric 4-methyl-l-GHP^10^ and dimethylmalonyl-CoA^13^ with wild-type CarB/variants resulted in (> 95% detected product) (4*S*)-4,6,6-trimethyl-*t*-CMP (Fig. 2b, Table 1 entry 3, Supplementary Figs. 2, 6 and 7), revealing potential for stereoselective formation of C-4/C-6-functionalised products. The tested ThnE/ThnE variants did not catalyse formation of any of the above 4,6-trisubstituted-*t*-CMP derivatives in detectable levels.

**Table 1:**
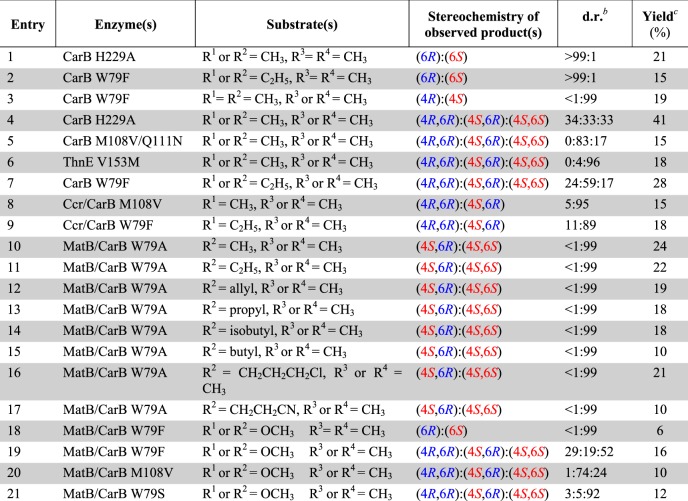
4,6-Disubstituted-*N*-heterocycles by (tandem) CMPS catalysis

See Fig. 3 for reaction scheme

^a^The C-2-alkylated-malonyl-CoA derivatives are Ccr or MatB products (see text and Fig. 6 for details)

^b^d.r.: diastereomeric ratio of epimers at C-4 and/or C-6 of *t*-CMP derivatives, determined by ^1^H NMR and/or LC–MS, under standard conditions. ^c^The % yield (isolated) was calculated^10,11,25^ following deprotection of amino acid aldehydes, incubation with enzyme(s), LC–MS purification and lyophilisation; products were quantified by NMR using [^2^H]4-trimethylsilylpropionate as a standard

**Fig. 4:**
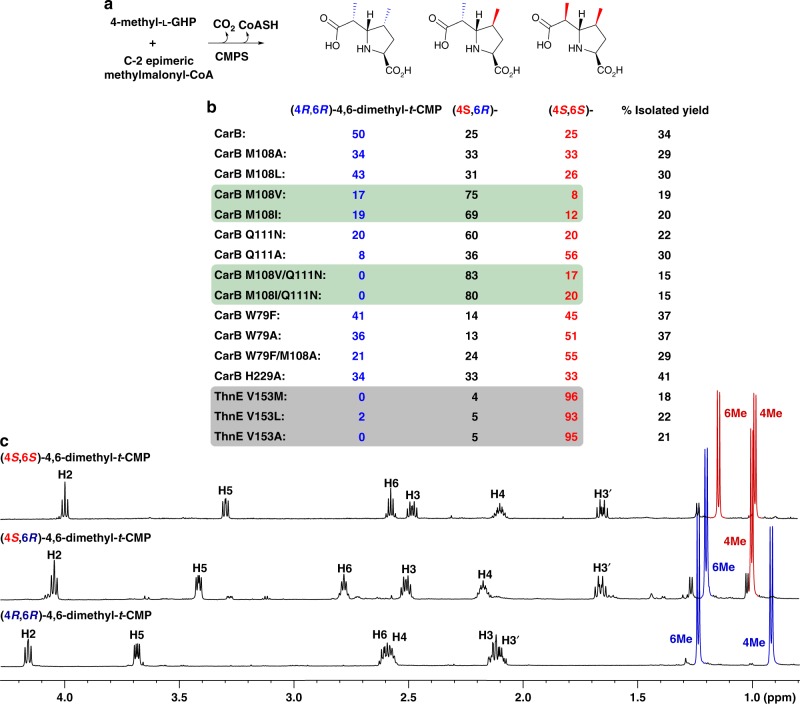
4,6-Dimethyl-*t*-CMP isomers produced by CMPS catalysis. The starting materials used are C-4 epimeric 4-methyl-l-GHP and C2-epimeric methylmalonyl-CoA. Variants manifesting relatively high stereoselectivity are shaded (green for CarB variants and grey for ThnE variants). Note that (4*S*,6*S*)-4,6-dimethyl-*t*-CMP can be produced from MatB/CMPS-catalysed reaction using methylmalonic acid and 4-methyl-l-GHP (Fig. 6b and Fig. 7). (4*S*,6*R*)-4,6-dimethyl-*t*-CMP is selectively produced from Ccr/CMPS-catalysed reaction of acryloyl-CoA and 4-methyl-l-GHP (Fig. 6A). **a** Substrates and observed products. **b** Observed stereoselectivities. **c** Exemplary ^1^H NMR spectra of the products

We then incubated epimeric 4-methyl-l-GHP and methylmalonyl-CoA with wild-type CarB; we observed two chromatographically distinct peaks with the anticipated mass (*m*/*z* = 202 [M + H]^+^). Scale-up and 1D/2D-NMR analyses revealed three stereoisomeric products: (4*R*,6*R*)-, (4*S*,6*R*)- and (4*S*,6*S*)-4,6-dimethyl-*t*-CMP, in an ~50:25:25 ratio (Fig. 2c, Fig. 4 and Supplementary Figs. 8-11). CarB variants (Supplementary  Table 2) catalysed formation of the same diastereoisomers of 4,6-dimethyl-*t*-CMP in varying yields and ratios, as confirmed by NMR analysis (Table 1 entries 4–7, Fig. 4 and Supplementary Fig. 11). No clear evidence for the formation of (4*R*,6*S*)-4,6-dimethyl-*t*-CMP was accrued. Notably, CarB variants with a β-branched residue at position-108 (i.e. CarB M108V/I) and CarB Q111N manifested selective production of (4*S*,6*R*)-4,6-dimethyl-*t*-CMP (Fig. 4). These observations guided us to test doubly substituted CMPSs, i.e. CarBM108V/Q111N and CarB M108I/Q111N, which manifest improved selectivity for production of the (4*S*,6*R*)-diastereomer (d.e. ≥ 0.6, Fig. 4). While wild-type ThnE catalysed formation of the 4,6-dimethyl-*t*-CMP isomers in relatively low yields, ThnE V153-based variants (ThnE V153M/L/A) catalysed formation of (4*S*,6*S*)-4,6-dimethyl-*t*-CMP, with d.e. ≥ 0.86, in ~18% isolated yield (small scale) (Table 1, entry 6, Fig. 4). Incubation of 4-methyl-l-GHP and (C-2 epimeric) ethylmalonyl-CoA with CarB W79-based variants (other variants gave lower yields) resulted in formation of three products with stereochemistries analogous to the methylmalonyl-CoA incubations: (4*R*,6*R*)-, (4*S*,6*R*)- and (4*S*,6*S*)-6-ethyl-4-methyl-*t*-CMP (Fig. 2c, Supplementary Figs. 12–15). The diastereomeric ratio with the CarB W79F-catalysed reaction (the highest yielding reaction, with 28% ‘isolated’ yield) was ~25 (4*R*,6*R*):59 (4*S*,6*R*):15 (4*S*,6*S*) (Table 1, entry 2). Incubation of 4,4-dimethyl-l-GHP and dimethylmalonyl-CoA did not manifest the anticipated *t*-CMP derivatives with any of the tested CMPSs (Supplementary Table 2).

**Fig. 5:**
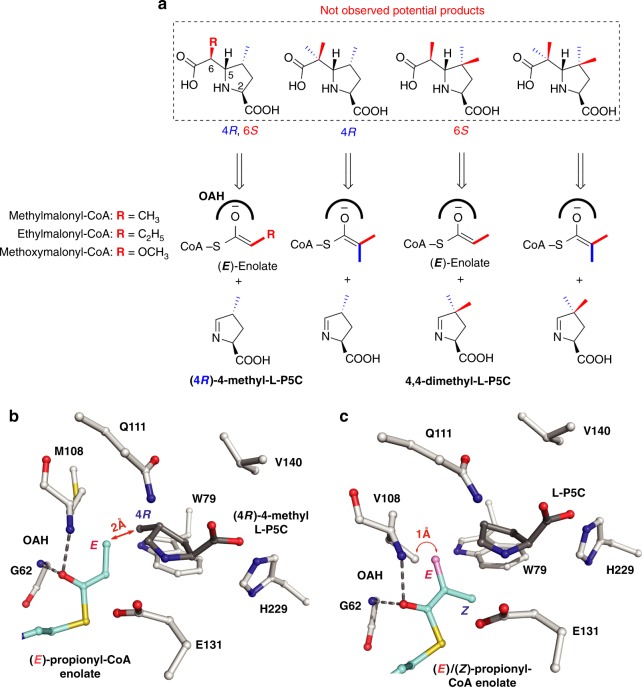
Mechanistic insights based on non-observed potential CMPS products. **a** Retro-catalytic analyses of non-observed potential products showing proposed requisite precursors, i.e. (*E*)/(*Z*)-enolate intermediates and the 4-substituted-l-P5C derivative. A common feature in potential production of the non-observed products is the presence of an (*E*)-enolate and (4*R*)-4-methyl-l-P5C. **b** View from a CarB structure with the modelled (*E*)-propionyl-CoA enolate, resulting from the decarboxylation of (2*R*)-methylmalonyl-CoA^25^, and (4 *R*)-4-methyl-l-P5C. The model implies proximity between the methyl group of the (*E*)-enolate and that of (4*R*)-4-methyl-l-P5C (~2 Å) suggesting a steric clash. The combination of an (*E*)-enolate and (4*R*)-4-methyl-l-P5C may thus be disfavoured, consistent with the lack of the potential products in **a**; **c** model of CarB M108V, with the (*E*)/(*Z*)-propionyl-CoA enolate (the methyl group of the (*E*)-enolate is in pink for distinction), and l-P5C. The model implies proximity between the methyl of the (*E*)-enolate and the β-methyl of valine-108 (~1 Å); the distance between the methyl of the (4*R*)-4-methyl-l-P5C (not shown for clarity) and the β-methyl of the valine residue is modelled at ~3 Å. Both these interactions thus may manifest a steric clash. The CarB M108V/I variants may thus preferentially catalyse formation of *t*-CMP derivatives with the (4*S*,6*R*)-stereochemistry (Fig. 4, entries in a green box)

These results provide further insights into CMPS selectivity. Consideration of the non-observed potential of CMPS products (Fig. 5a) in the light of crystallographic analyses implies a role for steric clashes in determining product outcomes. Thus, a clash between the methyl group of (4*R*)-4-methyl-l-P5C and the methyl group of the (*E*)-enolate (or a precursor of) may be responsible for their apparent lack of reaction with the tested CMPSs (Fig. 5b). The stereoselectivity of CarB variants with a β-branched residue (Val, Ile) at the OAH-forming residue-108 for formation of products with either (6*R*)- or (4*S*)-stereochemistry (Fig. 4, green-shaded boxes) can be rationalised on steric grounds, i.e. a clash between the methyl group of the (*E*)-enolate and the methyl group of (4*R*)-4-methyl-l-P5C (Fig. 5b), or with the β-methyl of the 108-valine/isoleucine residue is disfavoured (Fig. 5c). Thus, CarB variants with a β-branched 108-residue favour formation of the (4*S*,6*R*)-stereochemistry; this stereoselectivity is improved by Q111 substitution with Asn or Ala (Table 1, entry 5, Fig. 4, green-shaded boxes), possibly due to enhanced productive binding of the (4*S*)-4-methyl-l-P5C stereoisomer. On the other hand, we propose ThnE variants without a β-branched residue (Met, Leu and Ala) at residue-153 to favour the formation of (4*S*,6*S*)-stereochemistry products (Table 1, entry 6, Fig. 4, grey-shaded boxes), because of a preference to productively bind (4*S*)-4-methyl-l-P5C^10^ and hence form an (*E*)-enolate^25^.

In addition to mechanistic implications (Fig. 5), these results demonstrate the capacity of engineered CMPSs to catalyse formation of 4,6-alkyl-substituted *t*-CMP derivatives in high stereoselectivity. Although our ‘isolated’ yields are relatively low, given the micro-scale and non-optimised nature of the reactions, there is likely scope for improvement.

### Ccr/CMPS (4*S*,6*R*)-disubstituted-*t*-CMP preparation

**Fig. 6:**
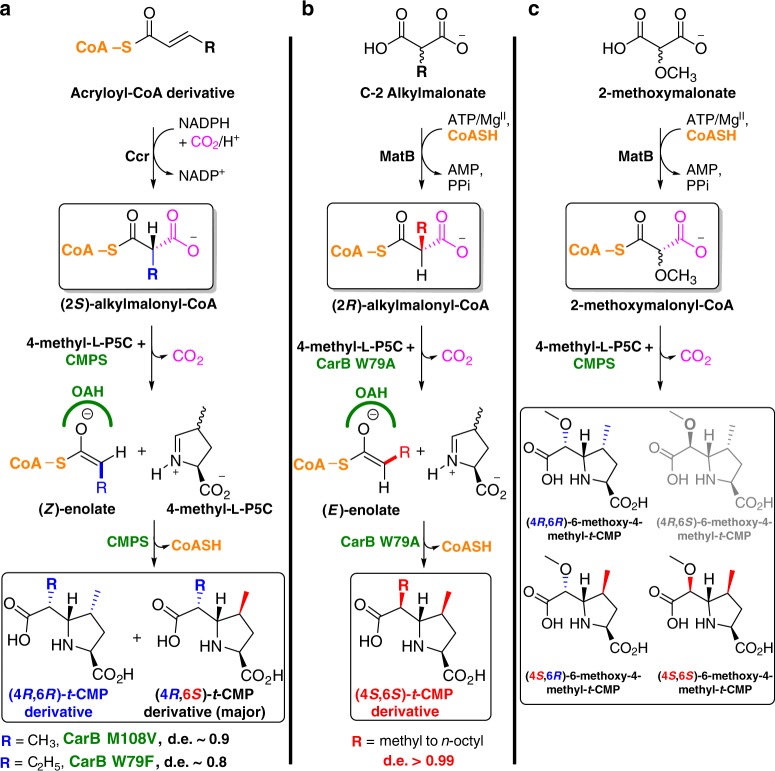
CMPS catalysis in tandem with an alkylmalonyl-CoA synthetase. a Incubation of 4-methyl-l-GHP and (2*S*)-alkylmalonyl-CoA (produced by Ccr)^32,33^, for selective formation of (4*S*,6*R*)-disubstituted-*t*-CMP derivatives. b Incubation of 4-methyl-l-GHP and (2*R*)-alkylmalonyl-CoA, produced by MatB catalysis from achiral alkylmalonic acids^24,34^, for selective formation of (4*S*,6*S*)-disubstituted-*t*-CMP derivatives. Note that CMPS-catalysed decarboxylation of (2*S*)-alkylmalonyl-CoA (**a**) is proposed to give the (*Z*)-enolate, while (2*R*)-alkylmalonyl-CoA (**b**) gives the (*E*)-enolate^25^. **c** Methoxymalonyl-CoA formation as catalysed by MatB and its one-pot reaction with 4-methyl-l-GHP, as catalysed by a CMPS to give the three shown stereoisomers. We propose that the nascent methoxymalonyl-CoA product of MatB catalysis is either epimeric or has the (2*R*)-stereochemistry, analogous to other MatB reactions (as in **c**)^24,34^, but undergoes relatively rapid epimerisation, consistent with the observed *t*-CMP products

Crotonyl-CoA carboxylase reductase (Ccr) catalyses formation of (2*S*)-methylmalonyl-CoA and (2*S*)-ethylmalonyl-CoA from acryloyl-CoA/CO_2_ or crotonyl-CoA/CO_2_, respectively (Fig. 6a)^32,33^. We have reported on the use of coupled Ccr/CMPS catalysis for production of (6*R*)-6-alkyl-*t*-CMP derivatives, likely via selective formation of the (*Z*)-enolate intermediates (Fig. 6a)^24,25^. We investigated use of the coupled system for formation of (4*S*,6*R*)-disubstituted-*t*-CMP derivatives, which are potential precursors for clinically used carbapenems.

One-pot incubation of acryloyl-CoA, sodium bicarbonate and 4-methyl-l-GHP with CarB M108V/I, Ccr and NADPH resulted in a mixture of (4*R*,6*R*)- and (4*S*,6*R*)-4,6-dimethyl-*t*-CMP in an ~5:95 ratio (Table 1, entry 8, Fig. 6a). Likewise, one-pot incubation of crotonyl-CoA and 4-methyl-l-GHP with Ccr and a CarB W79-based variant results in (4*R*,6*R*)- and (4*S*,6*R*)-4,6-dimethyl-*t*-CMP in an ~1:9 ratio, with the highest yielding variant being CarB W79F (Table 1, entry 9, Fig. 6a, Supplementary Fig. 16). By contrast, incubation of crotonyl-CoA and 4,4-dimethyl-l-GHP, catalysed by Ccr/CarB W79F (the highest yielding coupled system), manifested the (6*R*)-6-ethyl-4,4-dimethyl-*t*-CMP stereoisomer as the only observed product by LC–MS/NMR analyses (Supplementary Fig. 5). These results demonstrate that coupling with Ccr can enhance stereoselectivity in CMPS-catalysed formation of (4*S*,6*R*)-disubstituted-*t*-CMP derivatives.

### MatB/CMPS-catalysed (4*S*,6*S*)-disubstituted-*t*-CMP preparation

**Fig. 7:**
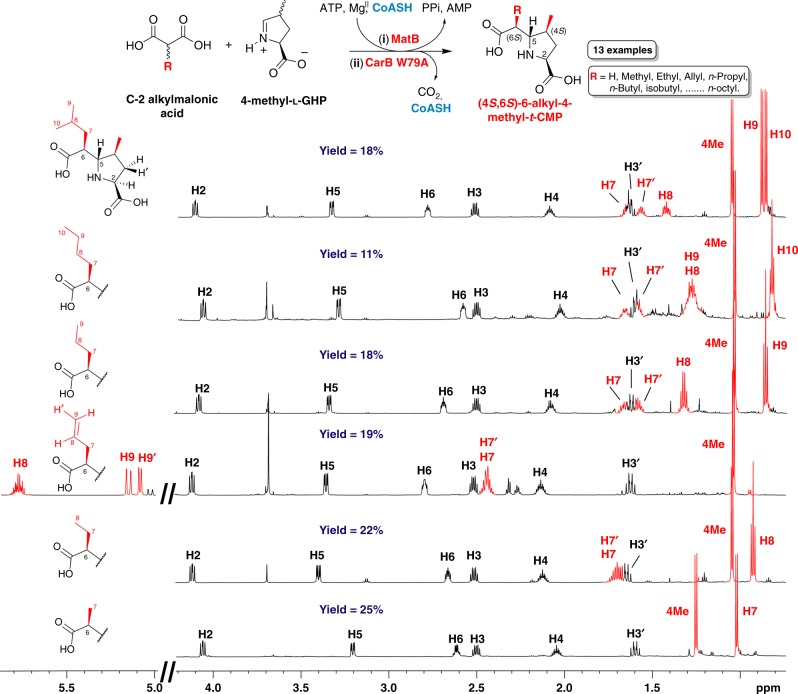
Stereoselectivity of tandem reactions catalysed by MatB and CarB W79A. The shown ^1^H-NMR spectra of the purified (4*S*,6*S*)-6-alkyl-4-methyl-*t*-CMP products reveal high (> 95%) stereocontrol at three centres (C-4, C5 and C-6) of products resulting from incubation of 4-methyl-l-GHP and C-2 precursors. For NMR characterisation of the isolated products, including assignment of stereochemistry, see Supplementary Methods and Supplementary Figs. 18–21, 23, 25 and 27–33

MatB catalyses formation of (2*R*)-alkylmalonyl-CoA derivatives, from achiral C-2 mono-alkylated malonic acid derivatives (Fig. 6b)^24,34^. We have reported on the use of malonyl-CoA synthetase (MatB)/CMPS coupling for stereoselective production of (6*S*)-6-alkyl-*t*-CMP derivatives (rather than the (6*R*)-epimer as with Ccr)^24,34^, likely via (*E*)-enolate intermediates derived from (2*R*)-alkylmalonyl-CoA derivatives^24^ (Fig. 6b). With a view to enhancing stereoselectivity of CMPS-catalysed formation of (4*S*,6*S*)-disubstituted-*t*-CMP derivatives, we investigated one-pot incubation of methylmalonic acid with 4-methyl-l-GHP in the presence of a CMPS (Supplementary Table 1), MatB, ATP and coenzyme A. A single product was observed that was assigned as (4*S*,6*S*)-4,6-dimethyl-*t*-CMP (Table 1, entry 10, Fig. 6b, Fig. 7).

We then investigated incubations with C-2-substituted-malonic acid derivatives ranging from ethylmalonic acid to derivatives with eight carbons. In some cases, reactions with 4-methyl-l-GHP, catalysed by CarB W79A, manifested single observed products, with the (4*S*,6*S*)-stereochemistry (shown by NMR) (Table 1, entries 11–15, Fig. 6b, Supplementary Figs. 16–21). Notably, the coupled MatB/CarBW79A system accepted substrates with polar groups, e.g. 2-(2-cyanoethyl)malonic acid, with a capacity for further modification (Table 1, entries 16–17, Supplementary Figs. 22–25). The capacity of CarB W79A to accept sterically demanding C-2-alkylated-malonyl-CoA derivatives, compared with other CMPSs (Supplementary Table 2), likely reflects its enlarged active site (Fig. 1b). In all tandem MatB/CarB W79A incubations, it appears that the (*E*)-geometry of the intermediate enolate, which results from the CarB W79A-catalysed decarboxylation of the (2*R*)-alkylmalonyl-CoA (the product of MatB catalysis), dictates the stereochemical outcome at C-4 of the product. By contrast, for Ccr/CMPS catalysis with 4-methyl-l-GHP, incubation of methylmalonic acid and 4,4-dimethyl-l-GHP in the presence of a CMPS (Supplementary Table 1) and MatB did not result in the formation of a CMP derivative (by LC–MS analysis). This result was anticipated since this reaction potentially involves a disfavoured interaction between the (*E*)-enolate intermediate (resulting from the decarboxylation of (2*S*)-methylmalonyl-CoA, produced by MatB catalysis) and (4 *R*)-4-methyl-l-P5C (Fig. 5b).

We then investigated the production of *t*-CMP derivatives with a C-6 heteroatom, using the capacity of MatB to form 2-methoxymalonyl-CoA from C-2 methoxymalonic acid (note that the product stereochemistry of this MatB product is unassigned)^34^. Unlike MatB/CMPS-catalysed incubation of 2-methylmalonic acid and 4,4-dimethyl-l-GHP, which did not manifest a detectable *t*-CMP product, incubation of methoxymalonic acid and 4,4-dimethyl-l-GHP with MatB/CarB W79F (the highest yielding coupled system), gave (6*R*)-4,4-dimethyl-6-methoxy-*t*-CMP as the only observed product (by LC–MS, NMR) (Table 1, entry 18, Supplementary Figs. 26 and 27). Incubation of methoxymalonic acid and 4-methyl-l-GHP with MatB/CarB W79F gave three stereoisomers (i.e. (4*R*,6*R*)-, (4*S*,6*R*)- and (4*S*,6*S*)-4,6-dimethyl-*t*-CMP), in an ∼30:20:50 ratio (Table 1, entry 19, Fig. 6c, Supplementary Figs. 26 and 28–33). The MatB/CarB M108V system exhibited bias towards formation of the (4*S*,6*R*)-stereoisomer (~0.5 d.e., Table 1, entry 20); however, the MatB/CarB W79S system exhibited bias towards the (4*S*,6*S*)-stereoisomer (~0.8 d.e., Table 1, entry 21 and Supplementary Fig. 28). These results imply that the 2-methoxymalonyl-CoA product of MatB catalysis is either epimeric at C-2 or undergoes epimerisation under assay conditions.

### Bicyclic β-lactam production

**Fig. 8:**
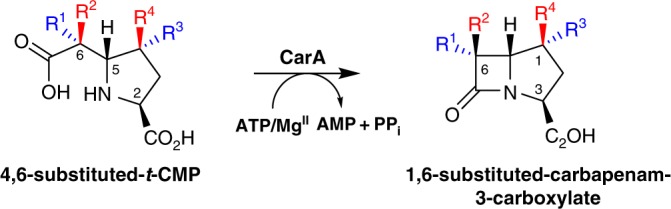
Conversion of 4,6-substituted-*t*-CMP derivatives into carbapenams. See Table 2 for substrates, products and conversions

To explore the utility of our methods for producing β-lactams, we investigated 4,6-disubstituted-t-CMP derivatives as CarA substrates. The three diastereomers of 4,6-dimethyl-*t*-CMP were converted by CarA into carbapenams, as confirmed by LC–MS and NMR analyses on crude reactions (Fig. 8, Table 2, entries 1–3, Supplementary Figs. 34–36). Turnover was nearly complete for (4*S*,6*R*)-4,6-dimethyl-*t*-CMP (≥ 90%), and ~65–70% for the other two stereoisomers. In the case of a reaction of an ∼1:1 mixture of the C-6 epimers (4*S*,6*R*)- and (4*S*,6*S*)-4,6-dimethyl-*t*-CMP, CarA exhibited a bias towards conversion of the (4*S*,6*R*)-stereoisomer (d.r. of products = 2:1, by LC–MS and NMR analyses on crude products, Supplementary Fig. 35).

**Table 2:**
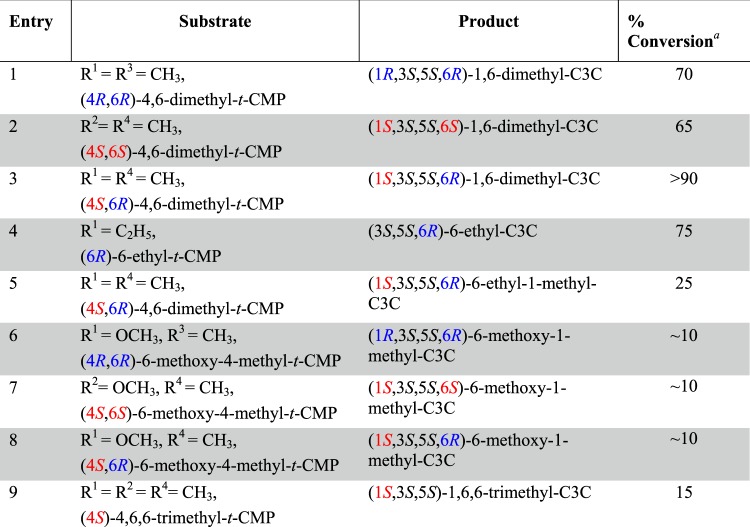
Conversion of 4,6-substituted-*t*-CMP derivatives into carbapenams

See Fig. 8 for reaction scheme

^a^% conversion by carbapenam synthetase catalysis, into 1,6-substituted-carbapenam-3-carboxylate (C3C) products was determined by LC–MS analysis measuring substrate conversion, in comparison with a control uncatalysed reaction. R^n^ = H, unless otherwise stated. (*S*)-stereocentres are in red and (*R*)-stereocentres are in blue, throughout, for positions 1 and 6

We observed that (6*R*)-6-ethyl-*t*-CMP,^25^ but not (6*S*)-6-ethyl-*t*-CMP^25^, is a good CarA substrate (75% conversion, Table 2, entry 4 and Supplementary Fig. 37). By contrast, out of the three CMPS-produced diastereomers of 4-methyl-6-ethyl-*t*-CMP, only (4*S*,6*R*)-4-methyl-6-ethyl-*t*-CMP was selectively converted by CarA (15% unoptimised small scale) (Table 2, entry 5, Supplementary Fig. 38), giving a carbapenam with the substitution pattern and stereochemistry of clinical carbapenems at C-1 and C-6. The three stereoisomers of 4-methyl-6-methoxy-*t*-CMP were relatively poor Car A substrates (~10% conversion was observed by LC–MS (Table 2, entries 6–8, Supplementary Fig. 34)).

Whilst under standard conditions, none of three 4,4,6-trisubstituted *t*-CMP derivatives prepared (i.e. (6*R*)-4,4,6-trimethyl-*t*-CMP, (6*R*)-6-ethyl-4,4-dimethyl-*t*-CMP and (6*R*)-4,4-dimethyl-6-methoxy-*t*-CMP) were Car A substrates, (4*S*)-4,6,6-trimethyl-*t*-CMP underwent ~15% conversion to the corresponding β-lactam (by LC–MS analysis) (Table 2, entry 9, Supplementary Fig. 34). The preference of CarA for substrates with the (4*S-*) and/or (6*R*)-stereochemistry is likely due to active site steric constraints (Supplementary Fig. 39), while the poor conversion of the 6-methoxy-*t*-CMP derivatives may additionally reflect introduction of a polar group.

The hydrolytic stability of unsubstituted carbapenams/carbapenems is reportedly low, to the extent that their isolation in the free form (rather than as ester derivatives) has not been readily possible^35–38^. We found that 1,6-disubstituted carbapenams are hydrolysed more slowly than their unsubstituted or monosubstituted analogues^10^, which undergo hydrolysis during LC–MS -guided purification/lyophilisation as evidenced by NMR. By contrast, the *t*_1/2_ of the (1*S*,3*S*,5*S*,6*S*)-1,6-dimethyl carbapenem was ~42 days by NMR (4 °C, sodium formate pH ∼7), revealing the stabilising effects of C-4/C-6 substitution.

## Discussion

The stereocontrolled synthesis of heterocycles, such as bicyclic β-lactams, with contiguous stereocentres is a challenge in development of natural products/natural product like drugs. Our results highlight the utility of engineered crotonases, and more generally enzyme-catalysed reactions proceeding via enolate intermediates, including when coupled with malonyl-CoA-forming enzymes, in addressing aspects of this challenge. We have described reactions with engineered CMPS enzymes with l-P5C giving CMP products substituted at C-6^23-25^. Introducing an epimeric methyl substituent at C-4 of l-P5C^10^, with a view to selectively preparing (4,6)-disubstituted-*t*-CMP derivatives with the (4*S*)-stereochemistry, which are potential precursors of 1β-methyl-carbapenams, increases the number of potential products to four stereoisomers (assuming conservation of (5*S*)-stereochemistry)^10,11,13,25^. The results (Fig. 3, Table 1) reveal the potential of engineered CMPS catalysis for stereocontrolled production of (4,6)-disubstituted-*t*-CMP derivatives, not only with the desired (4*S*,6*R*)-stereochemistry, as in most clinically used carbapenems, but for C-4/C-6-trisubstituted products (i.e. mono-alkylated at one of C-4 or C-6 and dialkylated at one of C-4 or C-6).

In the case of CMPS-catalysed reaction of C-2 epimeric alkylmalonyl-CoA with C-4 epimeric 4-methyl-l-P5C (Fig. 2c), of the four possible stereomeric products, one was not observed under standard conditions, i.e. the (4*R*,6*S*)-product. We propose that this is due to a steric clash involving the (*E*)-trisubstituted enolate and the methyl group of (4*R*)-methyl-l-P5C (Fig. 5b). This proposal implies scope for further engineering or expanding the scope of CMPS catalysis. Interestingly, substituting one of the oxyanion hole-forming residues (108_CarB_/153_ThnE_) has a major impact on C4/C6 stereocontrol; variants with a β-branched residue at this position favour formation of (4*S*,6*R*)-products, while ThnE variants lacking a β-branched residue favour formation of (4*S*,6*S*)-products (Fig. 5).

The results also reveal the capacity of the tandem MatB/CMPS system to enhance stereoselective formation of certain (4*S*,6*S*)-disubstituted-*t*-CMP derivatives, and to expand the range of accepted substrates. Thus, the stereoselectivity of CMPS-catalysed process can be enhanced by coupling an appropriately engineered CMPS with a malonyl CoA synthetase starting from a P5C derivative and an achiral C2-alkylated malonic acid derivative. Except for the case of 2-methoxymalonic acid, coupling MatB catalysis to that of engineered CMPSs enabled stereoselective formation of (4*S*,6*S*)-disubstituted-*t*-CMP derivatives, in some cases with high stereocontrol at C-4 and C-6. Similarly, coupling Ccr to engineered CMPSs enabled stereoselective formation of (4*S*,6*R*)-disubstituted-*t*-CMP derivatives, again with high stereocontrol at C-6 and > 75% stereocontrol at C-4. The range of substrates transformed by the MatB/CMPS pairs, including some with a heteroatom at C-6 is substantial. Some of these were converted by CarA into bicyclic β-lactams demonstrating the viability of the MatB–CMPS–CarA process for production of 1β-methyl-substituted carbapenams. Notably some of these products manifested improved hydrolytic stability compared with the unsubstituted 1β-carbapenams^35–38^. Thus, although challenges remain in developing the methods described here for the large-scale preparation of useful carbapenems, the results clearly demonstrate that engineering of biosynthesis enzymes has potential for the stereocontrolled production of functionalised bicyclic β-lactam derivatives.

## Methods

### Preparation of enzymes and variants reported

For details, see Supplementary Methods. All proteins were prepared and purified to > 95% by SDS-PAGE analysis. Mutagenesis of the plasmid-bearing *carB* or *thnE* genes was performed according to the QuikChange Site-Directed Mutagenesis Protocol (Stratagene). Supplementary Table 1 gives the oligonucleotide primers used for *carB* double-variants preparation. For a full list of the variants prepared and tested, see Supplementary Fig. 1 and Supplementary Table 2.

### Enzyme assays

Small- and large-scale assays of CMPSs, coupled MatB–CMPS, coupled Ccr–CMPS and CarA assays were performed and analysed as described in the Supplementary Methods and Supplementary Tables 3–5.

### Structural assignment of reported catalytic products

A combination of (high)-resolution MS and 2D-NMR analysis was employed, as fully detailed within the Supplementary Methods. Stereochemistries were assigned through combined analysis of ^3^*J*_HH_ coupling constants and 2D NOESY, assuming that the (*S*)-stereochemistry at C-2 is maintained during the acid-mediated deprotection of amino acid semialdehydes and product formation, as has been already confirmed^17^ (see Supplementary Figs. 2–38).

### Quantification of yields and diastereomeric ratio of the products of CMPS and CarA catalysis

Yields of different products of CMPS and CarA catalysis were calculated using a combination of LC–MS and ^1^H NMR spectroscopy, as detailed within the text (Fig. 3, Table 1 and as previously reported^10,11,25^).

## Supplementary information

Supplementary Information

## Data Availability

Data are available from the corresponding author on reasonable request.
