# Supplementary material for: Biocatalytic production of bicyclic β-lactams with three contiguous chiral centres using engineered crotonases
Source: Commun Chem. 2019 Jan 24;2(1):7. doi: 10.1038/s42004-018-0106-z (PMC6542682; doi:10.1038/s42004-018-0106-z)
Supplement: Supplementary file 1 — Supplementary Information [file 42004_2018_106_MOESM1_ESM.pdf]

Supplementary Methods

Materials and Methods

Unless otherwise stated, chemicals were from Alfa Aesar (Karlsruhe, Germany), Aldrich (Dorset, UK), Acros Chemicals (Loughborough, UK), Tokyo Chemical Industry UK Ltd, or Bachem (St. Helens Merseyside, UK), and used without further purification. HPLC grade solvents were from Rathburn (Walkerburn, UK) and used for chemical transformations, work-up and chromatography without further purification. Dried solvents were from Aldrich (Dorset, UK) or by filtration through columns containing activated aluminum oxide under argon. Silica gel 60 F254 analytical thin layer chromatography (TLC) plates were obtained from Merck (Darmstadt, Germany) and visualized under UV light, or with potassium permanganate stain. Chromatographic purifications were performed using prepacked SNAP columns on a Biotage SP1 Purification system (Uppsala, Sweden).

Water was purified by a Millipore Milli-Q system fitted with a 0.22 μm filter at the outlet. Solutions used in molecular biology and microbiology were prepared according to standard procedures[1] using Milli-Q water and were autoclaved or sterilised by filtration, as required. Isopropyl β-D-1-thioglactopyranoside (IPTG) was from Melford Laboratories Ltd., electrophoresis grade agarose was from Bioline, and acrylamide/bis-acrylamide stock solution was from Sigma. Bacto Tryptone, Yeast Extract and Bacto Agar for use in culture media were from Oxoid and Difco. Plasmids and enzymes were from Promega, Novagen, New England BioLabs, or Stratagene; unless otherwise stated. Oligonucleotide primers were from Sigma-Genosys. Molecular weight markers for SDS-PAGE (Prestained protein marker) were from Invitrogen. 1 kb DNA ladder for DNA electrophoresis was from New England Biolabs. Other materials were from QIAGEN and Roche, unless otherwise stated. FPLC columns and equipment, and small-scale gel filtration columns (PD-10) were from Amersham Biosciences. Spin concentrators for protein concentration were from Amicon.

NMR tubes (1 and 2 mm) were from Bruker. Deuterated solvents were from Sigma and Apollo Scientific Ltd. NMR Spectra were recorded using Bruker AVIII 700 MHz (with ^{1}H inverse cryoprobe) or Bruker DRX 500 MHz (with 1 mm inverse microprobe). Chemical shifts are given in ppm relative to the solvent peak. Coupling constants (J) are reported in Hz to the nearest 0.5 Hz. Prediction of dihedral angle (Φ) between vicinal hydrogen atoms was conducted using MestRe-J software employing the "HLA (Chemical groups)" equation.[2]

Low resolution ESI mass spectrometry was performed using a Micromass® Quattro Micro™ API mass spectrometer operating in positive or negative ionisation modes. High resolution (HR) ESI mass spectrometry was carried out using a Bruker μTOF spectrometer.

Protein Preparation

Ccr,[3] MatB,[4] CarA,[5] CarB, CarB variants,[6] ThnE and ThnE variants[6] were prepared and purified (>95% by SDS-PAGE analysis) as reported. For the double variants of CarB M108V/Q111N, CarB M108I/Q111N, the pET24a/*carB Q111N* construct was used as a template. The primers[6] used for site-directed mutagenesis were the same as those used for the preparation of CarB M108V and CarB M108I, respectively. For the production of the CarB W79S and CarB Q111A variants, the following primers were used, as reported,[6] using the pET24a/*carB* construct as a template:

CarB variant	Forward Primer (5' → 3')	Reverse Primer (3' → 5')
Q111A	gggtttcgcgttcgccctgatgtttgacc	ccatacccaaagcgcaagcgggactacaa
W79S	gacatcgaagagagcattgaccgcgtgatt	cgctcctgtagcttctctcgtaactggcgc

Supplementary Table 1: Primers used for CarB mutagenesis. Codons introducing the desired mutation are highlighted. Production of the desired mutant was verified by DNA sequencing.

CarB Variants	ThnE Variants
Wildtype CarB	Wildtype ThnE
CarB W79F	ThnE V153M
CarB W79A	ThnE V153L
CarB W79S	ThnE V153A
CarB W79F/M108A	
CarB M108A	
CarB M108L	
CarB M108V	
CarB M108I	
CarB M108V/Q111N	
CarB M108I/Q111N	
CarB Q111N	
CarB Q111A	
CarB H229A	

Supplementary Table 2: CMPS variants employed in this study.

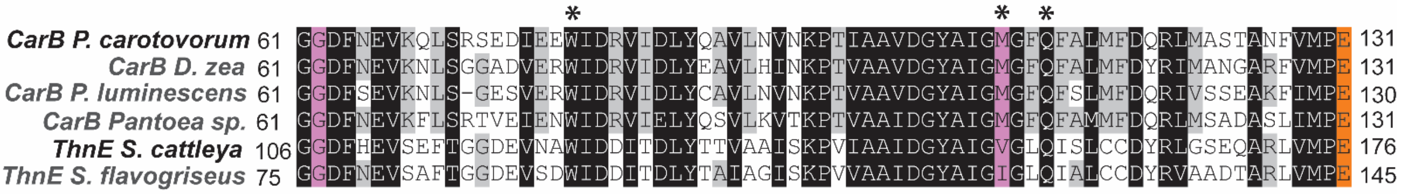

Supplementary Figure 1: A partial sequence alignment of known and putative CMPSs (the putative enzymes are in grey).[7] The proposed oxyanion hole forming residues are highlighted in pink and the catalytically important glutamate residue is in orange. Some of these residues (marked with asterisk) were targeted for engineering CarB and ThnE for control of multiple stereocentres in their t-CMP products of catalysis. The Figure was generated using Clustal W8 and Genedoc (http:// www.nrbsc.org/gfx/genedoc/).

III-A CMPS assays

Amino acid aldehydes were obtained by deprotection of the appropriate precursors as reported.[9] Analytical-scale wildtype and variant CMPS incubations were performed by sequential addition of the following reagents to a 0.5 mL Eppendorf tube (50 μL total volume):

	Volume	Final Concentration
600 mM Tris.HCl, pH 9.0	35 μL	420 mM
malonyl-CoA (or derivative thereof, 10 mM)	8 μL	1.60 mM
Amino acid semialdehyde in 10% formic acid (15 mM)	5 μL	1.50 mM
CMPS[a]	2/5 μL	~19-21 μM[a]

Supplementary Table 3 [a]The initial concentration of CarB/CarB variants was ~40 mg/mL (~0.48 mM) and that of ThnE/ThnE variants was ~20 mg/mL (~0.21 mM).

The incubation mixture was then kept at 37 °C for 30 min. To quench the reaction, an equal volume of methanol was added and the mixture cooled on ice for 10 min before centrifugation at 13,000 x g for 3 min. The supernatant was decanted and analysed by LC-MS. Control assays were performed in the same manner, but with substitution of 50 mM Tris-HCl pH 7.5 solution for the enzyme-containing solution. For quantification, the internal standard *p*-aminosalicylic acid was dissolved in the quenching solution of methanol (0.25 mM solution).

Small scale assay analyses

Products from small scale assays were analysed by LC-MS using either a Primesep 100 column (Sielc, 250 mm x 4.6 mm, 10 μm pore size, for relatively polar products) or an analytical C18 Column (150 mm x 4.6 mm, 5 μm pore size, for relatively nonpolar products) using a Waters 1525μ Binary HPLC Pump system equipped with a Waters 2777 Sample Manager coupled to a Micromass® Quattro micro™ API mass spectrometer (ESI+). The column was equilibrated at 1 mL/min with 5% eluent **B**. After 5 min, a gradient was run to 70% eluent **B** over 20 min. The column was washed with 100% eluent **B** for 5 min before the column was re-equilibrated at 5% eluent **B** for 10 min (overall run time is 40 min per assay).

Eluent **A**: 0.05% HCOOH in H_2O (v/v)

Eluent **B**: 0.05% HCOOH in MeCN (v/v)

For nonpolar *t*-CMP derivatives (those substituted at C-4 and C-6 with substituents totalling five carbons or more), a modified gradient was used as follows: The column was equilibrated at 1 mL/min with 5% eluent **B**. After 5 min, a gradient was run to 50% eluent **B** over 10 min. Then a gradient was run to 100% eluent **B** over 10 min The column was then washed with 100% eluent **B** for 5 min before the column was re-equilibrated at 5% eluent **B** for 10 min.

Large scale enzymatic product isolation and preparation for NMR characterization

Products for NMR analysis were produced by scale-up (10x) of assay conditions and incubation for 1 h at 37 °C, followed by quenching with MeOH (500 μL), centrifugation (13,000 x g) and freeze-drying of the supernatant. The resultant residue was re-suspended in 20 % aqueous methanol (300 μL) and purified using either or both of (**i**) a Waters Spherisorb column (250 mm x 10 mm, 5 μ, for relatively polar products) pre-equilibrated in 5% aqueous MeOH before a gradient was run to 10-25 % aqueous MeOH (according to the polarity of the product) with 0.1% aqueous formic acid; or (**ii**) a preparative C18 Column (250 mm x 22 mm, 15 μ, for relatively nonpolar products) pre-equilibrated in 5% aqueous MeCN with 0.1% aqueous formic acid before a gradient was run to 100 % MeCN with 0.1% aqueous formic acid over 40 min. In some

cases, the product was purified twice to obtain NMR spectra of sufficient quality. The 6-methoxy-*t*-CMP derivatives were initially purified by the Waters Spherisorb column then repurified employing the above mentioned preparative C18 Column.
Elution was monitored using a Micromass® Quattro Micro™ API mass spectrometer (equipped with a Waters 1525µ Binary HPLC Pump system coupled to a Waters 2777 Sample Manager). Fractions with *m/z* corresponding to anticipated products were collected (5-15 mL) and freeze-dried. The resultant residue was re-suspended in ^2H_2O (600 µL), transferred to an Eppendorf vial and freeze-dried. The final residue was re-suspended in ^2H_2O (13 µL for Bruker AVIII 700 MHz, or 6 µL for Bruker DRX 500 MHz (with microprobe)), transferred into a 1 mm NMR tube (Bruker) using a hand centrifuge, and analysed by NMR spectroscopy.

Quantification of yields and d.r. of the products of CMPS catalysis.
Yields of different products of CMPS catalysis were calculated using a combination of LC-MS and ^{1}H-NMR spectroscopy as follows: (**i**) The isolated yield obtained with a high yield producing CMPS variant for the product of interest was quantified (means of two experimental repeats) from protected L-GHP (or derivative thereof) according to the reported ^{1}H-NMR method[10] employing [^{2}H]$_4$-trimethylsilylpropionate as an external or internal standard; (**ii**) Using this ^{1}H-NMR quantified yield as a reference, the yields of other CMPSs were determined (means of two experimental repeats) by LC-MS assays using *p*-aminosalicylic acid as an internal standard. The d.r. of the products of (coupled) CMPS catalysis was determined by LC-MS and/or ^{1}H-NMR analyses.[6]

III-B Ccr/CMPS coupled assays

Analytical Ccr/CMPS incubations were performed by sequential addition of the following to a 0.5 mL Eppendorf tube:

	Volume	Final Concentration
Tris.HCl (600 mM, pH 9.0)	35 µL	150.00 mM
Amino acid semialdehyde in 10% formic acid (15 mM)	5 µL	0.54 mM
Tris.HCl (150 mM, pH 7.9)	67.5 µL	72.32 mM
NADPH (80 mM)	2.5 µL	1.43 mM
(*E*)-Crotonyl-CoA or acryloyl-CoA (10 mM)	10 µL	0.71 mM
$NaHCO_3$ (300 mM)	11 µL	23.57 mM
Ccr (6.25 µg/µl)	6 µL	5.61 µM
CMPS	3 µL	~10.3 µM

Supplementary Table 4

III-C MatB/CMPS coupled assays

Analytical MatB/CMPS incubations were performed by sequential addition of the following to a 0.5 mL Eppendorf tube:

	Volume	Final Concentration
Tris.HCl (600 mM, pH 9.0)	35 µL	200.00 mM
Amino acid semialdehyde in 10% formic acid (15 mM)	5 µL	0.71 mM
Tris.HCl (150 mM, pH 7.9)	42.5 µL	60.71 mM
Coenzyme A (10 mM)	5.5 µL	0.52 mM
ATP (100 mM in 50 mM Tris-HCL pH 8.0)	1.3 µL	1.24 mM
Malonic acid (or derivative thereof, 1M)	2.2 µL	20.95 mM
$MgCl_2$ (200 mM)	4.5 µL	8.57 mM
MatB (1.66 µM)	1 µL	0.02 µM
CMPS	3 µL	~13.8 µM

Supplementary Table 5

The assay mixtures were incubated at 37 °C for 2h then treated as described for CMPS assays. The C-2 alkylmalonic acid derivatives used in the coupled MatB/CMPS assays were obtained either as free acids or as di(m)ethyl esters. In the case of the (m)ethyl ester derivatives, they were subjected to alkaline hydrolysis. Thus, the (m)ethyl ester derivative (1 equivalent) and sodium/lithium hydroxide (2.2 equivalents) were dissolved in a round-bottom flask containing 10 ml water. The resulting solution was stirred at room temperature or at 40 °C for 2h then acidified with conc. hydrochloric acid (to pH 2-3) and extracted three times with 30 ml ethyl acetate. The combined organic layers were then dried using magnesium sulfate and after filtration a quantitative yield of the C-2 alkylmalonic acid was isolated after evaporation in *vacuo*.[4] The identity of the isolated products were confirmed by LC-MS (negative ionization mode) and NMR analyses.

III-D CarA assays

CarA analytical-scale incubations were performed as reported.[5] For larger scale incubations: the product of two preparative CMPS assays, after purification and freeze-drying, was incubated with the components of CarA assay (x3) for 1 h at 37 °C. The reaction mixture was then quenched with an equivalent volume of acetonitrile, incubated on ice for 10 min, centrifuged (13,000 x *g*). The supernatant was purified using a preparative C18 Column (250 mm x 22 mm, 15 μ) pre-equilibrated in 5% aqueous MeCN with 0.1% (v/v) aqueous formic acid before a gradient was run to 100 % MeCN with 0.1% (v/v) aqueous formic acid over 40 min. Elution was monitored using a Micromass® Quattro micro™ API mass spectrometer (ESI-). Fractions with *m*/*z* corresponding to the anticipated product were collected (~10 mL), 0.1 N sodium bicarbonate was added to pH 7.0, and then the neutralized fraction was freeze-dried. The resultant residue was re-suspended in ^2H_2O (500 μL), transferred to an Eppendorf vial and freeze-dried. The final residue was re-suspended in ^2H_2O (75 μL), transferred into a 2 mm NMR tube using a hand centrifuge, and analysed by NMR using a Bruker AVIII 700 equipped with a ^1H inverse cryoprobe.

Assignment of reported enzyme-catalysed products

NMR analyses were recorded at 298 K using a Bruker AVIII 700 MHz spectrometer equipped with a ^{1}H TCI-inverse cryoprobe optimised for ^{1}H observation (and running TOPSPIN 3 software), unless otherwise stated. Products were analysed by 2D COSY and NOESY (mixing time 800 ms) and stereochemistries were assigned through combined analysis of $^{3}J_{HH}$ coupling constants and NOEs. Selective 1D TOCSY experiments were conducted employing a DIPSI2 mixing scheme and zero-quantum suppression. Chemical shifts are reported in ppm relative to D_2O (δ_H 4.72); the deuterium signal was used as an internal lock signal and the HDO signal was reduced by presaturation where necessary. For quantification of the carboxymethylproline synthases products of catalysis, the trimethylsilane propionic acid sodium salt (TSP) was used as an external standard.

For spectroscopic identification of products of CMPSs catalysis, the following general considerations apply*:*

In all cases, the LC-MS analyses (positive or negative ion electrospray ionization) supported the formation of the product as shown by observation of the molecular ion and the ion arising from apparent decarboxylation of the product. The formation of a ring structure was assigned in part from the ^{1}H-NMR chemical shift of the bridgehead proton (H-5). All assignments assume that the (*S*)-stereochemistry at C-2 is maintained during the acid-mediated deprotection of amino acid semialdehydes and during product formation. Evidence has been reported confirming that this is the case for CarB- and ThnE-catalysed conversion of L-GHP to (2*S*,5*S*)-carboxymethylproline.[11,12] For all compounds reported, the assignment of the bridgehead carbon (C-5) as having (*S*)-stereochemistry was in part based on NOE data that showed no correlation between H-2 and the bridgehead proton. The NOE data between other protons within the ring system supported this assignment. For all (major) products of MatB/CMPS catalysis reported in this study, the assignment of C-4 of the 4-methyl-*t*-CMP derivatives as having (*R*) or (*S*)-stereochemistry was based on nOe data between H-5 and H-4 as well as H-5/H-2 and the methyl group at C-4. The assignment of C-6 of the 4,6-disubstituted *t*-CMP derivatives as having (*R*) or (*S*)-stereochemistry was based on a combination of coupling constant $J_{5,6}$ (*t*-CMP) and nOe data. In some cases, the assignment of stereochemistry at C-6 was further confirmed by conversion of the product into the corresponding β-lactam derivative by CarA catalysis.

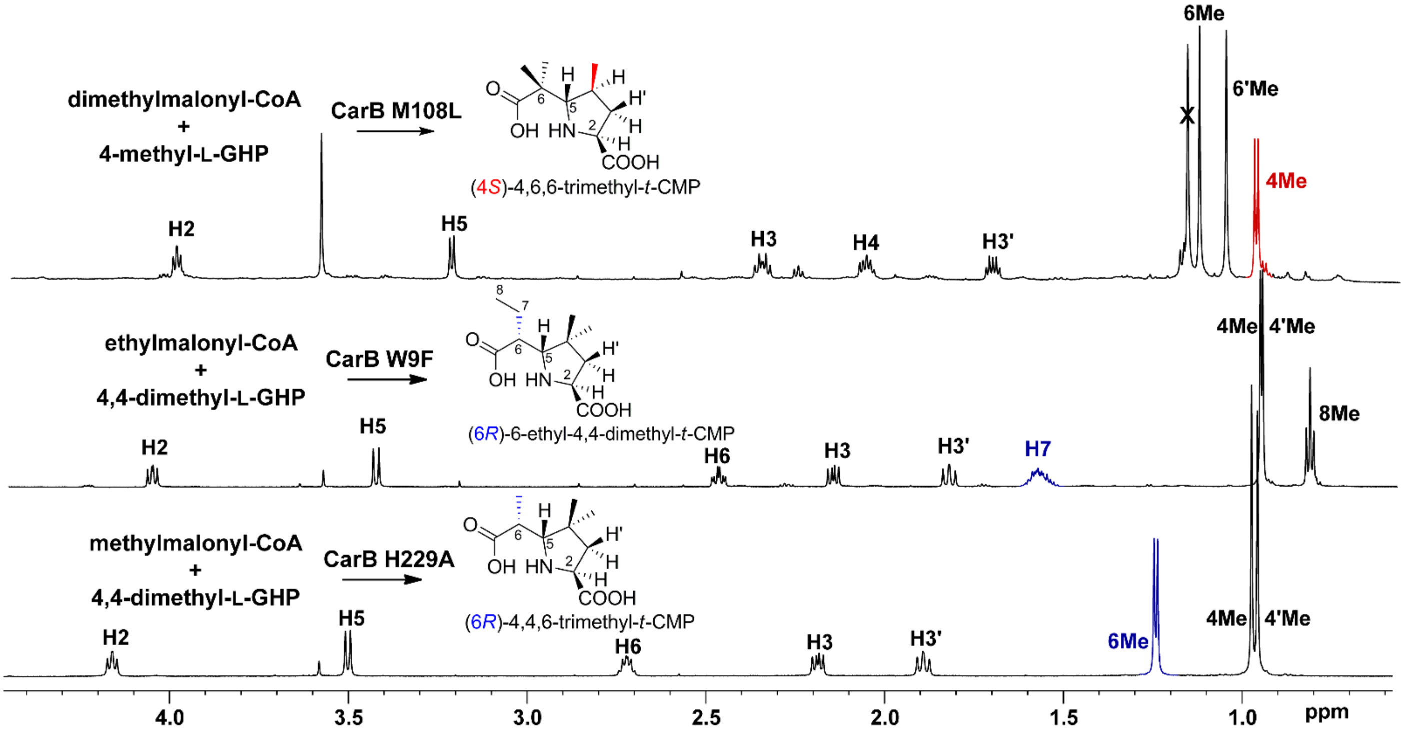

Supplementary Figure 2: ^{1}H NMR spectra for the purified C4 and C6 trisubstituted-*t*-CMP derivatives resulting from incubation of the stated C2-alkylmalonyl-CoA and C-4-methylated-L-GHP derivatives by the catalysis of the shown CMPSs.

Stereochemical assignment of 4,4,6-trimethyl-*t*-CMP (resulting from incubation of 4,4-dimethyl-L-GHP with the C-2 epimeric methylmalonyl-CoA in the presence of CMPSs)

The stereochemistry at C-6 was assigned as (*R*) on the basis of the following observations:

- A $J_{5,6}$ value of 9.8 Hz (predicted Φ ~ 170°) together with a weak nOe between H-5 and H-6, indicating an antiperiplanar relationship between the two protons.
- A strong nOe correlation between H-6 and one of the methyl groups at C-4 (4'Me), together with the absence of any nOe between the methyl group at C-6 and either of the methyl groups at C-4 (supported by 1D NOESY data involving the C-6 methyl group).

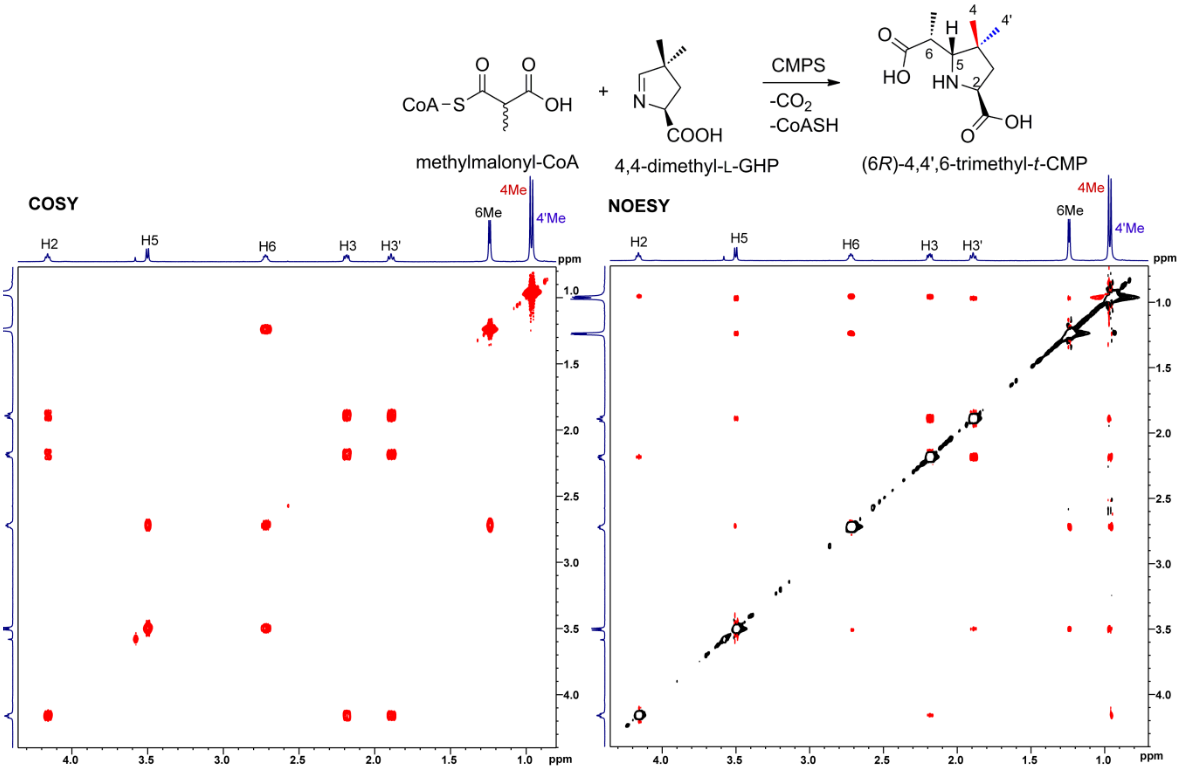

Supplementary Figure 3: ^{1}H-^{1}H COSY and NOESY spectra for (6*R*)-4,4,6-trimethyl-*t*-CMP produced from incubation of 4,4-dimethyl-L-GHP and C-2 epimeric methylmalonyl-CoA using CarB H229A catalysis.

Stereochemical assignment of 4,4-dimethyl-6-ethyl-*t*-CMP (resulting from incubation of 4,4-dimethyl-L-GHP with the C-2 epimeric ethylmalonyl-CoA in the presence of CMPSs)

The stereochemistry at C-6 was assigned as (*R*) on the basis of the following observations:

- A weak nOe between H-5 and H-6 together with the value of $J_{5,6}$ = 11.1 Hz, implying a predominately antiperiplanar relationship between the two protons.
- A strong nOe correlation between H-5 and H-7, absence of detected nOe between H-5 and 8-Me, together with the absence of any detected nOe between H-7/H-7' and the methyl groups at C-4.

It is notable that the tandem catalytic system comprising CarB W79F and Ccr using (*E*)-crotonyl-CoA as a co-substrate, under standard conditions, resulted in product with the same *m/z* and retention time as that resulting from the incubation of C-2 epimeric ethylmalonyl-CoA and 4,4-dimethyl-L-GHP (Supplementary Figure 4). On the other hand, coupling CarB W79F assay to that of MatB employing 2-ethylmalonic acid as a co-substrate, under standard conditions, did not result in the formation of a *t*-CMP derivative as supported by LC-MS analysis. These results are consistent with the lack of observation of formation of the (6*S*)-6-ethyl-4,4-dimethyl-*t*-CMP epimer by CMPS catalysis.

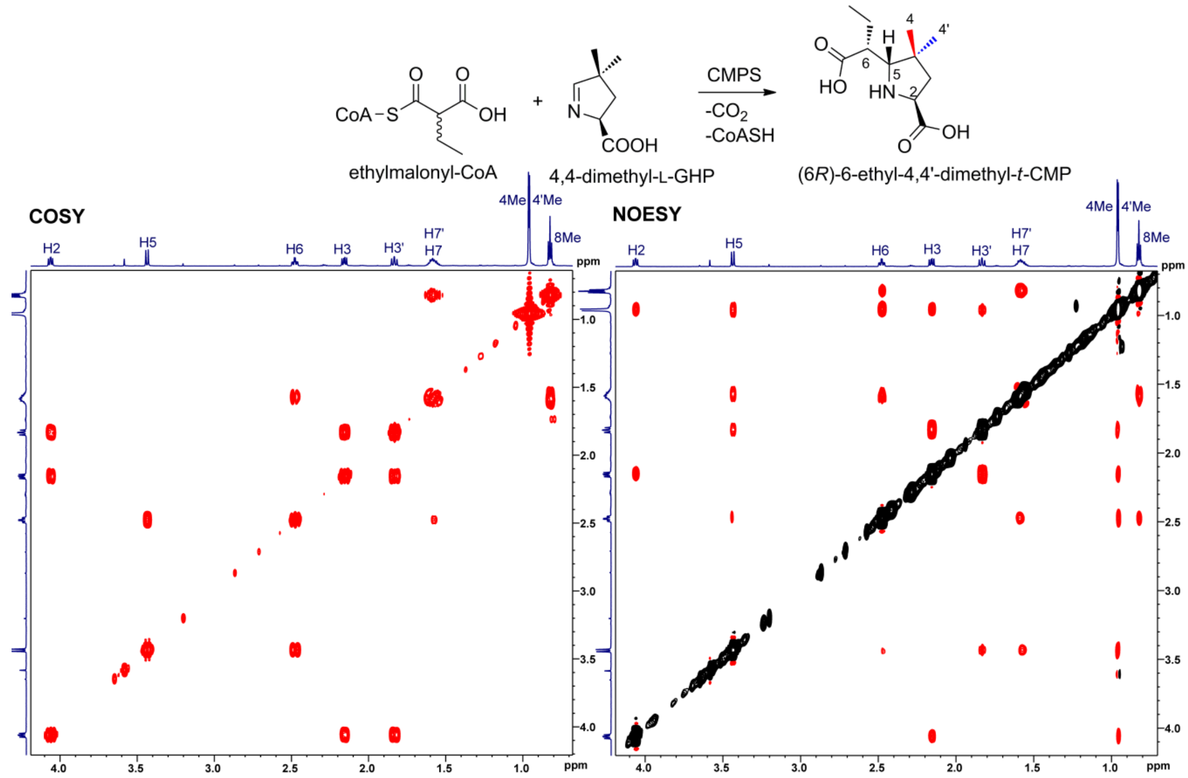

Supplementary Figure 4: ^{1}H-^{1}H COSY and NOESY spectra for (6*R*)-6-ethyl-4,4-dimethyl-*t*-CMP produced from incubation of 4,4-dimethyl-L-GHP and C-2 epimeric ethylmalonyl-CoA using CarB W79F catalysis.

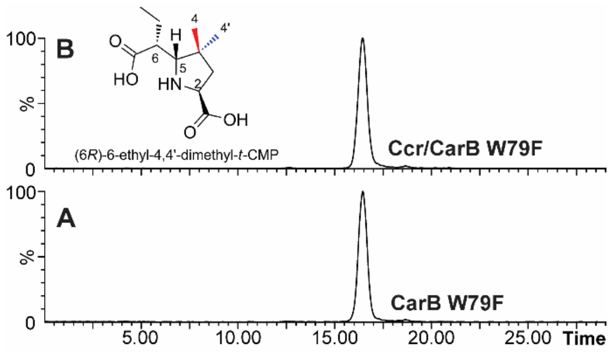

Supplementary Figure 5: LC-MS chromatograms for the (6*R*)-6-ethyl-4,4-dimethyl-*t*-CMP stereoisomer produced from: (A) incubation of C-2 epimeric ethylmalonyl-CoA and 4,4-dimethyl-L-GHP with CarB W79F; **(B)** incubation of (*E*)-crotonyl-CoA and 4,4-dimethyl-L-GHP in the presence of NADPH and sodium bicarbonate with coupled one pot Ccr/CarB W79F catalysis. It is noteworthy that incubation of 2-ethylmalonic acid 4,4-dimethyl-L-GHP, under standard conditions of MatB/CarB W79F coupled did not result in formation of a *t*-CMP derivative as supported by LC-MS analysis.

Stereochemical assignment of 4,6,6-trimethyl-*t*-CMP products (resulting from incubation of 4-methyl-L-GHP with dimethylmalonyl-CoA in the presence of CMPSs)

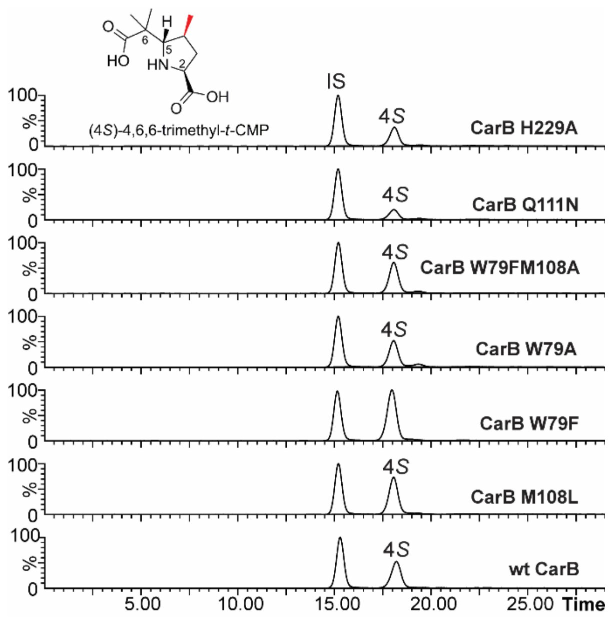

Supplementary Figure 6: LC-MS chromatograms displaying the formation of (4*S*)-4,6,6-trimethyl-*t*-CMP as a result of incubation of C-4 epimeric 4-methyl-L-GHP and dimethylmalonyl-CoA, under standard conditions, by the shown CMPSs. Note that CarB W79F is the highest yielding variant (19% isolated yield).

The stereochemistry at C-4 of the single detected product was assigned as (*S*) on the basis of the observation of a strong nOe between H-5 and the methyl group at C-4, together with the observation of a weak nOe between H-5 and H-4 (Supplementary Figure 6).

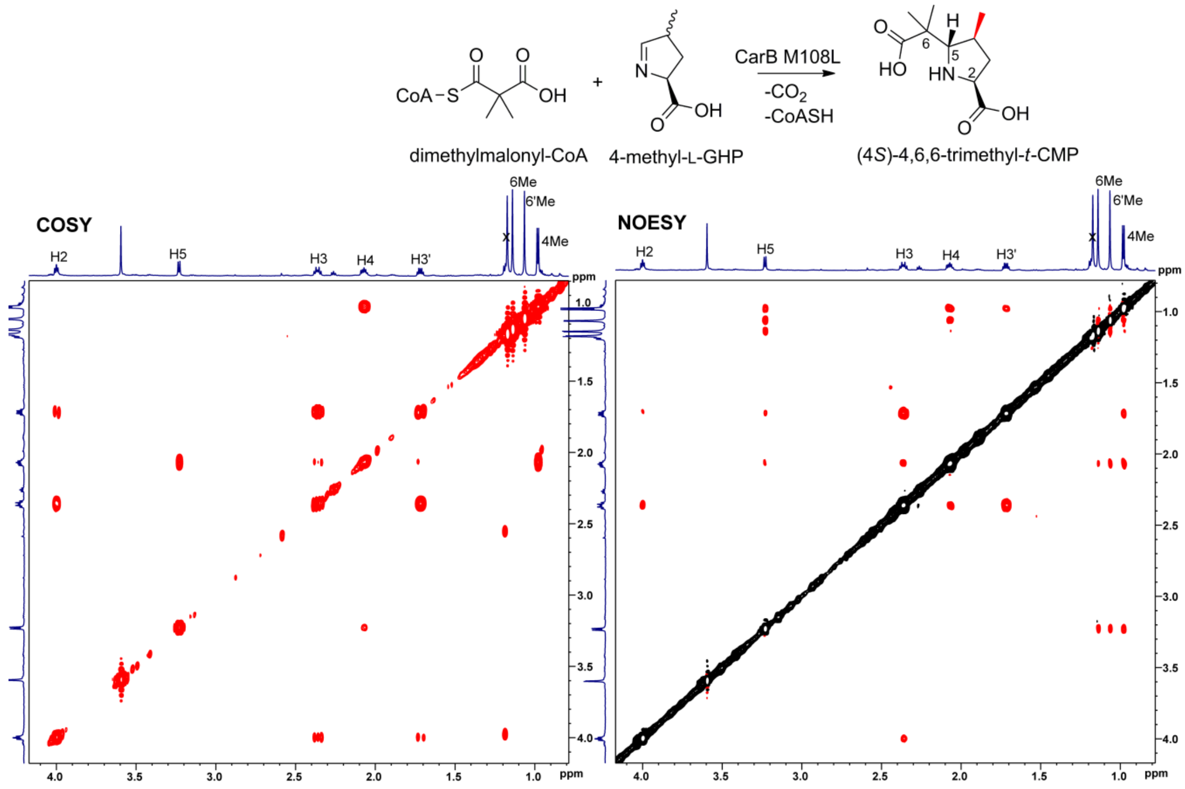

Supplementary Figure 7: ^{1}H-^{1}H COSY and NOESY spectra for (4*S*)-4,6,6-trimethyl-*t*-CMP produced from incubation of the C-4 epimeric 4-methyl-L-GHP and dimethylmalonyl-CoA by CarB M108L catalysis.

Stereochemical assignment of 4,6-dimethyl-*t*-CMP stereoisomers (resulting from incubation of C-4 epimeric 4-methyl-L-GHP and C-2 epimeric methylmalonyl-CoA in the presence of CMPSs)

Note: HRMS (ESI-TOF) *m*/*z*: $[M + H]^+$ Calcd for $C_9H_{16}O_4N$ 202.1074; Found 202.1076.

Three products were detected and labelled **A**, **B** and **C**, according to their order of elution. For compound **A**, the stereochemistry at C-4 was assigned as (*R*) based on the observation of a strong nOe between H-2 and the C-4 methyl group, together with the absence of an nOe between the methyl group at C-4 and H-5. The stereochemistry at C-6 was assigned as (*R*) on the basis of the following observations:

- A $J_{5,6}$ value of 10 Hz (predicted Φ ~ 170º) in addition to a weak nOe between H-5 and H-6 indicating an antiperiplanar arrangement for these two protons.
- A strong nOe correlation observed between H-6 and C-4 methyl group, as well as the absence of any nOe between the C-6 methyl group to H-4 or the methyl group at C-4.
- The assignment was confirmed by the CarA-catalysed conversion of the product into the corresponding β-lactam, which displayed a $J_{5,6}$ = 5.6 Hz (typical for β-lactams with H5 and H6 in a *cis*-relationship), Supplementary Figure 33.[13]

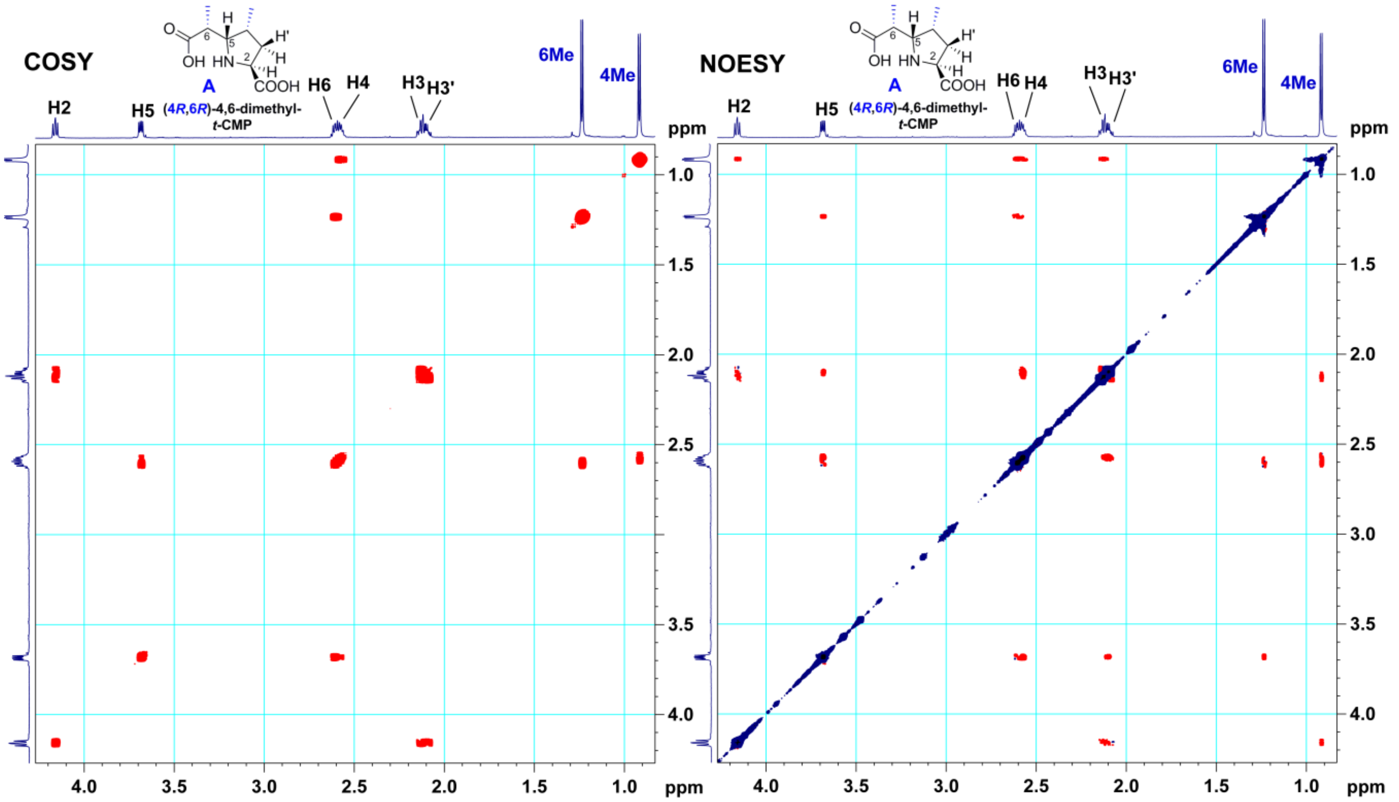

Supplementary Figure 8: ^{1}H-^{1}H COSY and NOESY spectra for purified (4*R*,6*R*)-4,6-dimethyl-*t*-CMP (A) produced from C-4 epimeric 4-methyl-L-GHP and C-2 epimeric methylmalonyl-CoA by CarB catalysis.

For compound **B**; the stereochemistry at C-4 was assigned as (*S*) based on the observation of a strong nOe between H-5 and the C-4 methyl group, together with the observation of a weak nOe between H-5 and H-4. The stereochemistry at C-6 was assigned as (*R*) based on the following observations:

- A $J_{5,6}$ value of 4.8 Hz (predicted Φ ~ 50º) together with a strong nOe between H-5 and H-6 indicating a predominately synclinal relationship between these two protons.
- Strong nOe correlations between H-6 and both H-4 and the methyl group at C-4, and the observation of (medium) nOe correlations between the C-6 methyl group to both H-4 and the C-4 methyl group.
- The assignment was confirmed by the CarA-catalysed conversion of the product into the corresponding β-lactam (Fig. 4), which displayed a $J_{5,6}$ = 5.0 Hz (typical for β-lactams with H5 and H6 in a *cis*-relationship).[13]

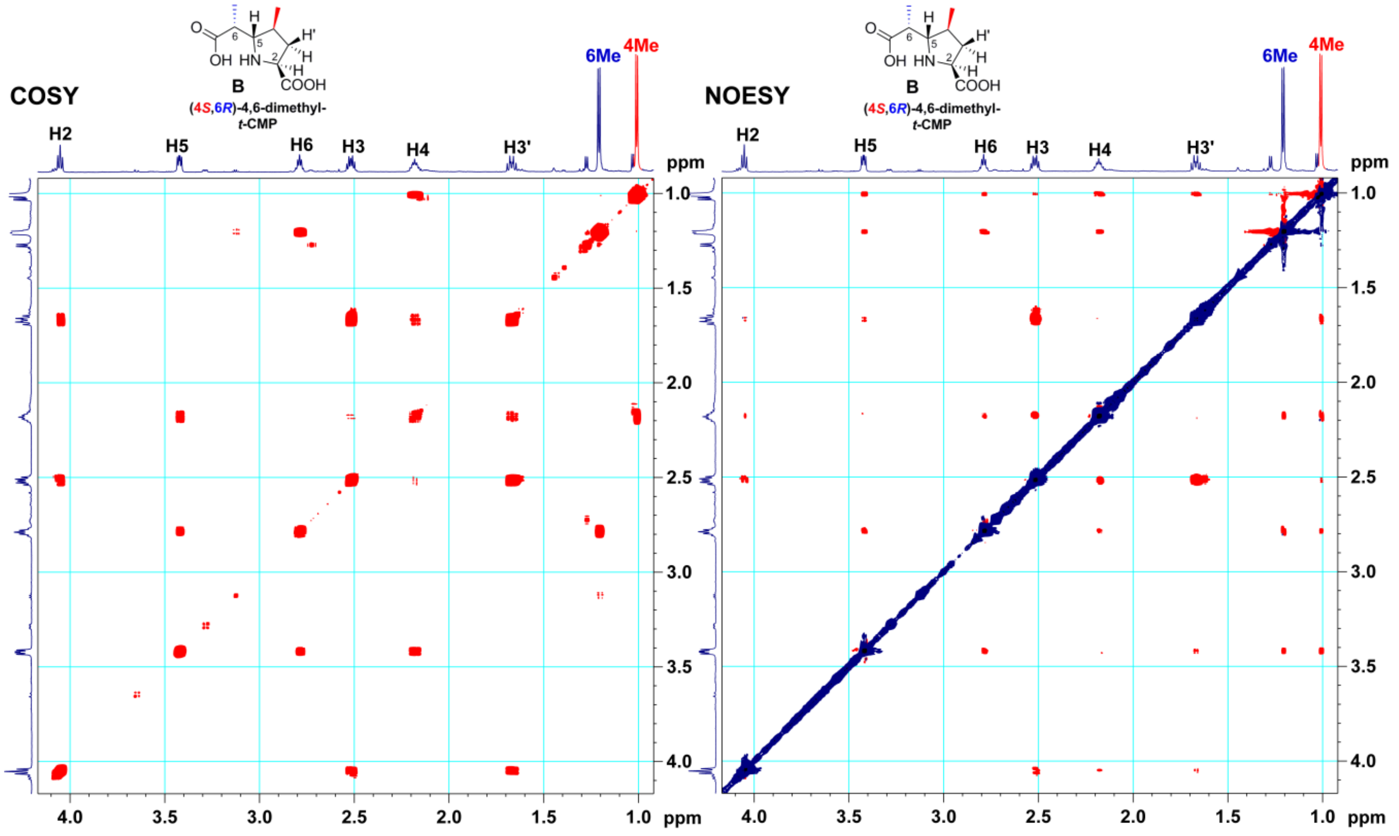

Supplementary Figure 9: ^{1}H-^{1}H COSY and NOESY spectra for purified (4*S*,6*R*)-4,6-dimethyl-*t*-CMP (B) produced from C-4 epimeric 4-methyl-L-GHP and C-2 epimeric methylmalonyl-CoA by CarB catalysis.

For compound **C**, the stereochemistry at C-4 was assigned as (*S*) based on the observation of a strong nOe between H-5 and the C-4 methyl group, together with the observation of a weak nOe between H-5 and H-4. The stereochemistry at C-6 was assigned as (*S*) based on the following observations:

- A $J_{5,6}$ value of 4.8 Hz (predicted Φ ~ 50º), together with a strong nOe between H-5 and H-6 indicating a predominately synclinal relationship between these two protons.
- The lack of observation of an nOe correlation between H-6 and the methyl group at C-4, together with the presence of weak nOe correlations between H-6 and H-4, the C-6 methyl group and H-4 and between the C-6 methyl group and the C-4 methyl group.
- The assignment was confirmed by the CarA-catalysed conversion of the product into the corresponding β-lactam, which displayed a $J_{5,6}$ = 1.2 Hz (typical for β-lactams with H5 and H6 in a *trans*-relationship), Fig. 4.[13]

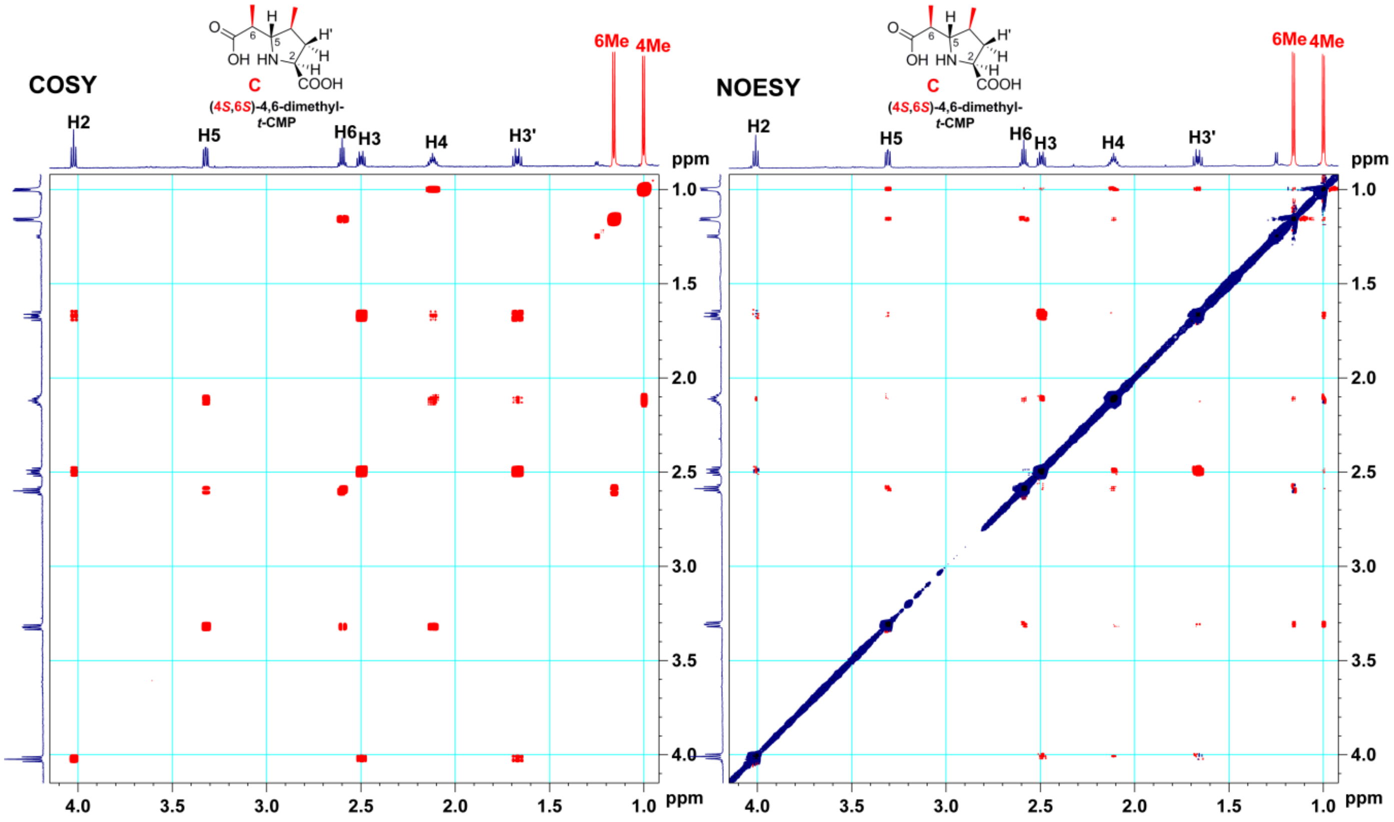

Supplementary Figure 10: ^{1}H-^{1}H COSY and NOESY spectra for purified (4*S*,6*S*)-4,6-dimethyl-*t*-CMP (C) produced from C-4 epimeric 4-methyl-L-GHP and C-2 epimeric methylmalonyl-CoA by CarB catalysis.

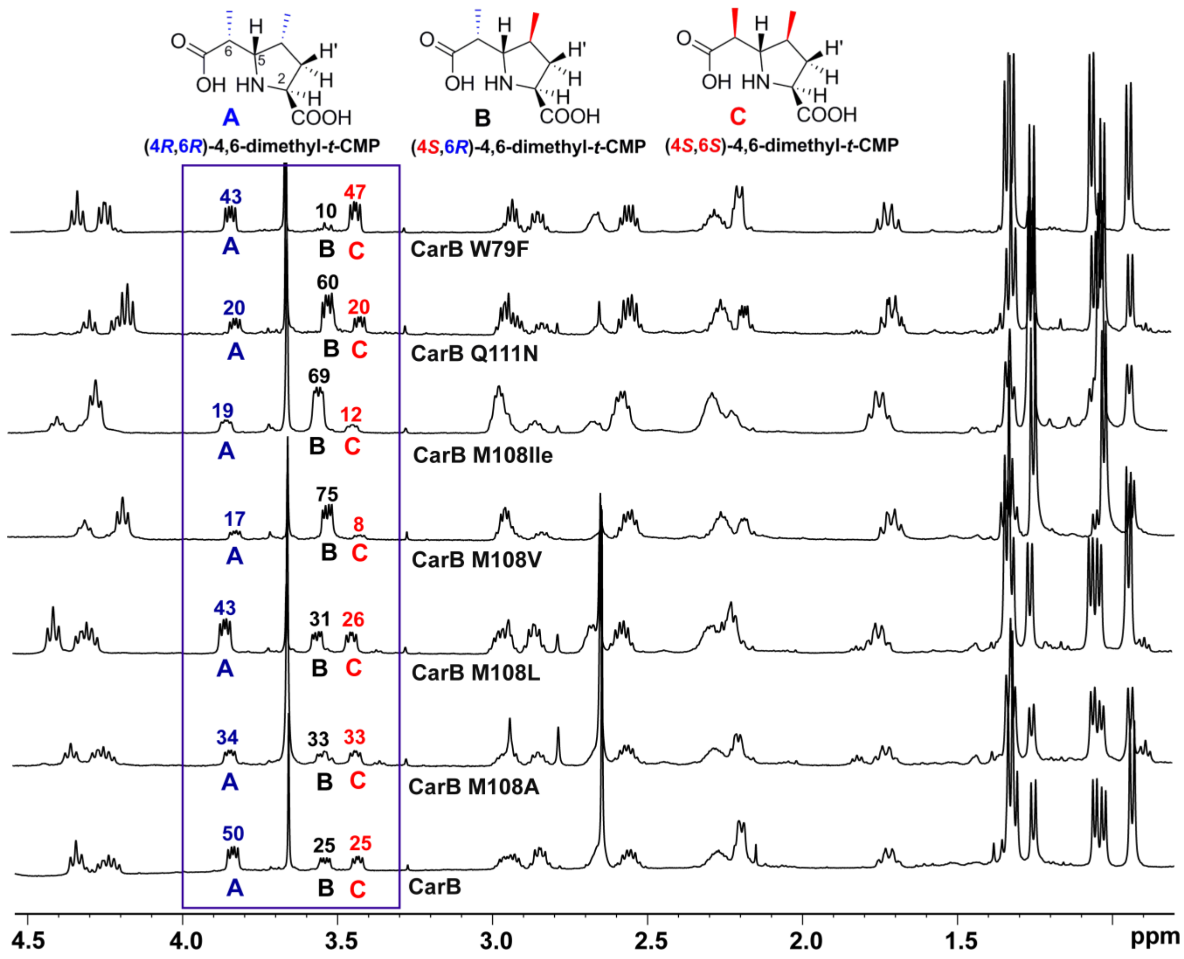

Supplementary Figure 11: Determination of the d.r. of the three detected stereoisomers of 4,6-dimethyl-*t*-CMP produced by incubation of C-2 epimeric methylmalonyl-CoA and C-4 epimeric 4-methyl-L-GHP with the shown CMPSs.

Stereochemical assignment of 4-methyl-6-ethyl-*t*-CMP stereoisomers (resulting from incubation of 4-methyl-L-GHP with the C-2 epimeric ethylmalonyl-CoA in the presence of CMPSs)

Three products were detected and labelled **A**, **B** and **C**, according to their order of elution.

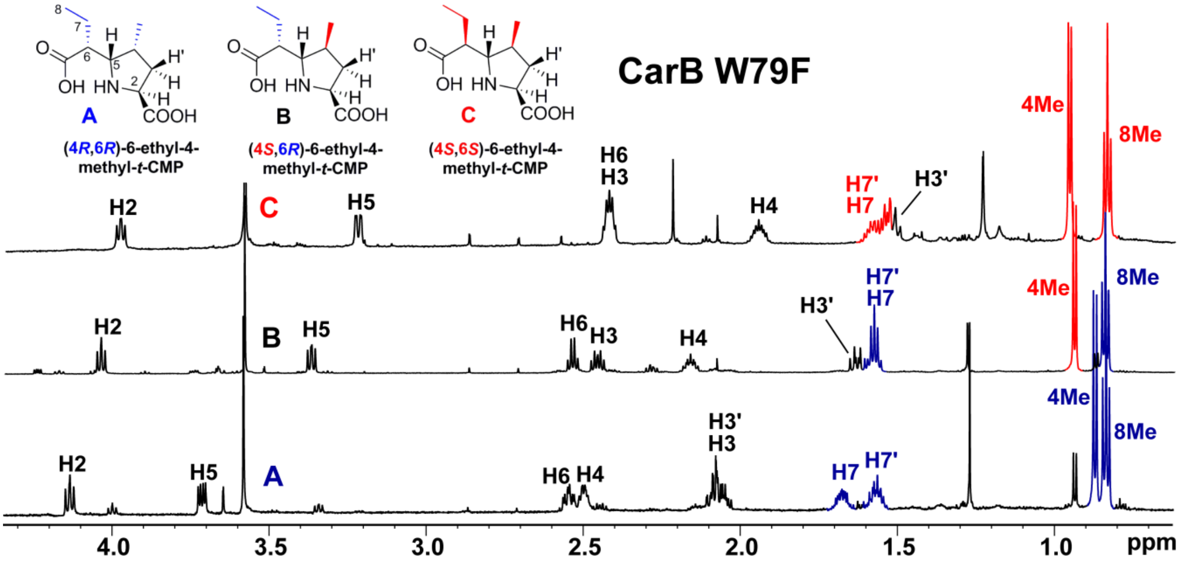

Supplementary Figure 12: ^{1}H-NMR spectra for the three detected C-4, C-6 stereoisomers of 6-ethyl-4-methyl-*t*-CMP produced by incubation of C-2 epimeric ethylmalonyl-CoA and C-4 epimeric 4-methyl-L-GHP with CarB W79F.

For compound **A**, the stereochemistry at C-4 was assigned as (*R*) based on the observation of a strong nOe between H-2 and the C-4 methyl group, together with the absence of any nOe between the methyl group at C-4 and H-5. The stereochemistry at C-6 was assigned as (*R*) on the basis of the following observations:

- The lack of nOe between H-5 and H-6, together with the value of $J_{5,6}$~ 11 Hz implying a predominately *anti* relationship between the two protons.
- A strong nOe between H-5 and H-7 as well as 8-Me together with the absence of any detectable nOe between H-4 and H-7 nor 4Me to H-7.

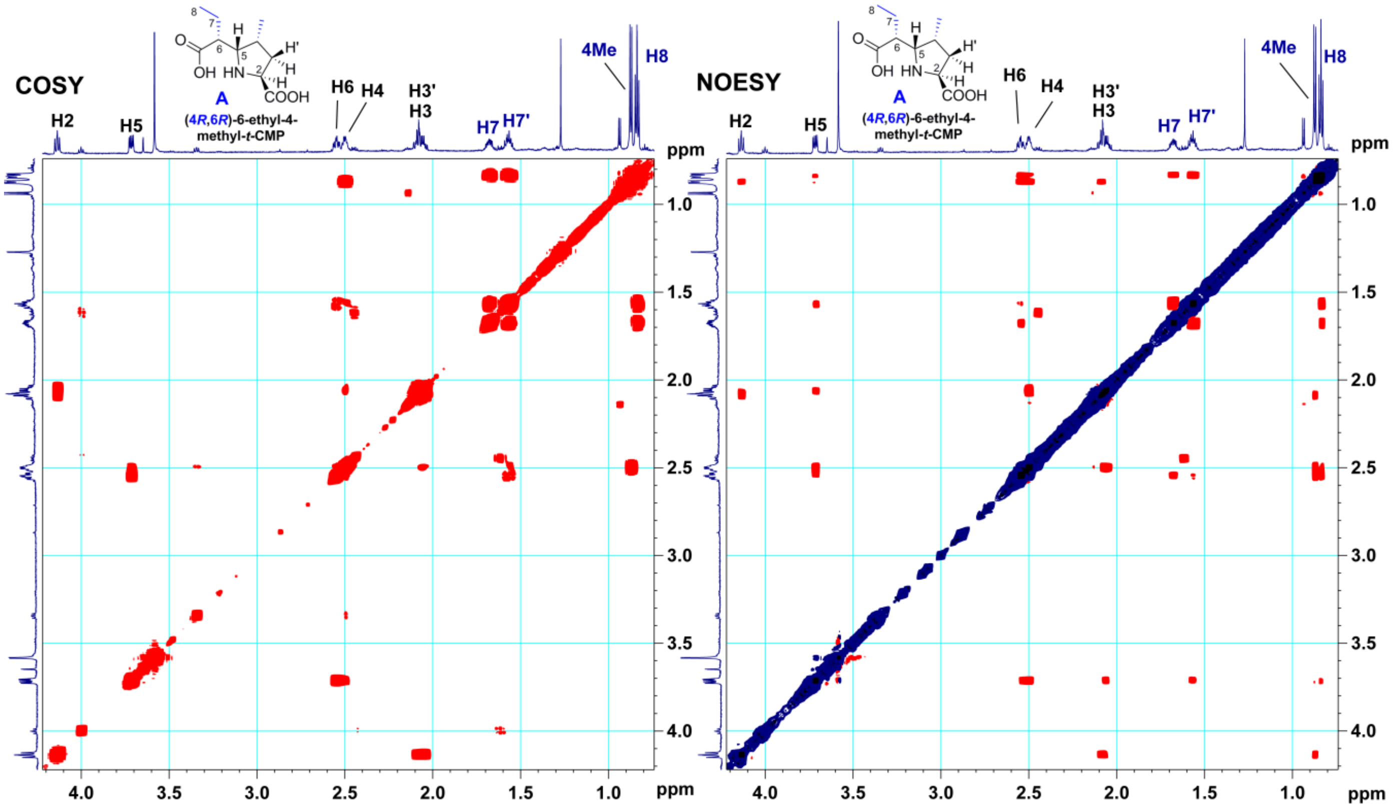

Supplementary Figure 13: ^{1}H-^{1}H COSY and NOESY spectra for (4*R*,6*R*)-6-ethyl-4-methyl-*t*-CMP (A) produced from C-4 epimeric 4-methyl-L-GHP and C-2 epimeric ethylmalonyl-CoA by CarB W79F catalysis.

For compound **B**, the stereochemistry at C-4 was assigned as (*S*) based on the observation of a strong nOe correlation between H-5 and the C-4 methyl group coupled to the nOe correlation between H-4 and H-2. The stereochemistry at C-6 was assigned as (*R*) on the basis of the following observations:

- A strong nOe between H-5 and H-6, together with the value of $J_{5,6}$~ 7.3 Hz implying a predominately *gauche* relationship between these two protons.
- A strong nOe between H-5 and H-7/H-7', as well as a weak nOe between H-5 and the protons at C-8.
- Weak nOe correlations between H-4 and H-7/H-7'.
- The assignment was confirmed by the CarA-catalysed conversion of the product into the corresponding β-lactam (Supplementary Figure 35), which displayed a $J_{5,6}$ = 4.9 Hz (typical for β-lactams with H5 and H6 in a *cis*-relationship).[13]

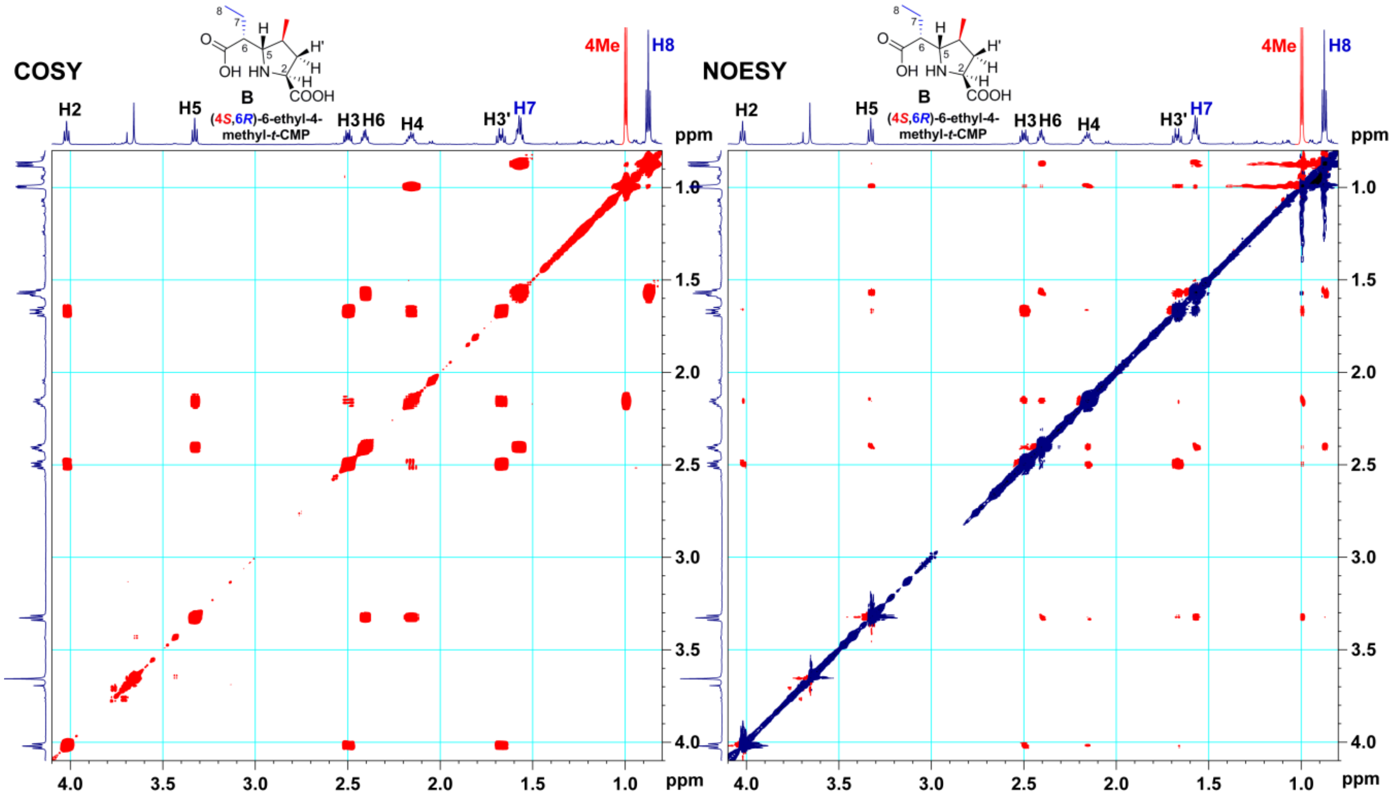

Supplementary Figure 14: ^{1}H-^{1}H COSY and NOESY spectra for (4*S*,6*R*)-6-ethyl-4-methyl-*t*-CMP (B) produced by incubation of C-4 epimeric 4-methyl-L-GHP and C-2 epimeric ethylmalonyl-CoA with CarB W79F.

For compound **C**, the stereochemistry at C-4 was assigned as (*S*) based on the observation of a strong nOe correlation between the methyl group at C-4 and H-5.

The stereochemistry at C-6 was assigned as (*S*) based on the following observations:

- A $J_{5,6}$ value of 4.2 Hz together with a strong nOe between H-5 and H-6 indicating a predominately *gauche* relationship between these two protons.
- The medium nOe correlation between H-5 and H-7/H-7'.
- The lack of observation of an nOe correlation between H-6 and H-4, together with the presence of a medium nOe correlation between H-6 and the methyl group at C-4.

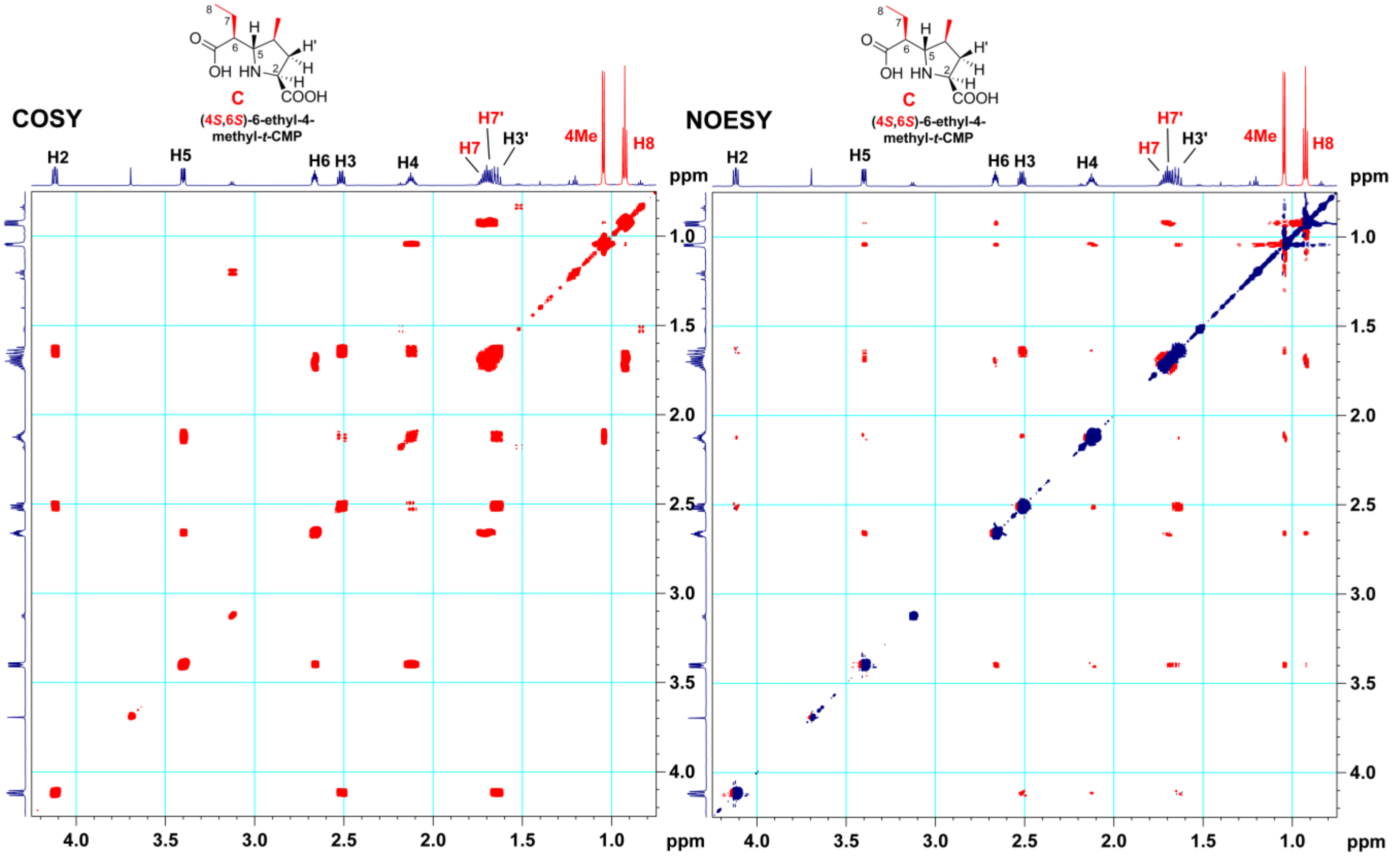

Supplementary Figure 15: ^{1}H-^{1}H COSY and NOESY spectra for (4*S*,6*S*)-6-ethyl-4-methyl-*t*-CMP (C) produced by incubation of C-4 epimeric 4-methyl-L-GHP and C-2 epimeric ethylmalonyl-CoA with CarB W79F.

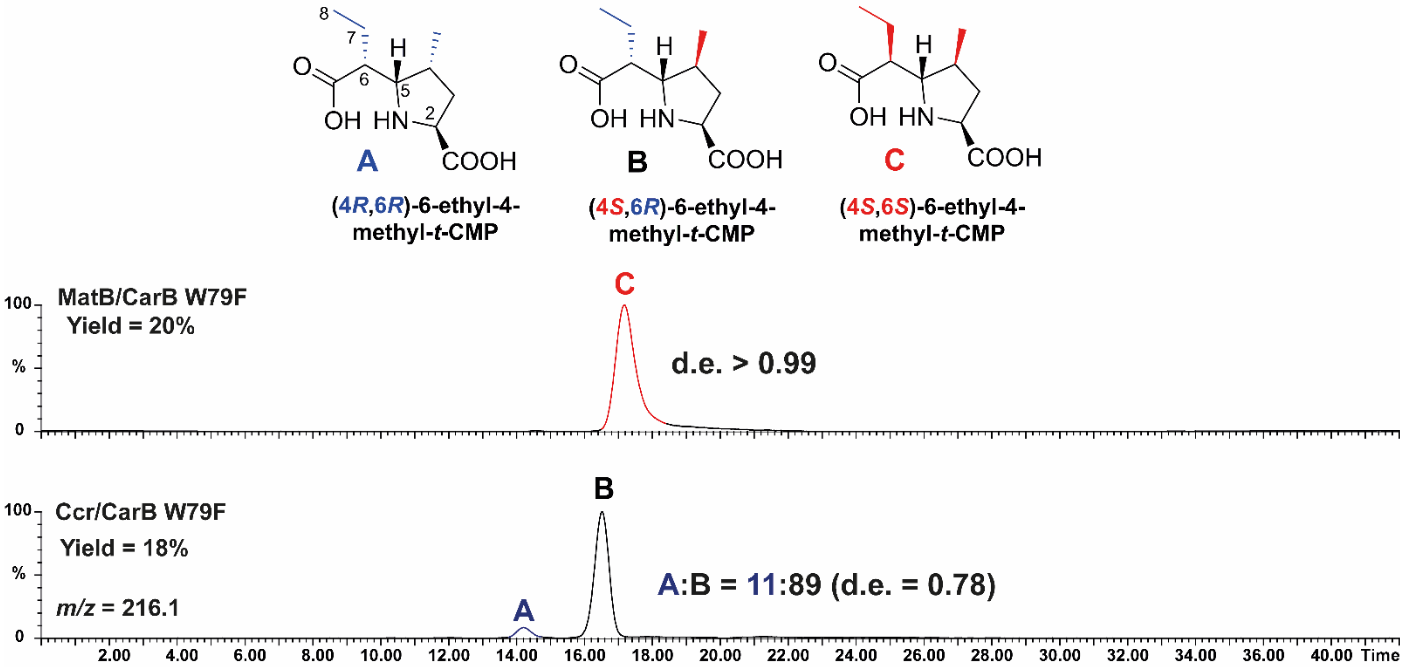

Supplementary Figure 16: Optimizing the stereoselectivity of CarB W79F catalysis. *The lower ion-extracted chromatogram* displays the formation of (4*R*,6*R*)- and (4*S*,6*R*)-6-ethyl-4-methyl-*t*-CMP epimers (in an 11:89 diastereomeric ratio) as a result of incubation of (*E*)-crotonyl-CoA and 4-methyl-L-GHP, in the presence of NADPH and sodium bicarbonate, as catalysed by tandem Ccr/CarB W79F catalysis. *The upper ion-extracted chromatogram* displays the selective formation of the (4*S*,6*S*)-6-ethyl-4-methyl-*t*-CMP epimer as a result of incubation of 2-ethylmalonic acid and 4-methyl-L-GHP, in the presence of ATP and Co-enzyme A, as catalysed by tandem MatB/CarB W79F catalysis. Note that incubation of C-2 epimeric ethylmalonyl-CoA and 4-methyl-L-GHP, as catalysed by CarB W79F, results in the formation of three observed products (A, B, and C) in a 24:59:17 ratio (Table 1, entry 7).

Stereochemical assignment of 4-methyl-6-alkyl-*t*-CMP stereoisomers (resulting from incubation of 4-methyl-L-GHP with C-2-alkylated malonic acid derivatives, under standard conditions, as catalysed by MatB/CarB W79A coupled system)

For the products analysed by NMR spectroscopy, the stereochemistry of C-4 was assigned as (*S*) based on the observation of a strong nOe between H-5 and the C-4 methyl group, together with the observation of a weak nOe between H-5 and H-4 (Supplementary Figures 18-21).

The assignment of C-6 as having the (*S*)-stereochemistry was based on a combination of coupling constant $J_{5,6}$ ~ 6-9 Hz (predicted Φ ~ 130-155°) and nOe data that revealed: (i) a moderate nOe correlation between H-5 and H-6 indicating a predominately anticlinal relationship between these two protons; (ii) The observation of a moderate nOe correlation between H-6 and H-4', together with a weak or no correlation between H-6 and H-4; (iii) The observation of a moderate to weak nOe correlation between H-7 and H-4, but not (or weaker) with H-4'. See below for full-annotated spectra of 4-methyl-6-alkyl-*t*-CMP stereoisomers prepared in this study.

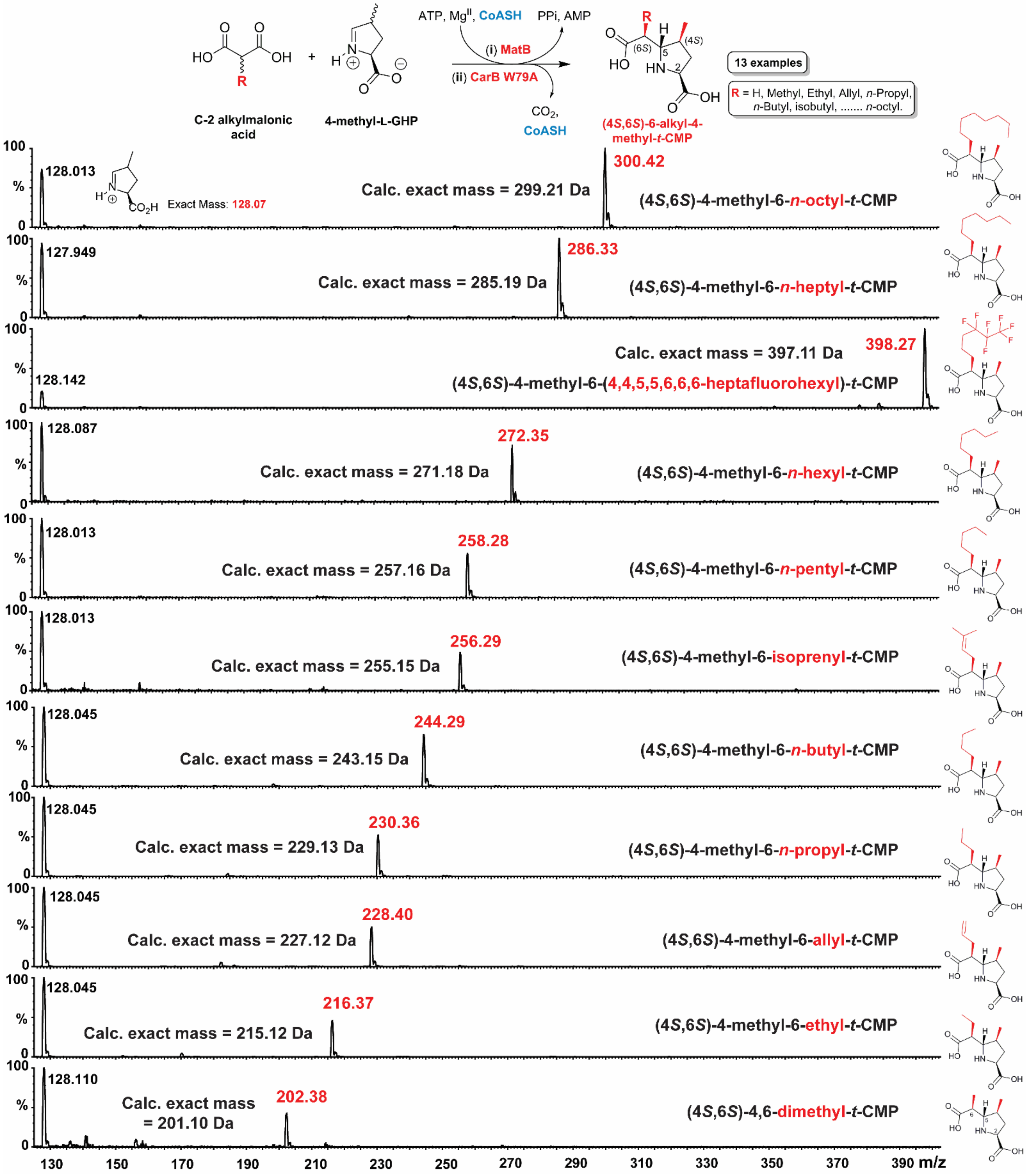

Supplementary Figure 17: Electrospray ionisation mass spectra (ESI+) displaying the ability of the coupled MatB/CarB W79A-catalysed reactions to generate diverse C-4 and C-6 alkylated *t*-carboxymethylproline (*t*-CMP) derivatives from 4-methyl-L-GHP and the corresponding C-2 alkylated malonic acid derivatives in the presence of the cofactors ATP and CoASH. Note the characteristic MS fragment at m/z = 128, resulting from loss of the side chain at C-5 of the *t*-CMP derivatives.

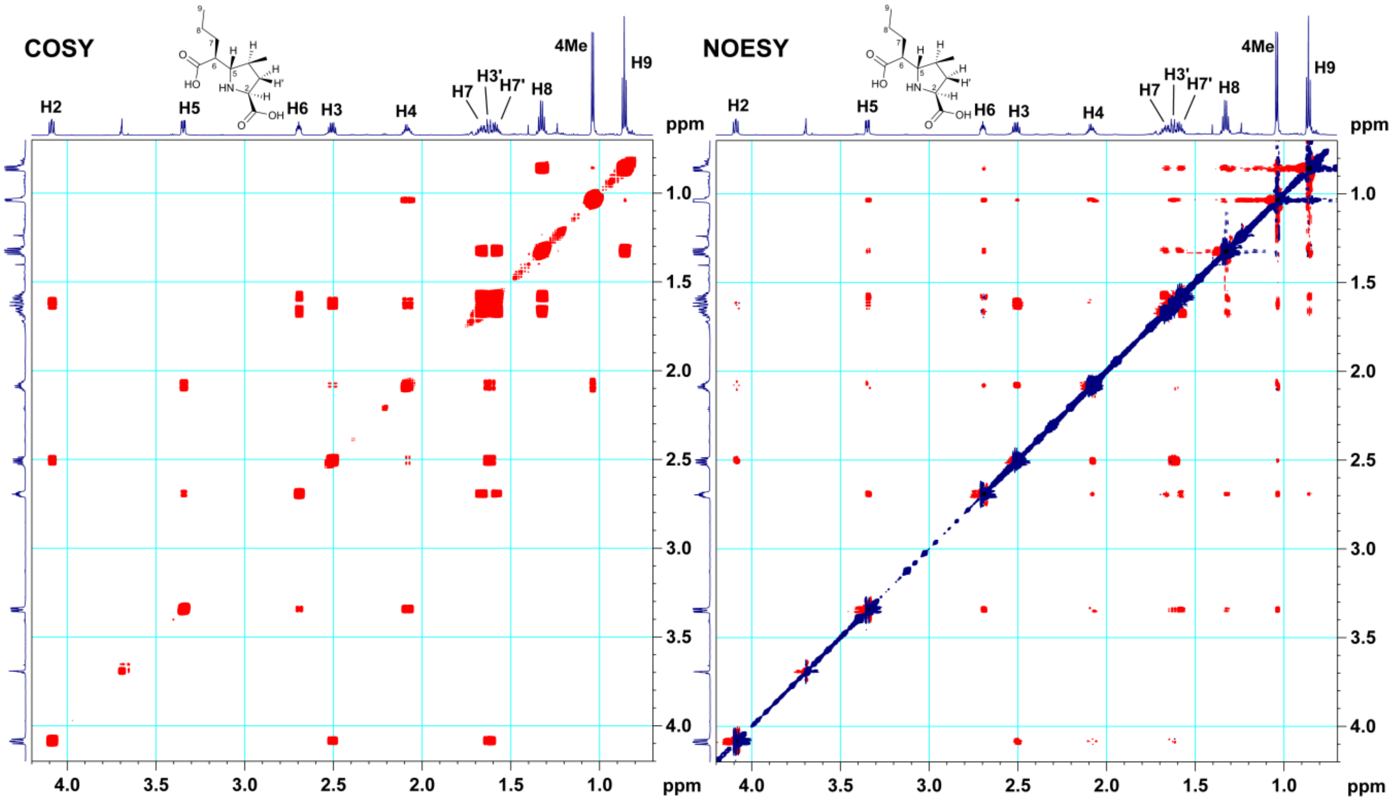

Supplementary Figure 18: **^{1}H-^{1}H COSY (left) and NOESY (right) spectra for (4*S*,6*S*)-4-methyl-6-*n*-propyl-*t*-CMP resulting from the incubation of C-2 *n*-propylmalonic acid and 4-methyl-L-GHP in the presence of MatB, CarB W79A and requisite co-substrates/co-factors.**

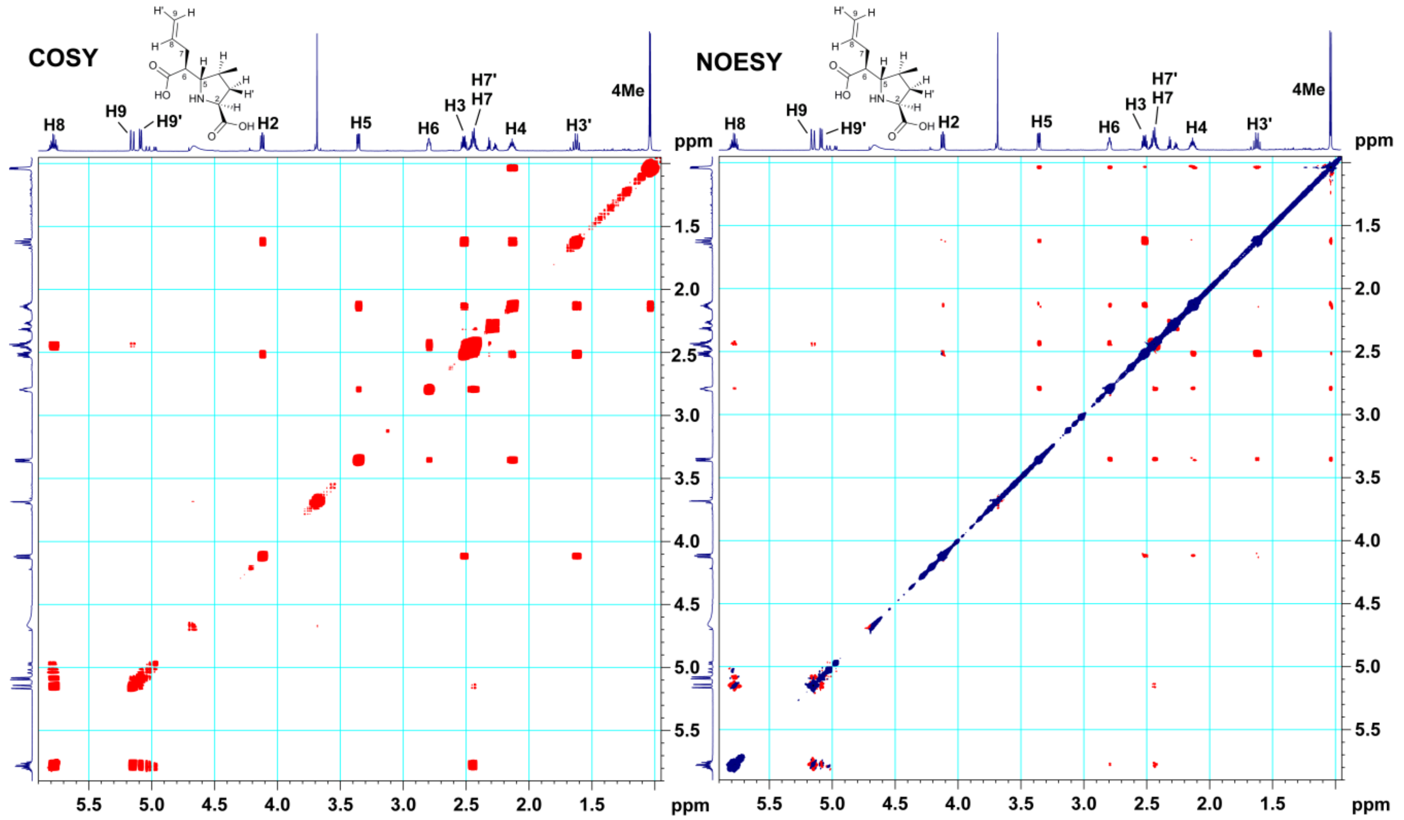

Supplementary Figure 19: **^{1}H-^{1}H COSY (left) and NOESY (right) spectra for (4*S*,6*S*)-6-Allyl-4-methyl-*t*-CMP resulting from the incubation of C-2 allylmalonic acid and 4-methyl-L-GHP in the presence of MatB, CarB W79A and requisite co-substrates/co-factors.**

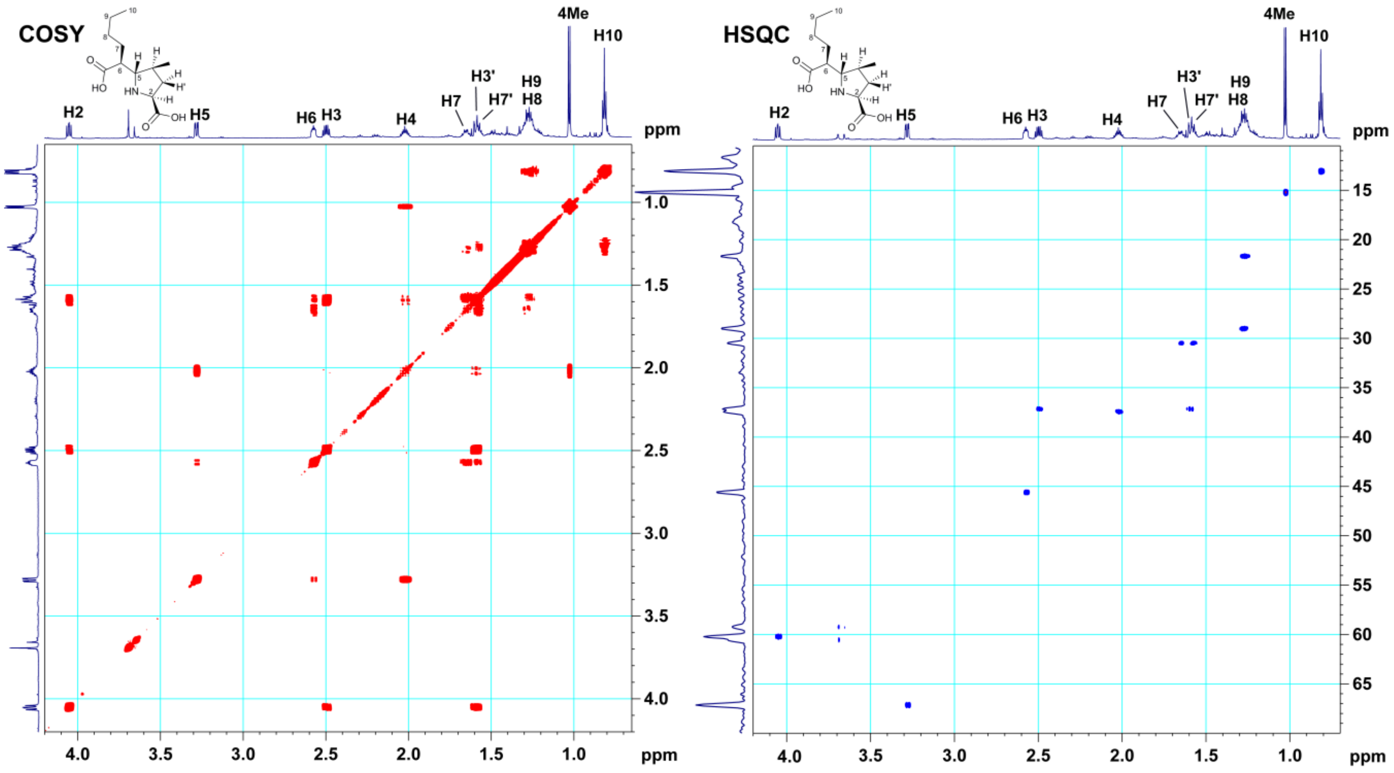

Supplementary Figure 20: **^{1}H-^{1}H COSY (left) and HSQC (right) spectra for (4*S*,6*S*)-6-*n*-butyl-4-methyl-*t*-CMP resulting from the incubation of C-2 *n*-butylmalonic acid and 4-methyl-L-GHP in the presence of MatB, CarB W79A and other required co-substrates/co-factors.**

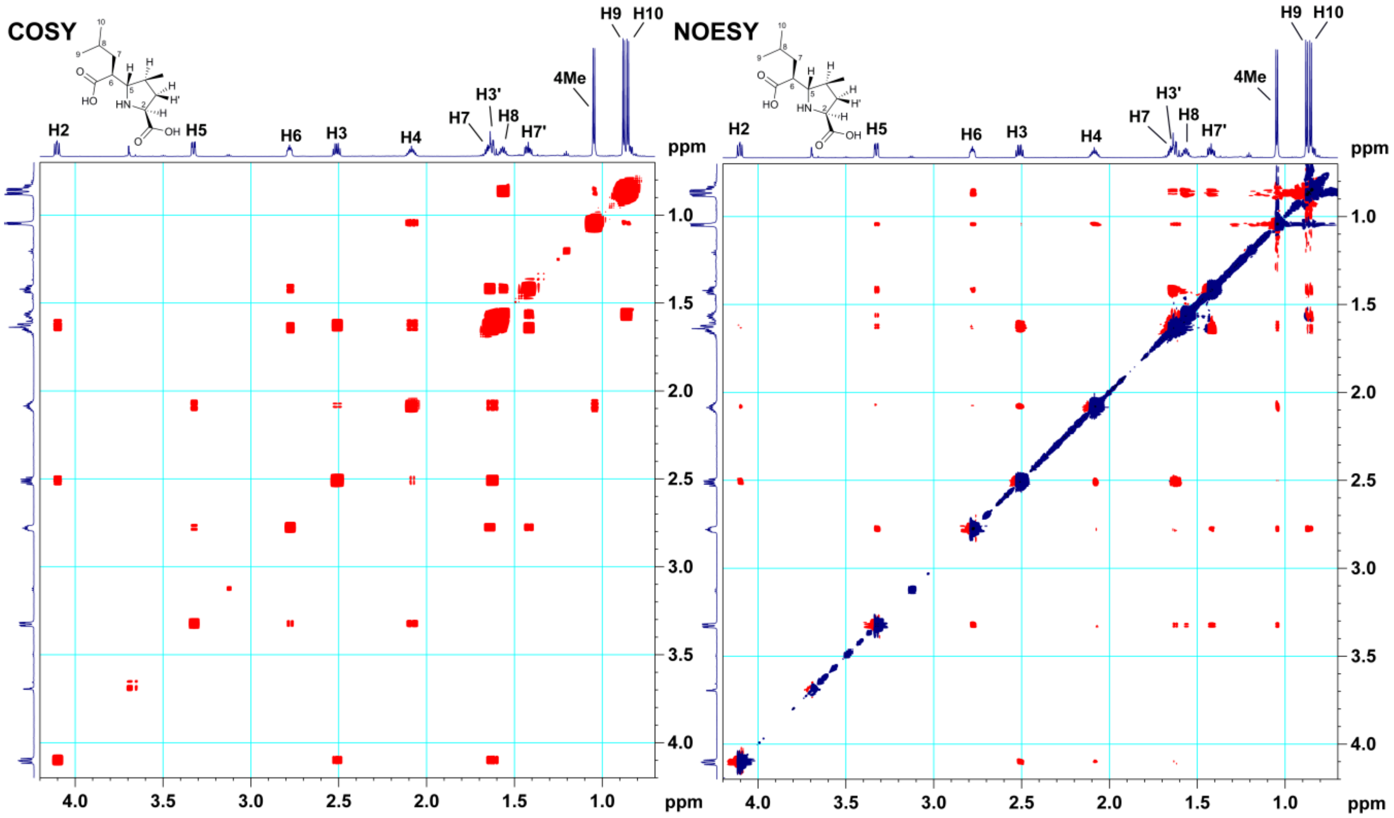

Supplementary Figure 21: **^{1}H-^{1}H COSY (left) and NOESY (right) spectra for (4*S*,6*S*)-6-isobutyl-4-methyl-*t*-CMP resulting from the incubation of C-2 isobutylmalonic acid and 4-methyl-L-GHP in the presence of MatB, CarB W79A and requisite co-substrates/co-factors.**

Incubation of 2-(2-cyanoethyl)malonic acid, L-GHP derivatives, MatB and CarB W79A for the production of (6*S*)-6-(2-cyanoethyl)-*t*-CMP derivatives

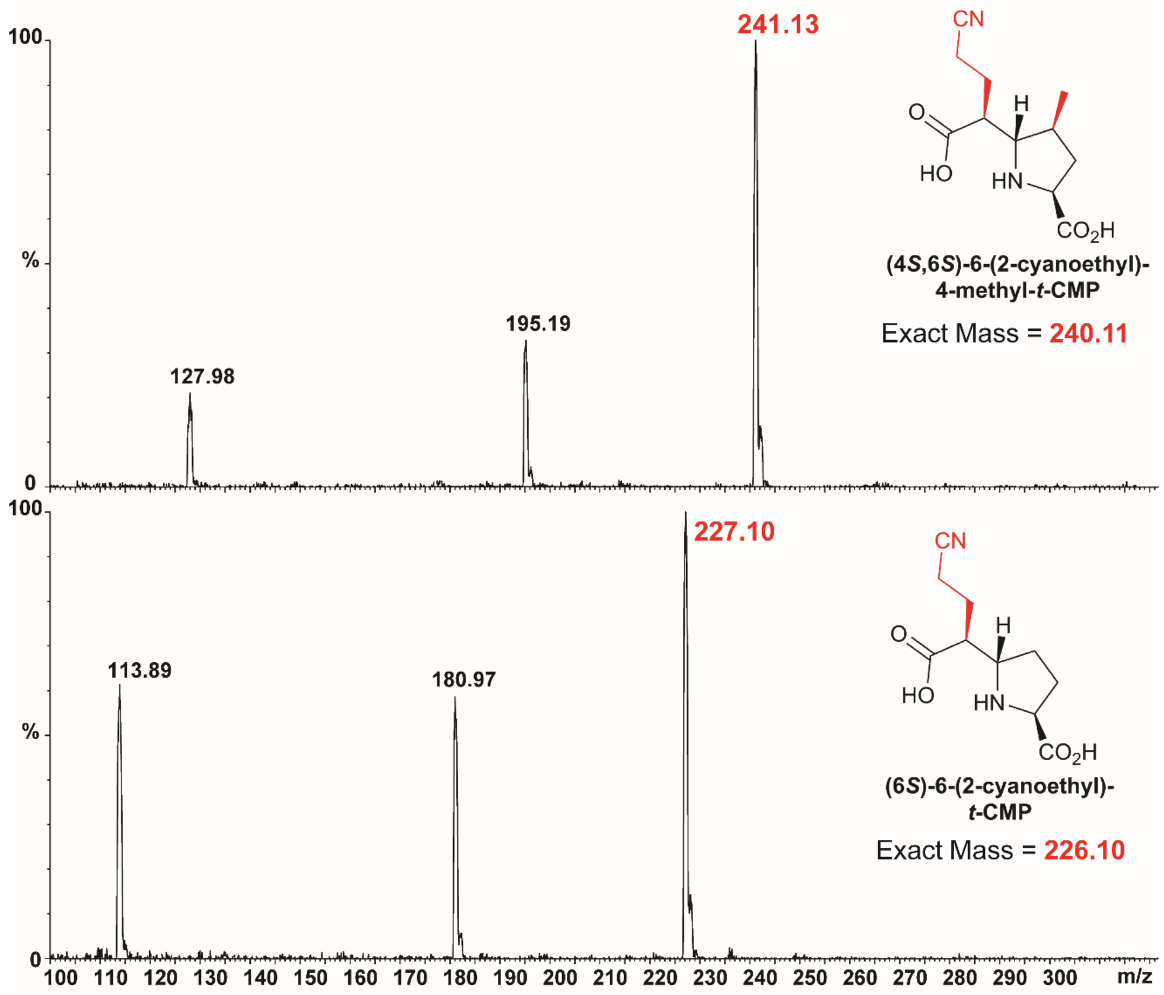

Supplementary Figure 22: Electrospray ionisation mass spectra (ESI+) displaying the formation of C-6 (2-cyanoethyl)-*t*-carboxymethylproline (*t*-CMP) derivatives from incubation of 2-(2-cyano)malonic acid and L-GHP/4-methyl-L-GHP as catalysed by the coupled MatB and CarB W79A in the presence of ATP and CoASH. Note the characteristic MS fragment at *m*/*z* = 114 and 128, resulting from loss of the C-5 side chain.

Stereochemical assignment of (4*S*,6*S*)-6-(2-cyanoethyl)-4-methyl-*t*-CMP (resulting from incubation of 4-methyl-L-GHP with 2-(2-cyanoethyl)malonic acid, as catalysed by MatB/CarB W79A coupled system)

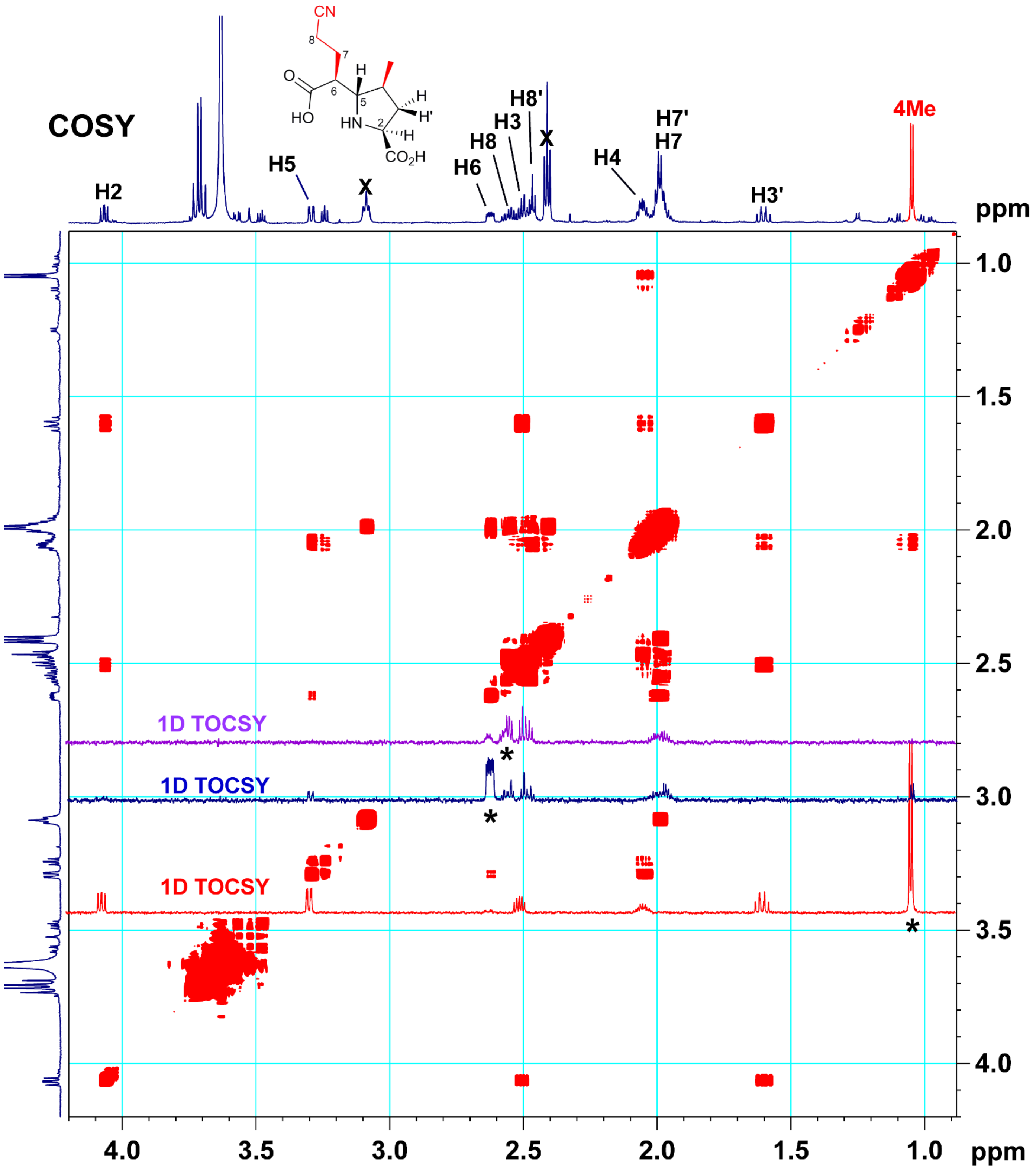

Supplementary Figure 23: NMR spectra for (4*S*,6*S*)-6-(2-cyanoethyl)-4-methyl-*t*-CMP resulting from incubation of 2-(2-cyanoethyl)malonic acid and 4-methyl-L-GHP with MatB, CarB W79A and requisite co-substrates/co-factors. The 1D-TOCSY spectra (τ_m = 150 ms) were generated by selective excitation of the asterisked protons.

Incubation of 2-(3-chloropropyl)malonic acid and L-GHP derivatives, MatB and CarB W79A for the production of (6*S*)-6-(3-chloropropyl)-*t*-CMP derivatives

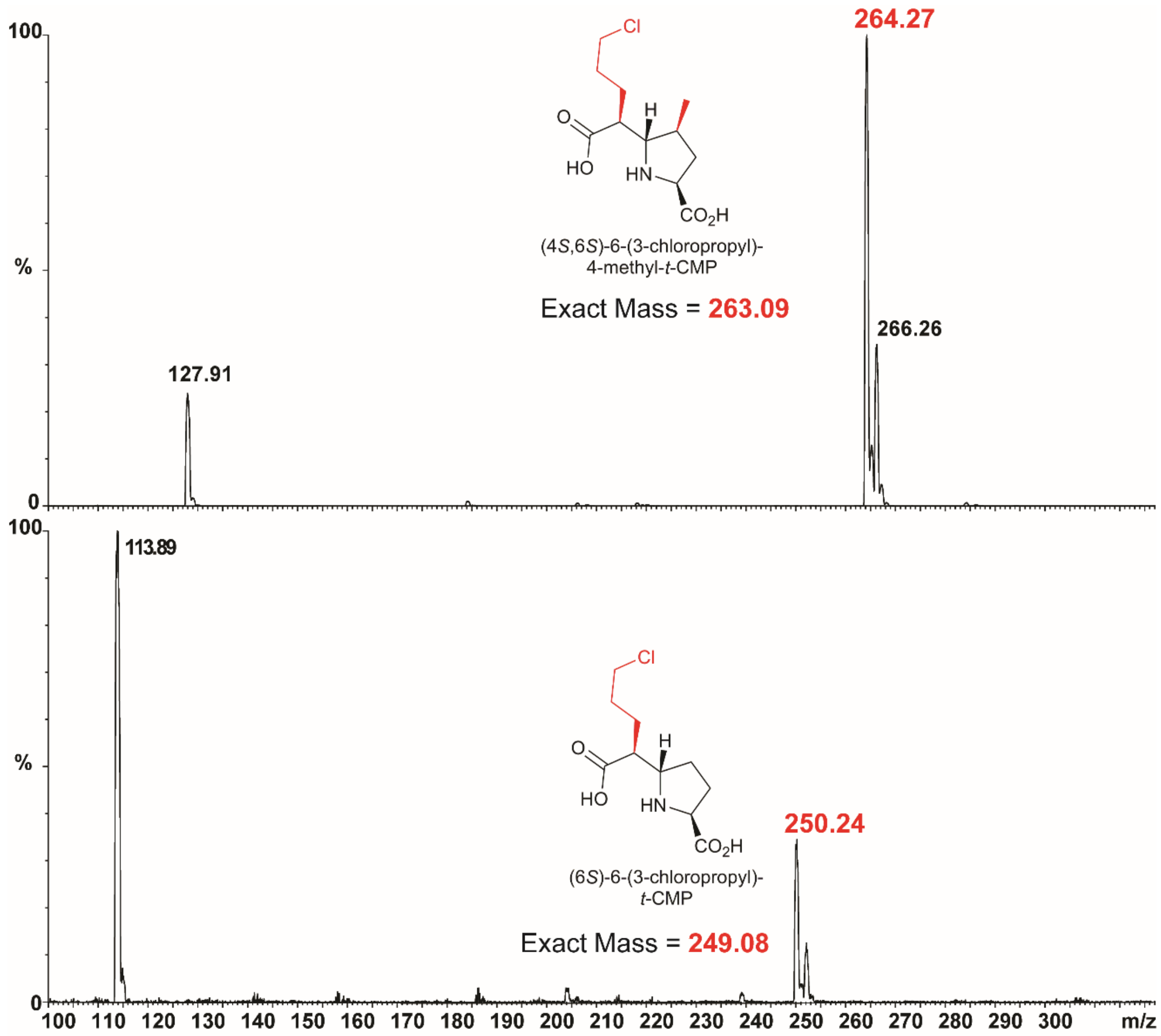

Supplementary Figure 24: Electrospray ionisation mass spectra (ESI+) supporting formation of C-6 (3-chloropropyl)-*t*-carboxymethylproline (*t*-CMP) derivatives from incubation of 2-(3-chloropropyl)malonic acid and L-GHP/4-methyl-L-GHP as catalysed by the coupled MatB and CarB W79A in the presence of the cofactors ATP and CoASH. Note the ratio of M^+ to $(M+2)^+$ = 1:0.3, characteristic for monochlorinated small molecules. Also, note the characteristic MS fragment at *m*/*z* = 114 and 128, resulting from loss of the side chain at C-5.

Stereochemical assignment of (4*S*,6*S*)-6-(3-chloropropyl)-4-methyl-*t*-CMP (resulting from incubation of 4-methyl-L-GHP with 2-(3-chloropropyl)malonic acid, under standard conditions, as catalysed by MatB/CarB W79A coupled system)

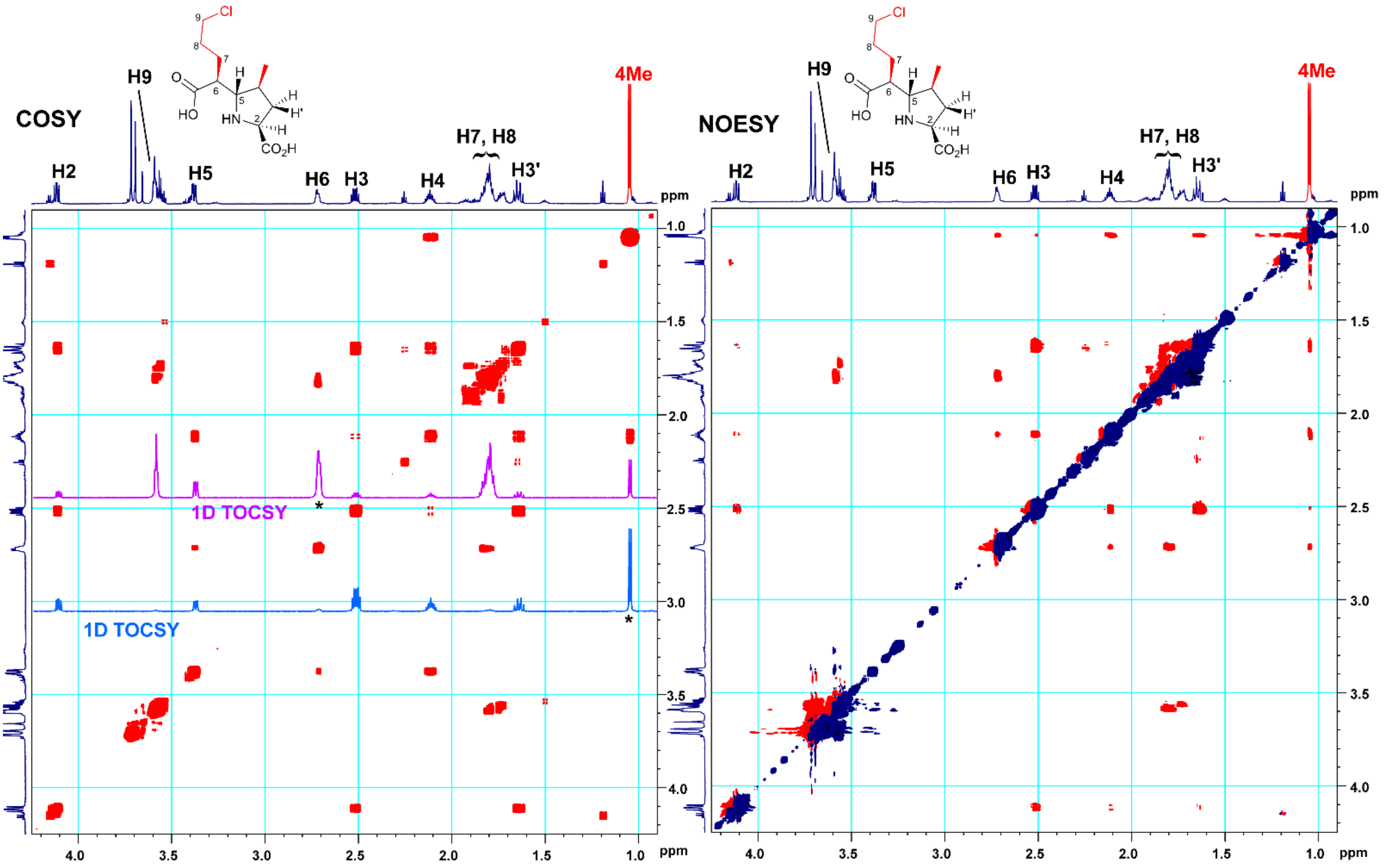

Supplementary Figure 25: NMR spectra for (4*S*,6*S*)-6-(3-chloropropyl)-4-methyl-*t*-CMP resulting from the incubation of 2-(3-chloropropyl)malonic acid and 4-methyl-L-GHP in the presence of MatB, CarB W79A and other required co-substrates/co-factors. The 1D-TOCSY spectra (τ_m = 150 ms) were generated by selective excitation of the asterisked protons.

Incubation of 2-methoxymalonic acid, L-GHP derivatives, MatB and CMPS variants for the production of 6-methoxy-*t*-CMP derivatives

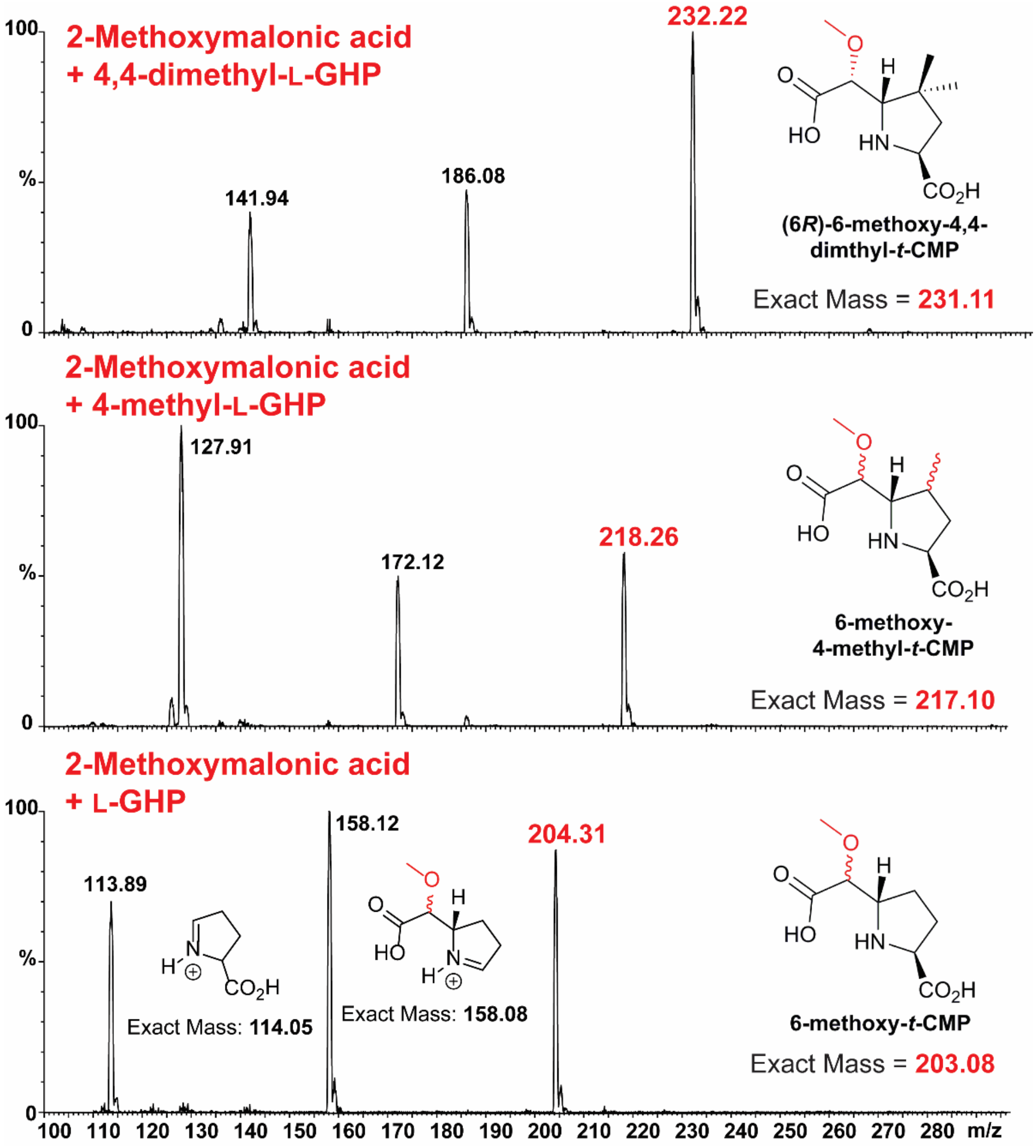

Supplementary Figure 26: Electrospray ionisation mass spectra (ESI+) displaying the formation of 6-methoxy-*t*-carboxymethylproline (*t*-CMP) derivatives resulting from incubation of 2-methoxymalonic acid and L-GHP, 4-methyl-L-GHP or 4,4-dimethyl-L-GHP (in order from bottom to top) as catalysed by the tandem MatB/CarB W79F system, in the presence of MatB cofactors (ATP and CoASH). Note the characteristic MS fragments of *t*-CMP derivatives corresponding to loss of the side chain at C-5 or decarboxylation of the proline core.

Stereochemical assignment of (6*R*)-6-methoxy-4,4-dimethyl-*t*-CMP (resulting from incubation of 4,4-dimethyl-L-GHP and 2-methoxymalonic acid as catalysed by MatB/CarB W79F)

The stereochemistry at C-6 was assigned as (*R*) on the basis of the following observations:

- A $J_{5,6}$ value of 7.6 Hz (predicted $\Phi \sim 139^{o}$) together with a weak nOe correlation between H-5 and H-6 implying an anticlinal relationship.
- A strong nOe correlation between H-6 and the methyl group(s) at C-4.
- A lack of observation (very weak) nOe correlation between the methoxy group and the methyl group(s) at C-4.

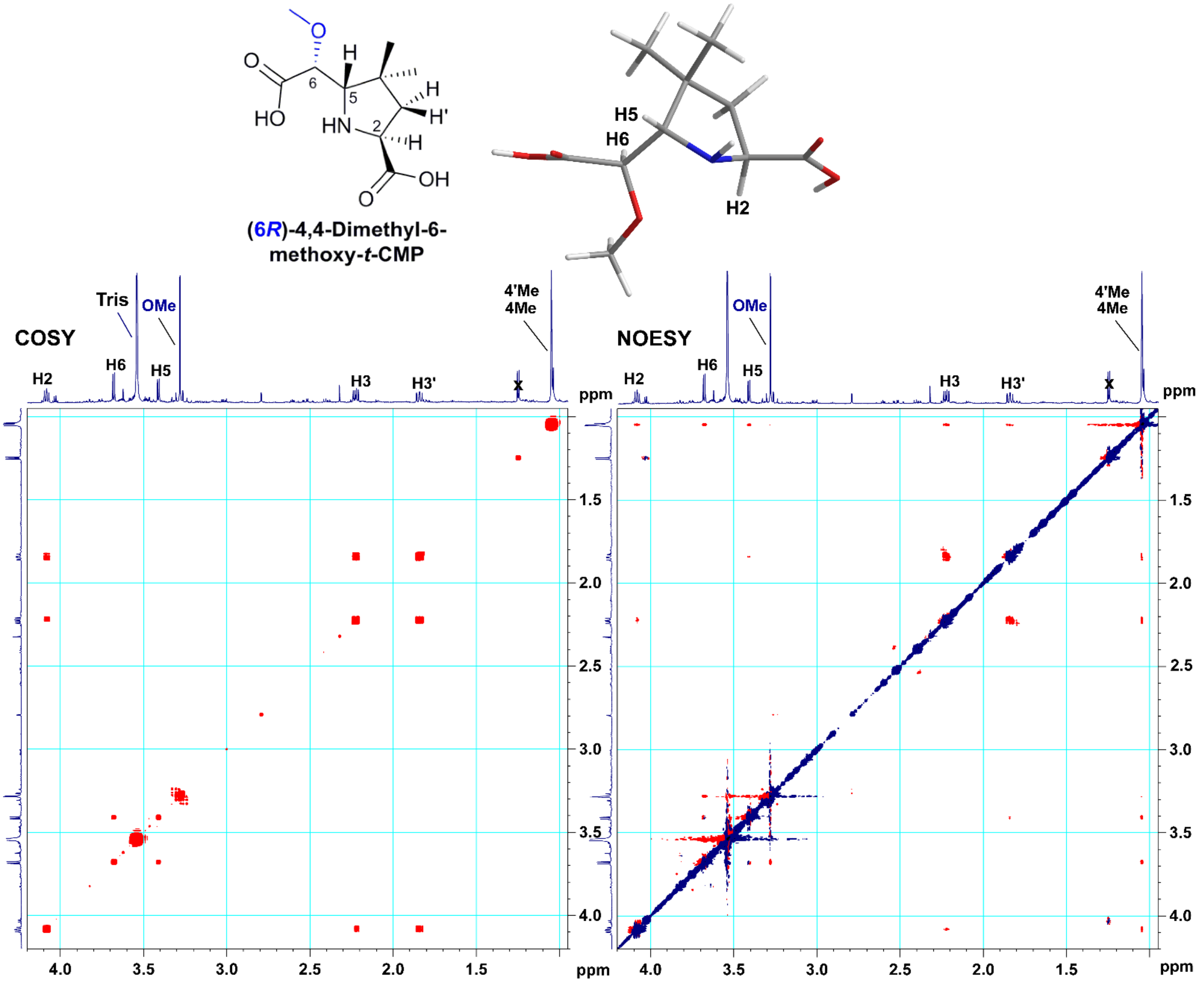

Supplementary Figure 27: ^{1}H-^{1}H COSY and NOESY spectra for (6*R*)-4,4-dimethyl-6-methoxy-*t*-CMP produced from incubation of 4,4-dimethyl-L-GHP and 2-methoxymalonic acid by coupled MatB/CarB W79F catalysis. The appended energy minimized 3D model, generated by ChemBio3D, is based on the coupling constant and 2D NOESY spectral data.

Stereochemical assignment of 6-methoxy-4-methyl-*t*-CMP stereoisomers (resulting from incubation of C-4 epimeric 4-methyl-L-GHP and 2-methoxymalonic acid as catalysed by MatB/CMPSs)

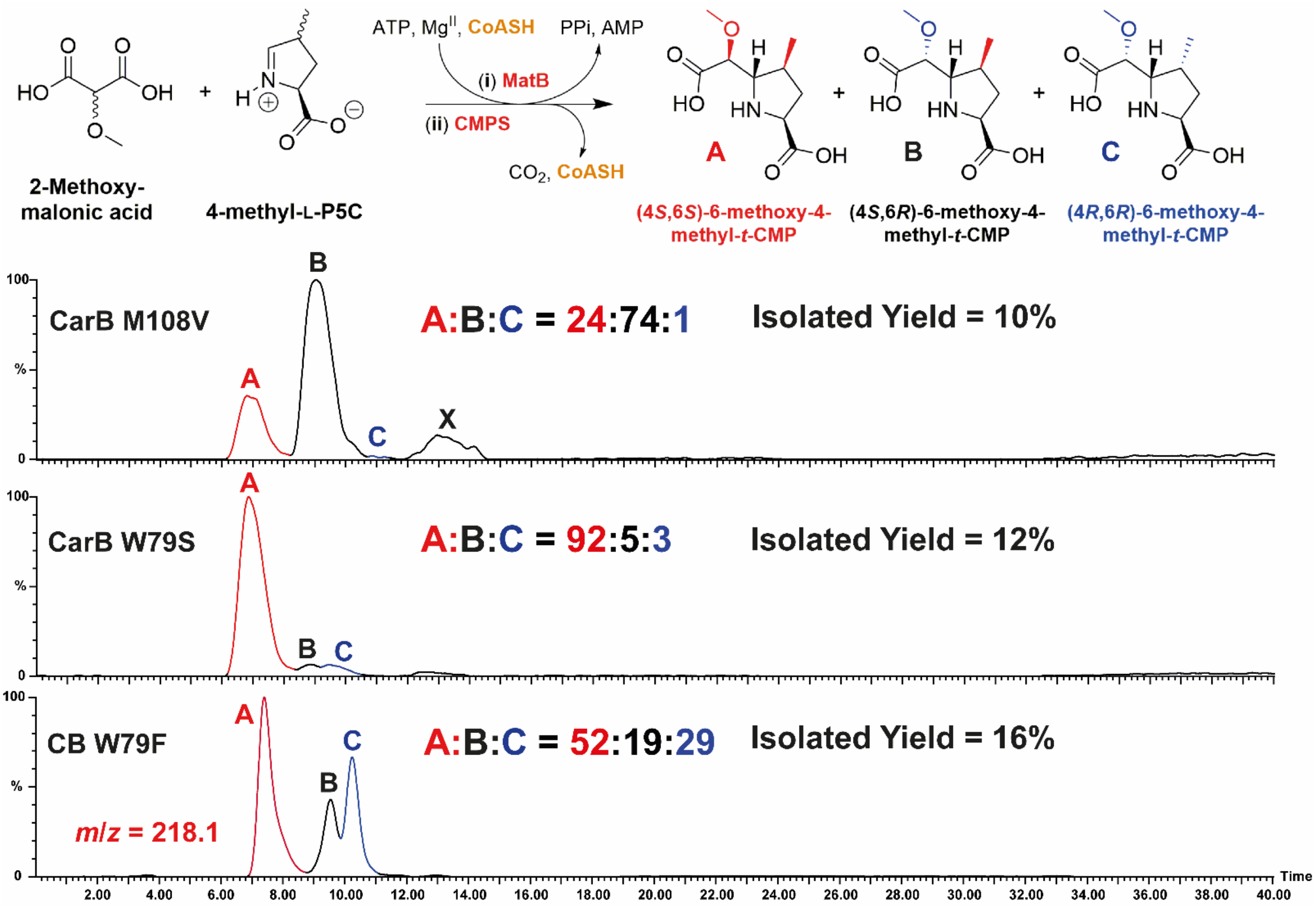

Supplementary Figure 28: Formation of 6-methoxy-4-methyl-*t*-CMP stereoisomers via incubation of C-4 epimeric 4-methyl-L-GHP and 2-methoxymalonic acid with MatB/CMPSs, in the presence of MatB cofactors (ATP and CoASH). The ion extracted LC-MS chromatograms (ESI+) display the different selectivities of the coupled Ccr/CMPSs. The Internal standard (*p*-aminosalicyclic acid) is not shown for clarity.

Three products were detected and labelled **A**, **B** and **C**, according to their order of elution. For compound **A**, the stereochemistry at C-4 was assigned as (*S*) based on the observation of a strong nOe between H-5 and the C-4 methyl group, together with the absence of an nOe between the methyl group at C-4 and H-2. The stereochemistry at C-6 was assigned as (*S*) on the basis of the following observations:

- A $J_{5,6}$ value of 2.9 Hz (predicted Φ ~ 44°) together with a strong nOe correlation between H-5 and H-6 indicating a synclinal arrangement for these two protons.
- The lack of observation of an nOe correlation either between H-6 and H-4 or between H-6 and the methyl group at C-4.
- The lack of observation of an nOe correlation neither between the methoxy group and H-4 nor between the methoxy group and the methyl group at C-4.

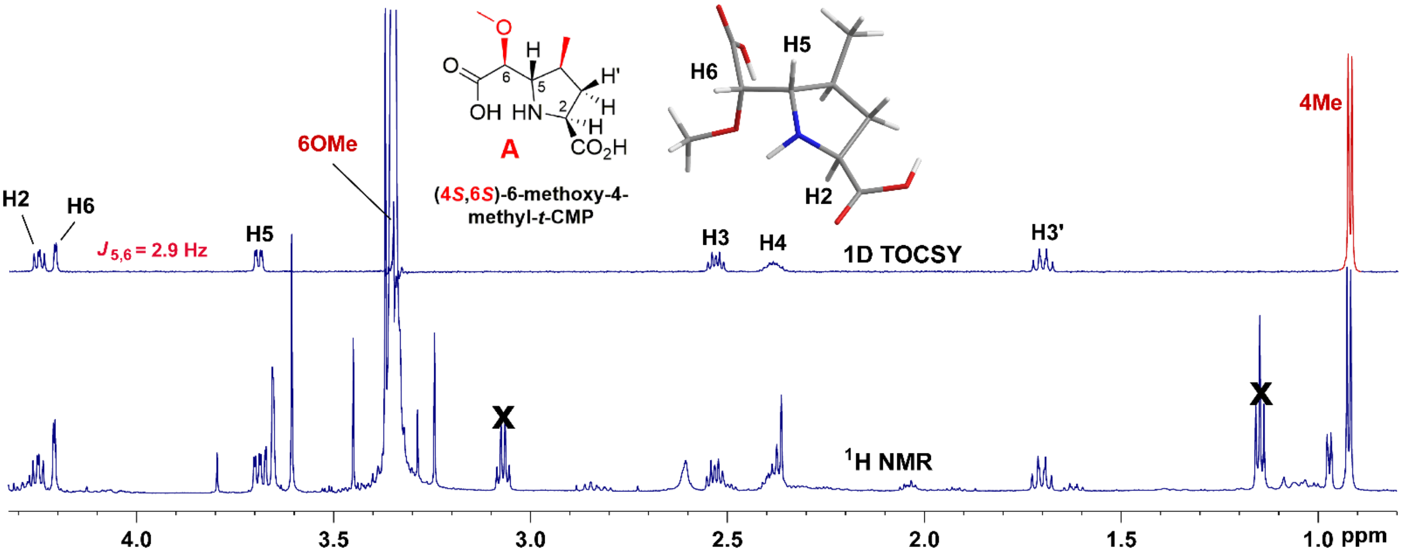

Supplementary Figure 29: NMR spectra for the early eluting diastereomer of 6-methoxy-4-methyl-*t*-CMP resulting from the incubation of 2-methoxymalonic acid and 4-methyl-L-GHP in the presence of MatB, CarB W79F and other required co-substrates/co-factors. The 1D-TOCSY spectrum (τ_m = 150 ms) was generated by selective excitation of the methyl group protons at C-4. The appended energy minimized 3D model, generated by ChemBio3D, is based on the coupling constant and 2D NOESY spectral data (Supplementary Figure 26).

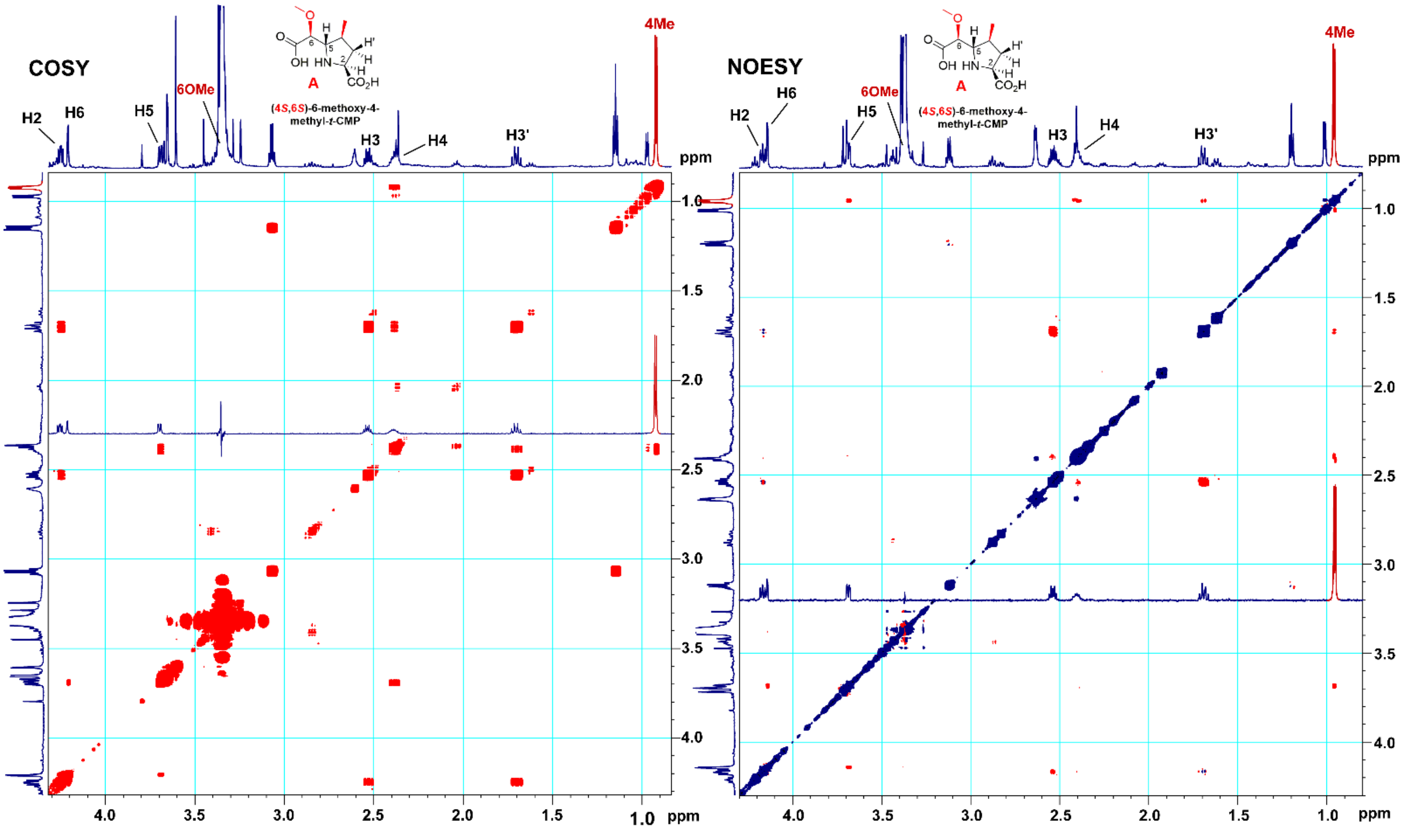

Supplementary Figure 30: NMR spectra for the first eluting stereoisomer of 6-methoxy-4-methyl-*t*-CMP resulting from incubation of 2-methoxymalonic acid and 4-methyl-L-GHP with MatB, CarB W79F and requisite co-substrates/co-factors. The appended 1D-TOCSY spectrum (τ_m = 150 ms) was generated by selective excitation of the methyl group protons at C-4.

For compound **B**, the stereochemistry at C-4 was assigned as (*S*) based on the observation of a strong nOe correlation between H-5 and the C-4 methyl group coupled to a weak nOe correlation between H-2 and H-4. The stereochemistry at C-6 was assigned as (*R*) based on the following observations:

- A $J_{5,6}$ value of 6.9 Hz (predicted Φ ~ 134°) together with a weak nOe correlation between H-5 and H-6 implying an anticlinal relationship.
- A weak nOe correlation between H-6 and the methyl group at C-4.
- The lack of observation of nOe correlations either between the methoxy group and H-4 or between the methoxy group and the methyl group at C-4.

For compound **C**, the stereochemistry of C-4 was assigned as (*R*) based on the observation of a strong nOe between H-2 and the C-4 methyl group together with the observation of a strong nOe between H-5 and H-4. The stereochemistry at C-6 was assigned as (*R*) on the basis of the following observations:

- A $J_{5,6}$ value of 7.4 Hz (predicted Φ ~ 137°) together with a weak nOe correlation between H-5 and H-6 implying an anticlinal relationship.
- A strong nOe correlation between H-6 and the methyl group at C-4.
- A weak nOe correlation between the methoxy group and the methyl group at C-4.

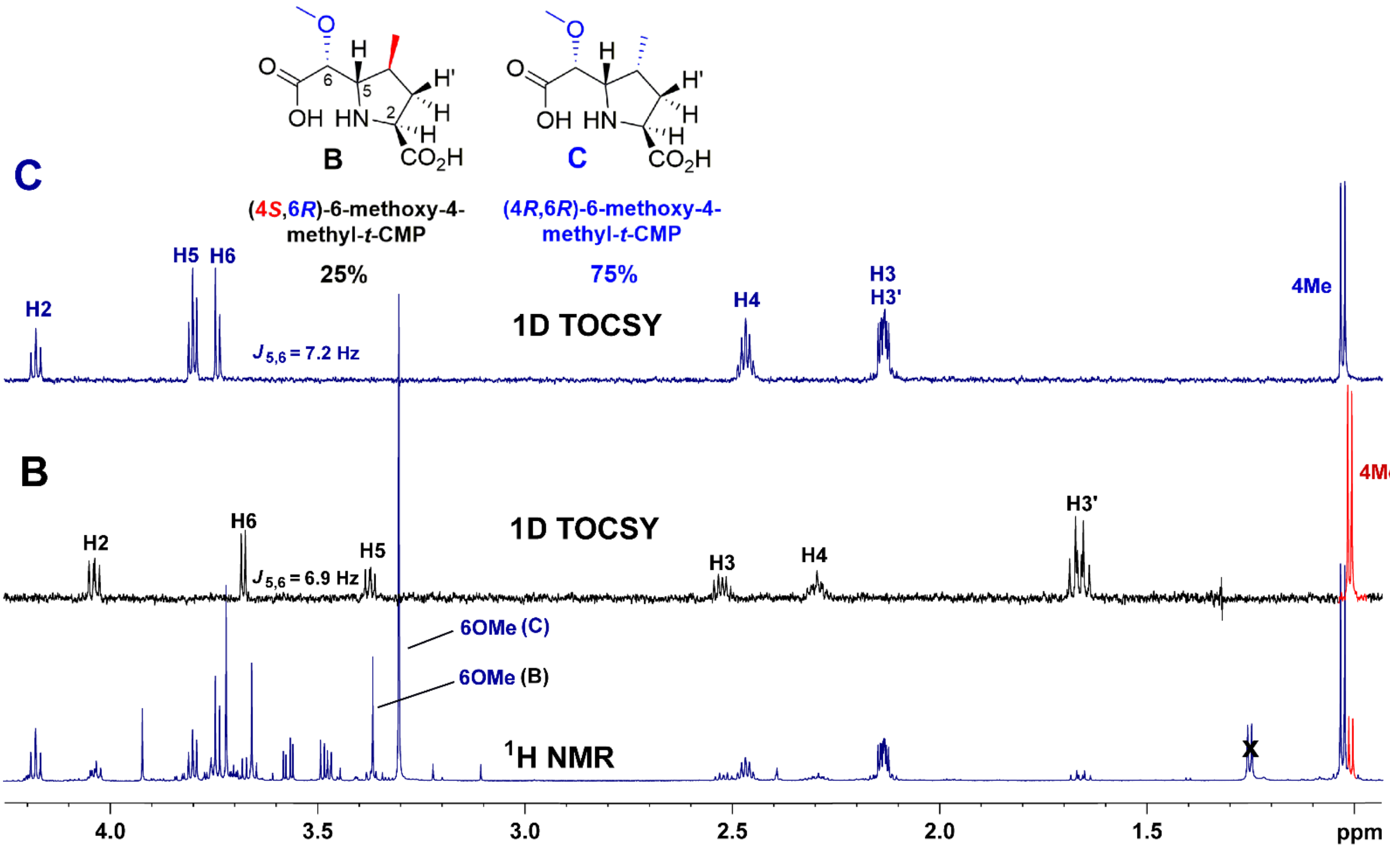

Supplementary Figure 31: NMR spectra for the two later-eluting mixture of stereoisomers of 6-methoxy-4-methyl-*t*-CMP resulting from the incubation of 2-methoxymalonic acid and 4-methyl-L-GHP with MatB/CarB W79F in the presence of requisite co-substrates/co-factors. The 1D-TOCSY spectra (τ_m = 150 ms) were generated by selective excitation of the protons of the respective methyl group at C-4.

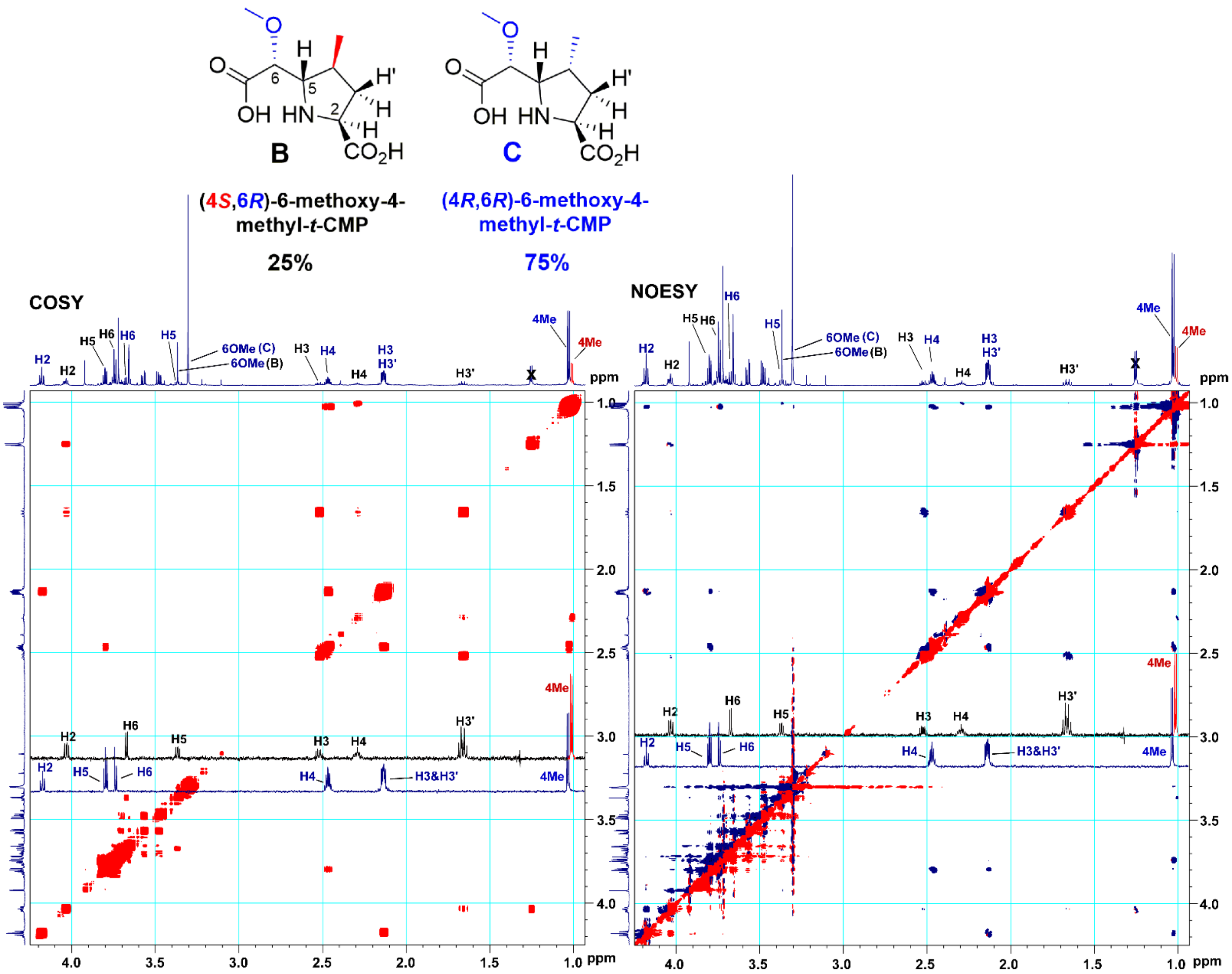

Supplementary Figure 32: NMR spectra for the later-eluting mixture of stereoisomers of 6-methoxy-4-methyl-*t*-CMP resulting from incubation of 2-methoxymalonic acid and 4-methyl-L-GHP with MatB, CarB W79F and requisite co-substrates/co-factors. The appended 1D-TOCSY spectra (τ_m = 150 ms) were generated by selective excitation of the protons of the respective methyl group at C-4.

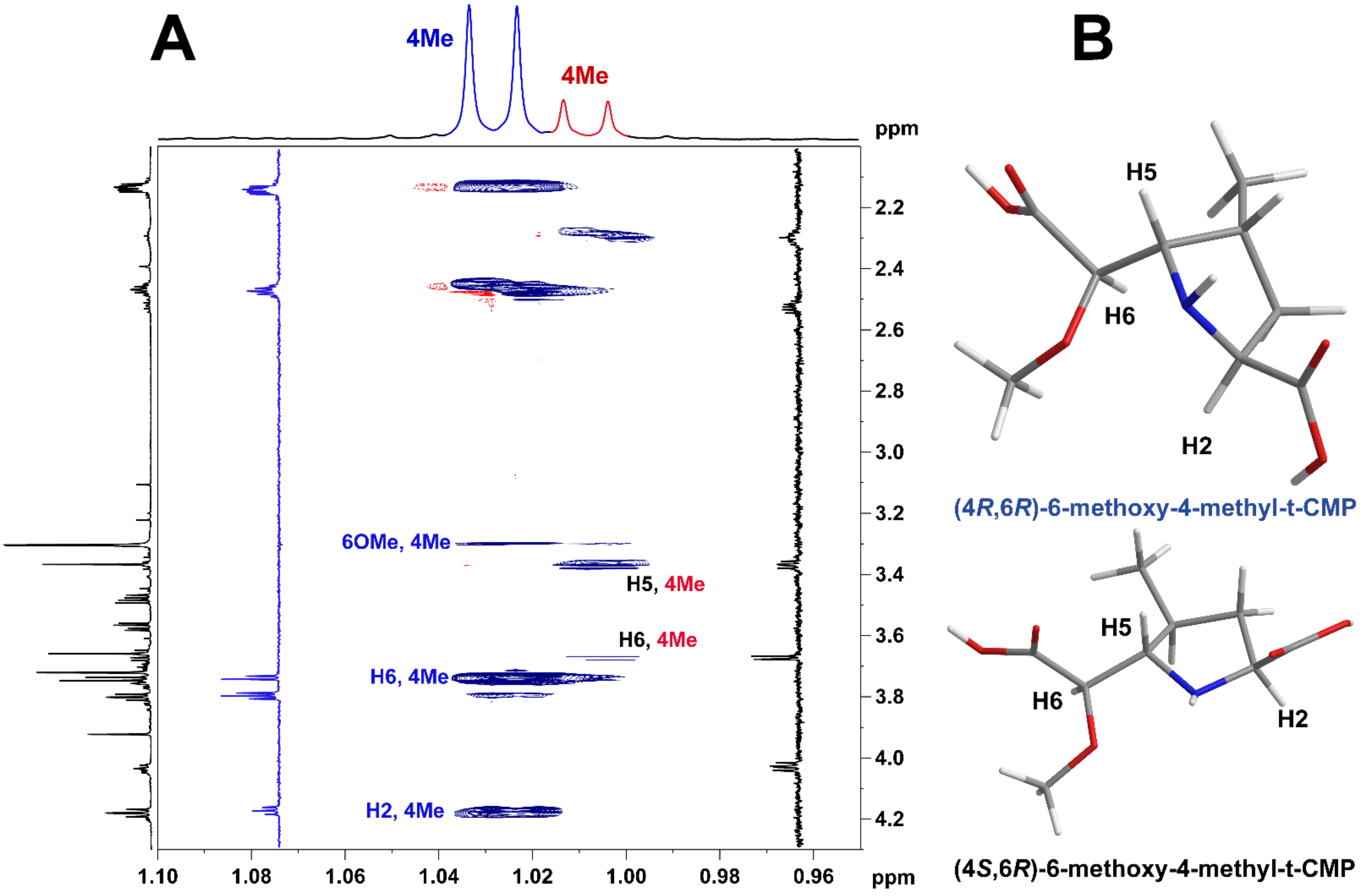

Supplementary Figure 33: The two later-eluting epimers of 6-methoxy-4-methyl-*t*-CMP resulting from the incubation of 2-methoxymalonic acid and 4-methyl-L-GHP in the presence of MatB, CarB W79F and other required co-substrates/co-factors. A: Expansion of the 2D NOESY spectrum of the mixture. The appended 1D TOCSY spectra are added for clarity; **B**: Energy minimized 3D models of the two epimers based on the coupling constant and 2D NOESY spectral data, generated using ChemBio3D.

Conversion of some of the *t*-CMP derivatives, produced by CMPS catalysis, into bicyclic β-lactams by CarA catalysis

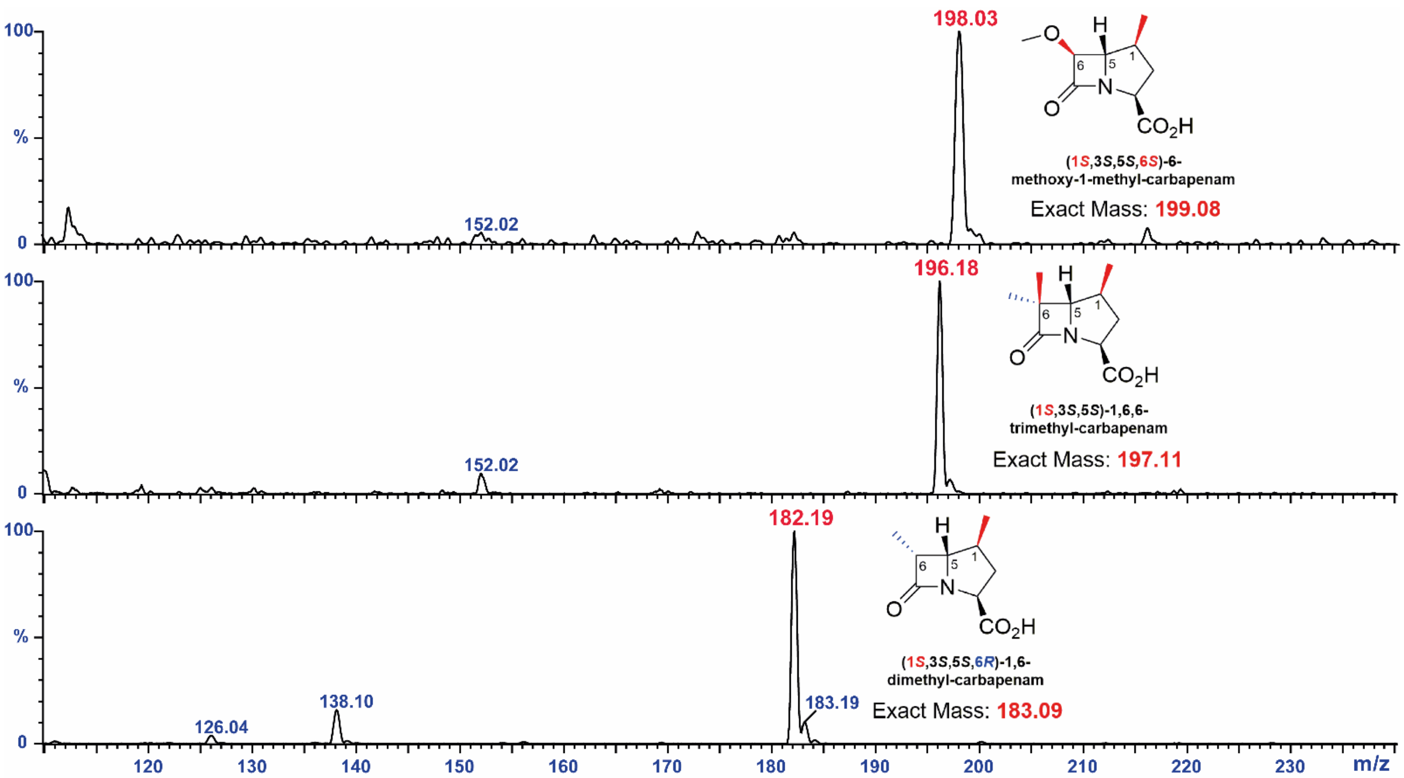

Supplementary Figure 34: Electrospray ionisation mass spectra (ESI-) supporting formation of the shown carbapenams from the corresponding *t*-CMP derivatives by CarA catalysis, under standard assay conditions.

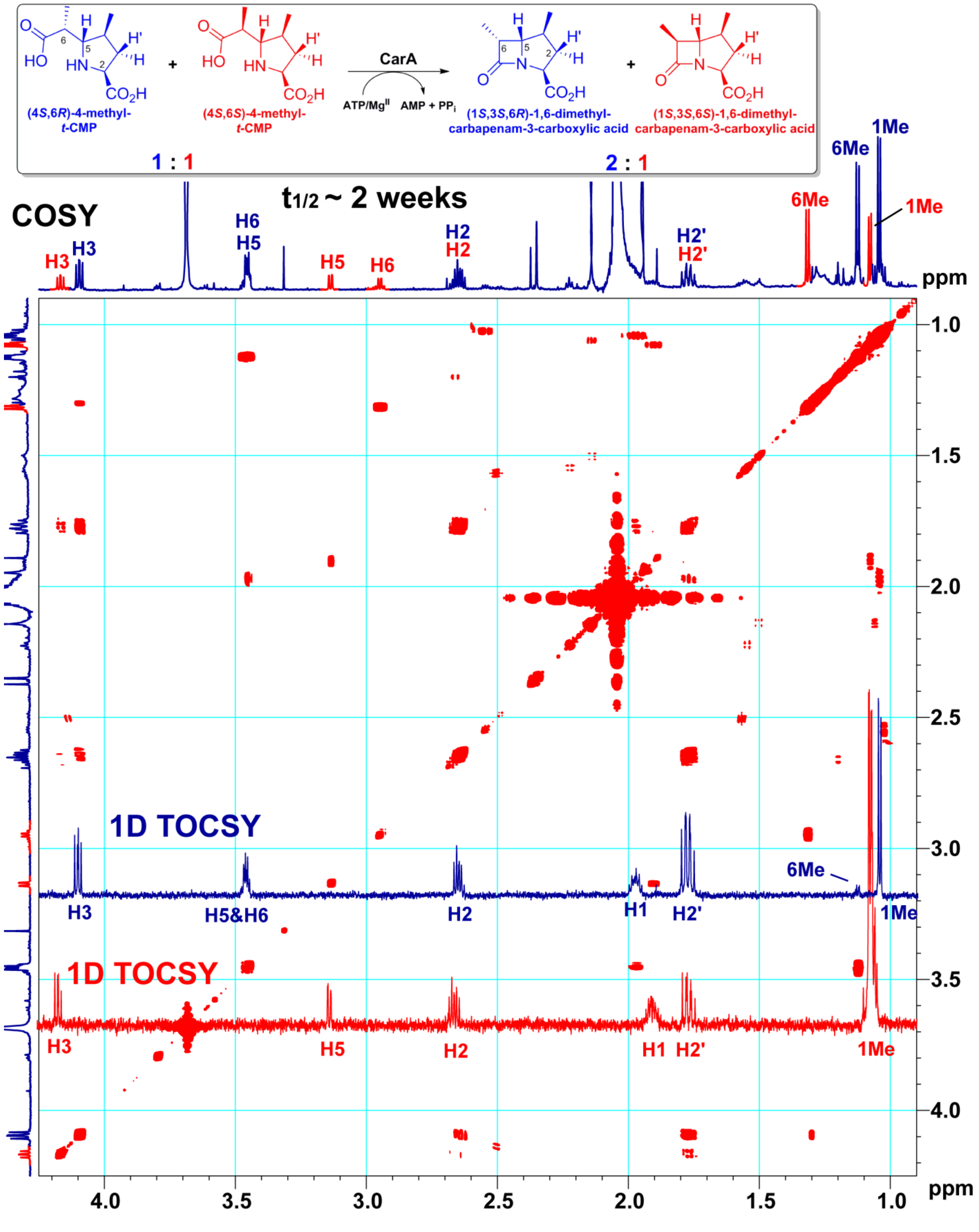

Supplementary Figure 35: **Formation of 1,6-dimethyl-carbapenam-3-carboxylate epimers by carbapenam synthetase (Car A) catalysis**. Note the preference of CarA for the (4*S*,6*R*)-4,6-dimethyl-*t*-CMP epimer, to the (4*S*,6*S*)-4,6-dimethyl-*t*-CMP epimer, as a substrate. The *trans*-relationship of H5 and H6 in the case of (1*S*,3*S*,5*S*,6*S*)-4,6-dimethyl-carbapenam-3-carboxylic acid was assigned on the basis of the $J_{5,6}$ = 1.2 Hz (typical for β-lactams with H5 and H6 in a *trans*-relationship).[13] Similarly, a $J_{5,6}$ = 5 Hz confirmed the *cis*-relationship of H5 and H6 in the case of (1*S*,3*S*,5*S*,6*R*)-epimer. The stability of the resultant carbapenams to hydrolysis is noteworthy (the $t_{1/2}$ of the products was determined by NMR).

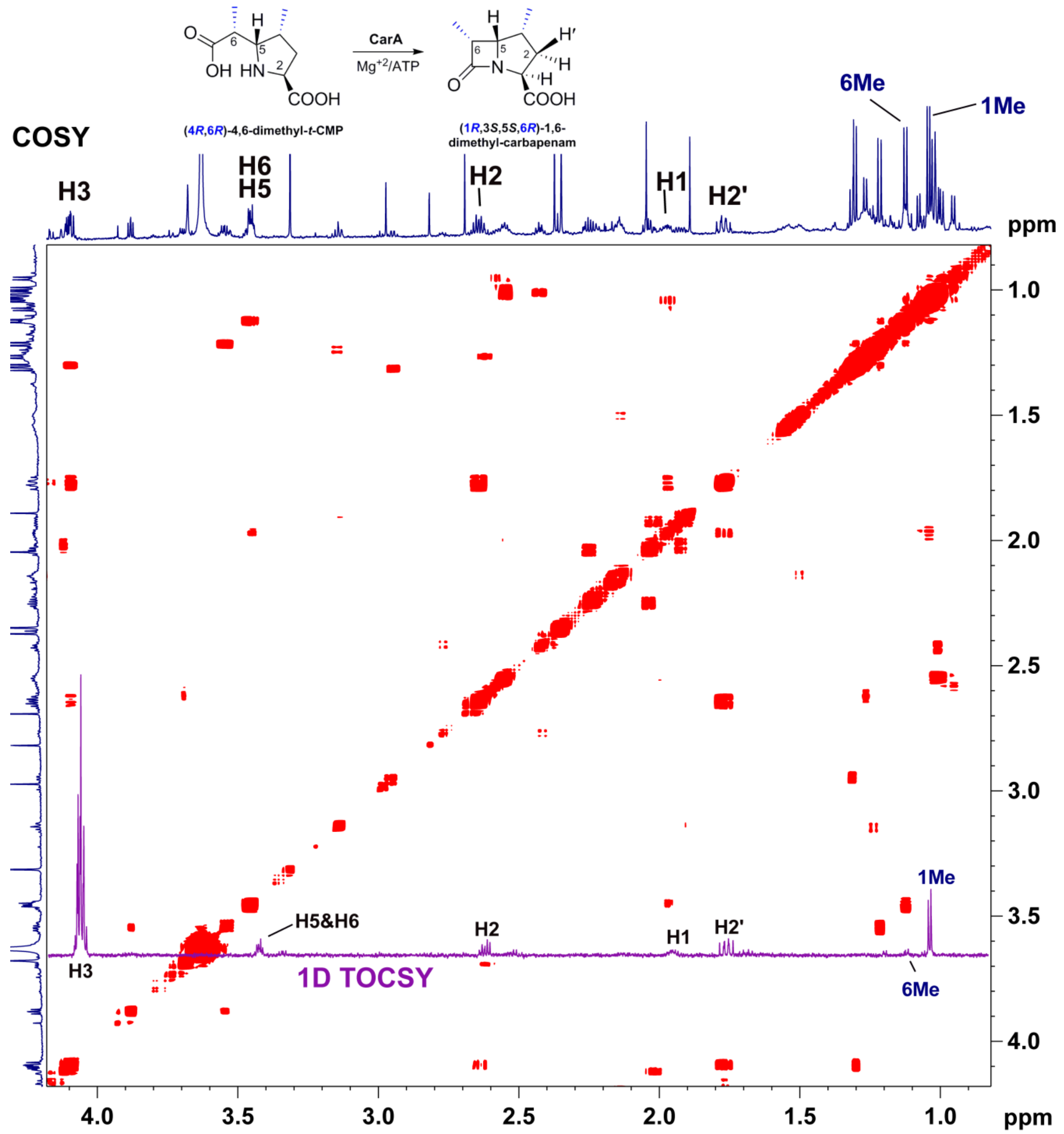

Supplementary Figure 36: NMR spectra for the (1*R*,3*S*,5*S*,6*R*)-4,6-dimethyl-carbapenam-3-carboxylic acid produced by incubation of (4*R*,6*R*)-4,6-dimethyl-*t*-CMP with ATP/Mg^{II} and CarA. The 1D-TOCSY spectrum (τ_m = 150 ms) was generated by selective excitation of the H3 proton. The stereochemistry at C-6 was assigned/confirmed as (*R*) on the basis of the $J_{5,6}$ = 5.6 Hz (typical for β-lactams with H5 and H6 in a *cis*-relationship).[13] The value of the $J_{5,6}$ coupling constant was calculated from the 1D-TOCSY spectrum (H5 and H6 overlap in the 1D ^{1}HNMR spectrum.

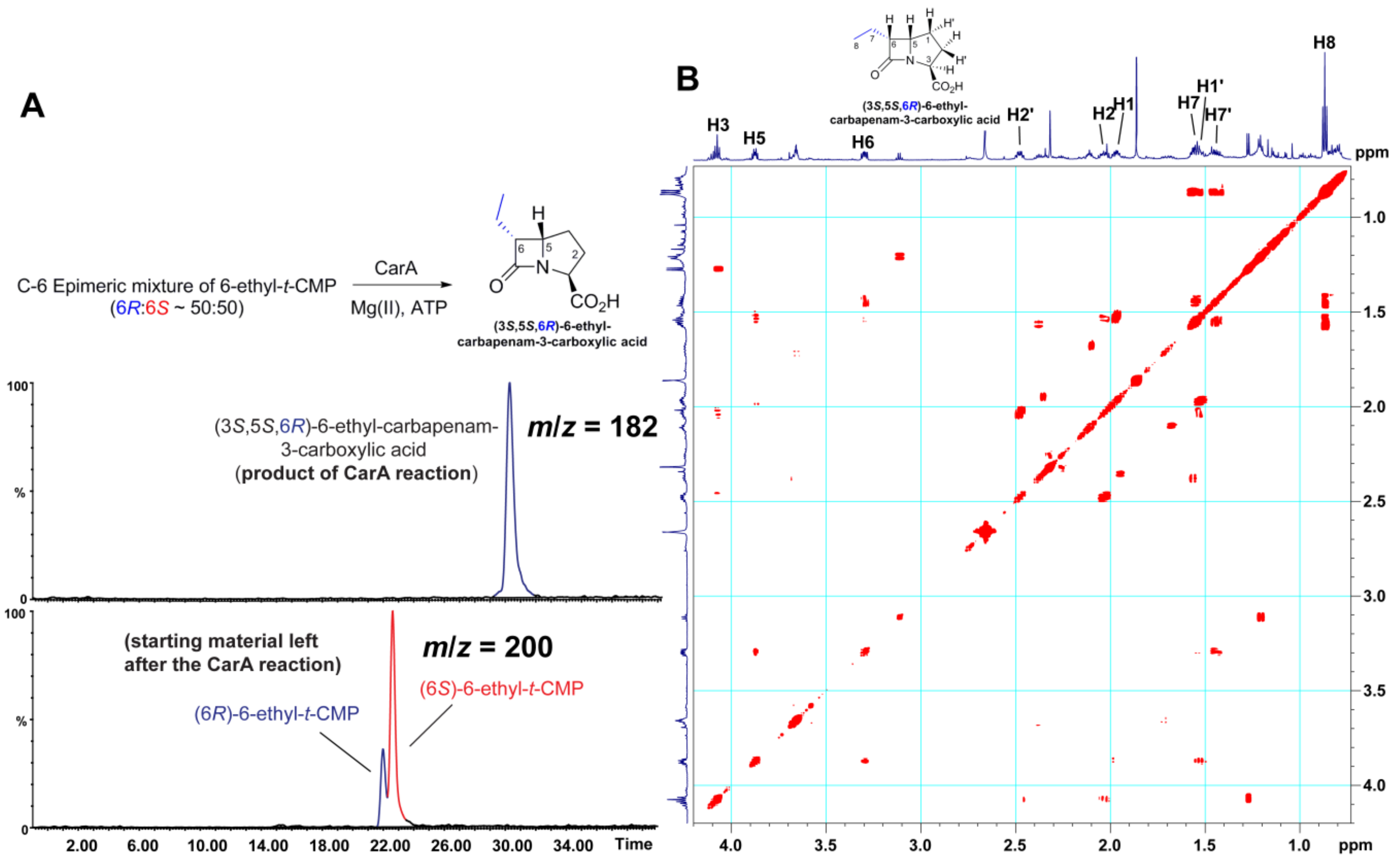

Supplementary Figure 37: Selective production of (3*S*,5*S*,6*R*)-6-ethyl-carbapenam-3-carboxylic acid by CarA catalysis. A: Ion extracted LC-MS chromatograms (ESI-) for the (3*S*,5*S*,6*R*)-6-ethyl-carbapenam-3-carboxylic acid (top chromatogram) produced by incubation of a mixture of 1:1 of the C-6 epimers of 6-ethyl-*t*-CMP with ATP/Mg^{II} and CarA. Note the preference of CarA for the (6*R*)-epimer of 6-ethyl-*t*-CMP as a substrate; **B**: ^1H-^1H COSY spectrum for the purified (3*S*,5*S*,6*R*)-6-ethyl-carbapenam-3-carboxylic acid produced by CarA catalysis. The stereochemistry at C-6 was assigned/further confirmed as (*R*) on the basis of the $J_{5,6}$ = 4.7 Hz (typical for β-lactams with H5 and H6 in a *cis*-relationship).[13]

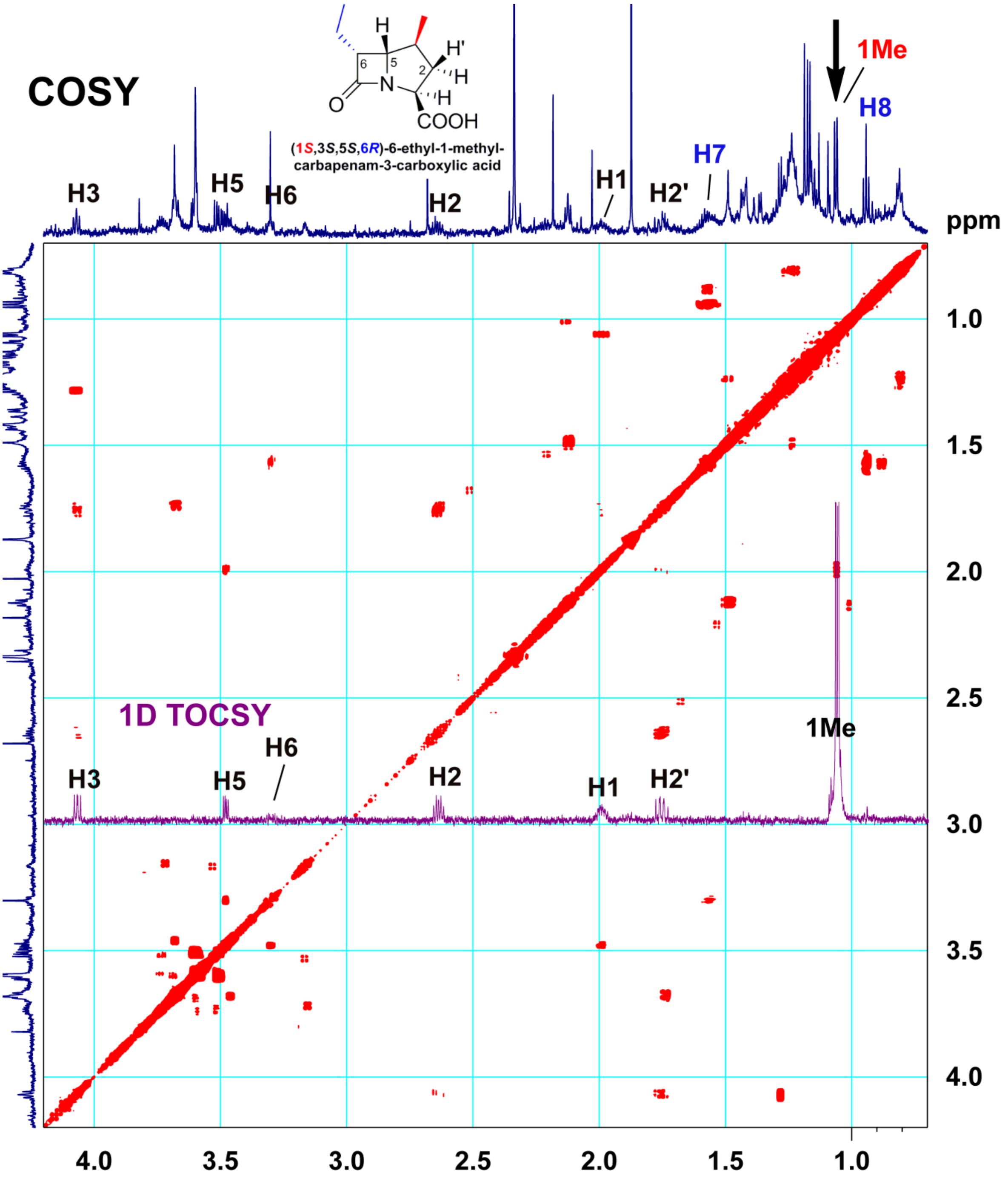

Supplementary Figure 38: NMR spectra for the (1*S*,3*S*,5*S*,6*R*)-6-ethyl-1-methylcarbapenam-3-carboxylic acid produced by incubation of (4*S*,6*R*)-6-ethyl-4-methyl-*t*-CMP with ATP/Mg^{II} and CarA. The 1D-TOCSY spectrum (τ_m = 150 ms) was generated by selective excitation of the protons of the methyl group at C-1. The stereochemistry at C-6 was assigned/confirmed as (*R*) on the basis of the $J_{5,6}$ = 4.9 Hz (typical for β-lactams with H5 and H6 in a *cis*-relationship).[13]

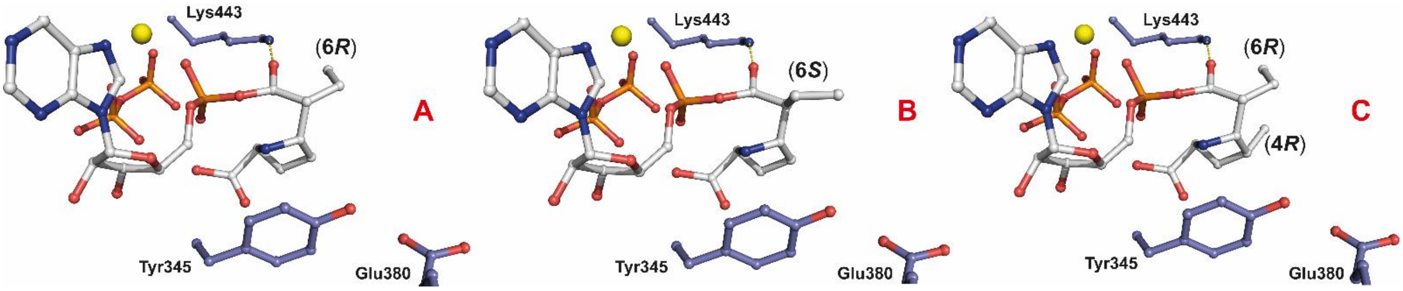

Supplementary Figure 39: Views from models based on a CarA crystal structure (PDB 1Q19)[14] with the proposed acyl-adenylate intermediate of the C-4/C-6 substituted stereoisomers of *t*-CMP derivatives in the active site. Note that the intramolecular Bürgi-Dunitz trajectory for nucleophilic attack of the secondary amine onto the carbonyl of the acyl-adenylate intermediate is predicted to be less sterically hindered in the case of the (6*R*)-epimer (**A**) than the (6*S*)-epimer (**B**) of 6-ethyl-*t*-CMP, potentially rationalising the bias of CarA towards substrates with the (6*R*)-stereochemistry. Similarly, the reactive conformation of the acyl-adenylate intermediate for the (4*R*,6*R*)-4-methyl-6-ethyl-*t*-CMP diastereomer (**C**) is likely to be disfavoured due to a steric clash between the substituents at C-4 and C-6 relative to the (4*S*,6*R*)-diastereomer (not shown). The model is built on the basis of the acyl-adenylate N^2-(2-carboxymethyl)arginine-AMP species observed in a crystal structure of β-LS (PDB 1MBZ).[15] Lys443 is proposed[16] to assist in ring cyclisation *via* stabilisation of the proposed “tetrahedral” intermediate (resulting upon nucleophilic attack by the secondary amine of *t*-CMP). The shown Tyr345-Glu380 dyad is proposed to be involved in deprotonation of the substrate amine in β-lactam formation.[17]

Supplementary References

1 Sambrook, J., Fritsch, E. F. & Maniatis, T. *Molecular cloning: A laboratory manual, 2nd ed.* (Cold Spring Harbor Laboratory Press, Cold Spring Harbor, 1989).

2 Navarro-Vázquez, A., Cobas, J. C., Sardina, F. J., Casanueva, J. & Díez, E. A Graphical Tool for the Prediction of Vicinal Proton−Proton 3JHH Coupling Constants. *J. Chem. Inf. Comput. Sci.* **44**, 1680-1685 (2004).

3 Erb, T. J. *et al.* Synthesis of C_5-dicarboxylic acids from C_2-units involving crotonyl-CoA carboxylase/reductase: The ethylmalonyl-CoA pathway. *Proc. Natl. Acad. Sci. U. S. A.* **104**, 10631-10636 (2007).

4 Hughes, Amanda J. & Keatinge-Clay, A. Enzymatic Extender Unit Generation for In Vitro Polyketide Synthase Reactions: Structural and Func-tional Showcasing of Streptomyces coelicolor MatB. *Chem. Biol.* **18**, 165-176 (2011).

5 Sleeman, M. C. & Schofield, C. J. Carboxymethylproline synthase (CarB), an unusual carbon-carbon bond-forming enzyme of the crotonase superfamily involved in carbapenem biosynthesis. *J. Biol. Chem.* **279**, 6730-6736 (2004).

6 Hamed, R. B. *et al.* Stereoselective C-C bond formation catalysed by engineered carboxymethylproline synthases. *Nat. Chem.* **3**, 365-371 (2011).

7 Hamed, R. B. *et al.* The enzymes of β-lactam biosynthesis. *Nat. Prod. Rep.* **30**, 21-107 (2013).

8 Thompson, J. D., Higgins, D. G. & Gibson, T. J. CLUSTAL W: Improving the sensitivity of progressive multiple sequence alignment through sequence weighting, position-specific gap penalties and weight matrix choice. *Nucl. Acids Res.* **22**, 4673-4680 (1994).

9 Ducho, C. *et al.* Synthesis of regio- and stereoselectively deuterium-labelled derivatives of L-glutamate semialdehyde for studies on carbapenem biosynthesis. *Org. Biomol. Chem.* **7**, 2770-2779 (2009).

10 Hamed, R. B., Mecinovic, J., Ducho, C., Claridge, T. D. W. & Schofield, C. J. Carboxymethylproline synthase catalysed syntheses of functionalised N-heterocycles. *Chem. Commun.* **46**, 1413-1415 (2010).

11 Sorensen, J. L., Sleeman, M. C. & Schofield, C. J. Synthesis of deuterium labelled L- and D-glutamate semialdehydes and their evaluation as substrates for carboxymethylproline synthase (CarB) - implications for carbapenem biosynthesis. *Chem. Commun.*, 1155-1157 (2005).

12 Hamed, R. B., Batchelar, E. T., Mecinovic, J., Claridge, T. D. W. & Schofield, C. J. Evidence that Thienamycin Biosynthesis Proceeds via C-5 Epimerization: ThnE Catalyzes the Formation of (2*S*,5*S*)-*trans*-Carboxymethylproline. *ChemBioChem* **10**, 246-250 (2009).

13 Albers-Schönberg, G. *et al.* Structure and absolute-configuration of thienamycin. *J. Am. Chem. Soc.* **100**, 6491-6499 (1978).

14 Miller, M. T., Gerratana, B., Stapon, A., Townsend, C. A. & Rosenzweig, A. C. Crystal structure of carbapenam synthetase (CarA). *J. Biol. Chem.* **278**, 40996-41002 (2003).

15 Miller, M. T., Bachmann, B. O., Townsend, C. A. & Rosenzweig, A. C. The catalytic cycle of β-lactam synthetase observed by x-ray crystallographic snapshots. *Proc. Natl. Acad. Sci. U. S. A.* **99**, 14752-14757 (2002).

16 Raber, M. L., Castillo, A., Greer, A. & Townsend, C. A. A Conserved Lysine in β-Lactam Synthetase Assists Ring Cyclization: Implications for Clavam and Carbapenem Biosynthesis. *ChemBioChem* **10**, 2904-2912 (2009).

17 Raber, M. L., Arnett, S. O. & Townsend, C. A. A Conserved Tyrosyl−Glutamyl Catalytic Dyad in Evolutionarily Linked Enzymes: Carbapenam Synthetase and β-Lactam Synthetase. *Biochemistry* **48**, 4959-4971 (2009).
